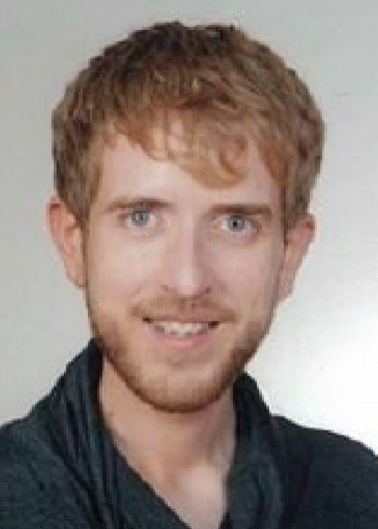# Progress in Difluoroalkylation of Organic Substrates by Visible Light Photoredox Catalysis

**DOI:** 10.1002/adsc.201801121

**Published:** 2019-01-17

**Authors:** Agostinho Lemos, Christian Lemaire, André Luxen

**Affiliations:** ^1^ GIGA Cyclotron Research Centre *In Vivo* Imaging University of Liège Allée du 6 Août 8, 4000 Liège Belgium

**Keywords:** C−H functionalization, difluoroalkylation, late-stage fluorination, organophotocatalysis, transition metal photocatalysis, visible light

## Abstract

The selective incorporation of fluorinated motifs, in particular CF_2_FG (FG=a functional group) and CF_2_H groups, into organic compounds has attrracted increasing attention since organofluorine molecules are of the utmost importance in the areas of nuclear imaging, pharmaceutical, agrochemical, and material sciences. A variety of synthetic approaches has been employed in late‐stage difluoroalkylation reactions. Visible light photoredox catalysis for the production of CF_2_FG and CF_2_H radicals has provided a more sustainable alternative to other conventional radical‐triggered reactions from the viewpoint of safety, cost, availability, and “green” chemistry. A wide range of difluoroalkylating reagents has been successfully implemented in these organic transformations in the presence of transition metal complexes or organic photocatalysts. In most cases, upon excitation *via* visible light irradiation with fluorescent light bulbs or blue light‐emitting diode (LED) lamps, these photocatalysts can act as both reductive and oxidative quenchers, thus enabling the application of electron‐donor or electron‐acceptor difluoroalkylating reagents for the generation of CF_2_FG and CF_2_H radicals. Subsequent radical addition to substrates and additional organic transformations afford the corresponding difluoroalkylated derivatives. The present review describes the distinct strategies for the transition metal‐ and organic‐photocatalyzed difluoroalkylation of a broad range of organic substrates by visible light irradiation reported in the literature since 2014.

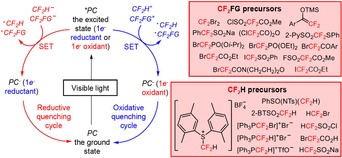

## 1 General Introduction

**Figure 1:**
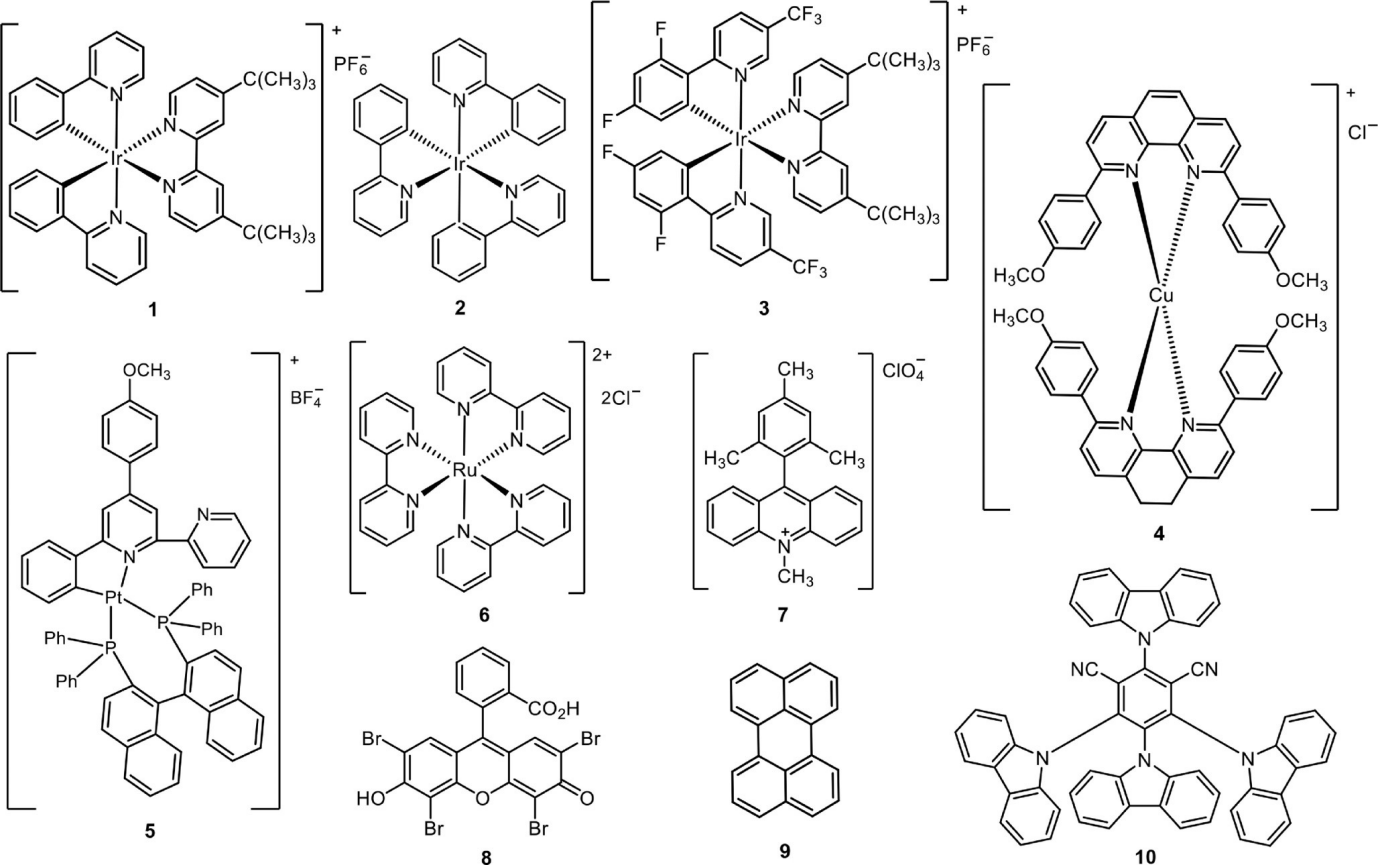
Transition metal (**1**–**6**) and organic photocatalysts (**7**–**10**) employed in difluoroalkylation reactions. **1** – [Ir(dtbbpy)(ppy)_2_]PF_6_; **2** – *fac*‐Ir(III)(ppy)_3_; **3** – [Ir(dF(CF_3_)ppy)_2_(dtbbpy)]PF_6_; **4** – [Cu(dap)_2_]Cl; **5** – Pt(II)[R(C^N^P^P)] (R=4‐CH_3_OC_6_H_4_); **6** – [Ru(bpy)_3_]Cl_2_; **7** – *N*‐methyl‐9‐mesitylacridinium perchlorate ([Mes‐Acr]ClO_4_); **8** – eosin Y; **9** – perylene; **10**‐1,2,3,5‐tetrakis(carbazolyl)‐4,6‐dicyanobenzene (4CzIPN).

**Figure 2:**
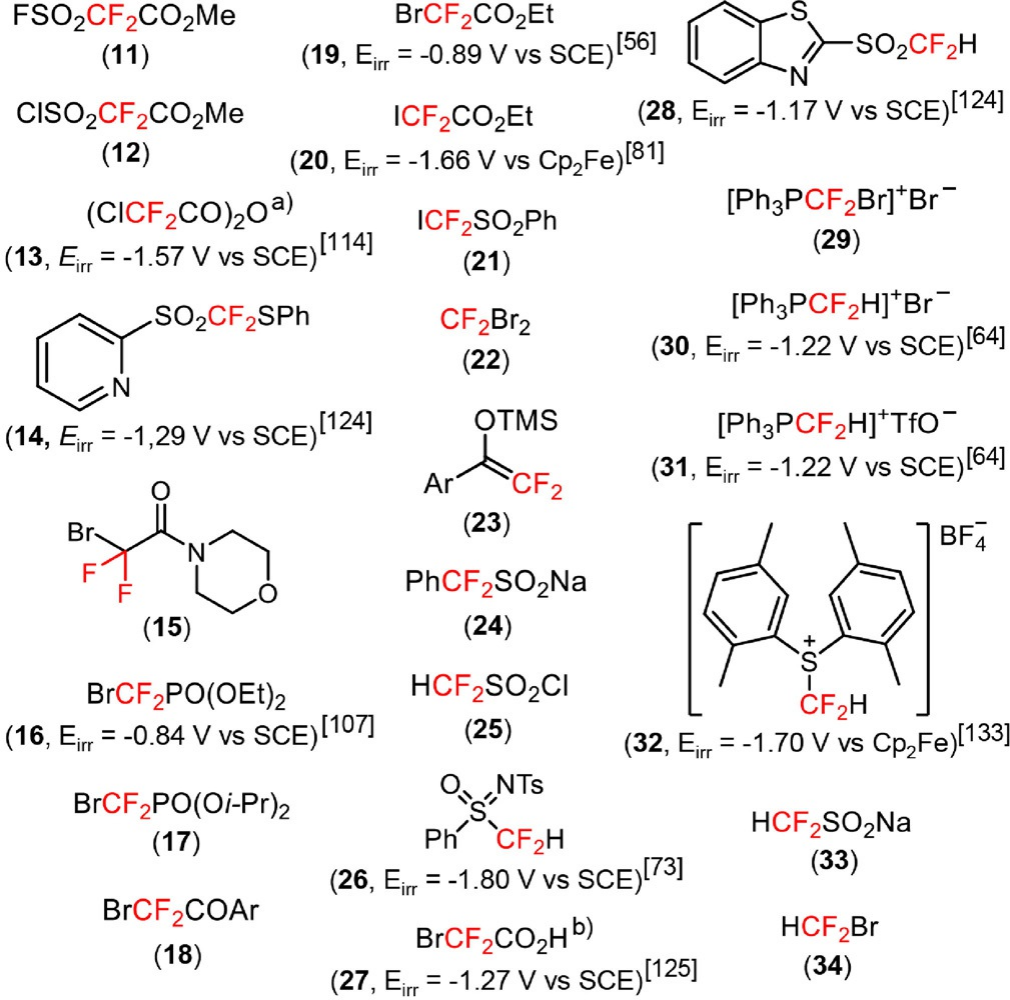
List of CF_2_FG (**11**–**24**) and CF_2_H reagents (**25**–**34**) employed in visible light‐mediated difluoroalkylation reactions and their potentials given in volts *versus* the saturated calomel electrode (SCE) or ferrocene (Cp_2_Fe). ^a)^ Potential of **13** in combination with pyridine *N*‐oxide. ^b)^ Potential of the intermediate BrCF_2_CO_2_Cs.

**Figure 3:**
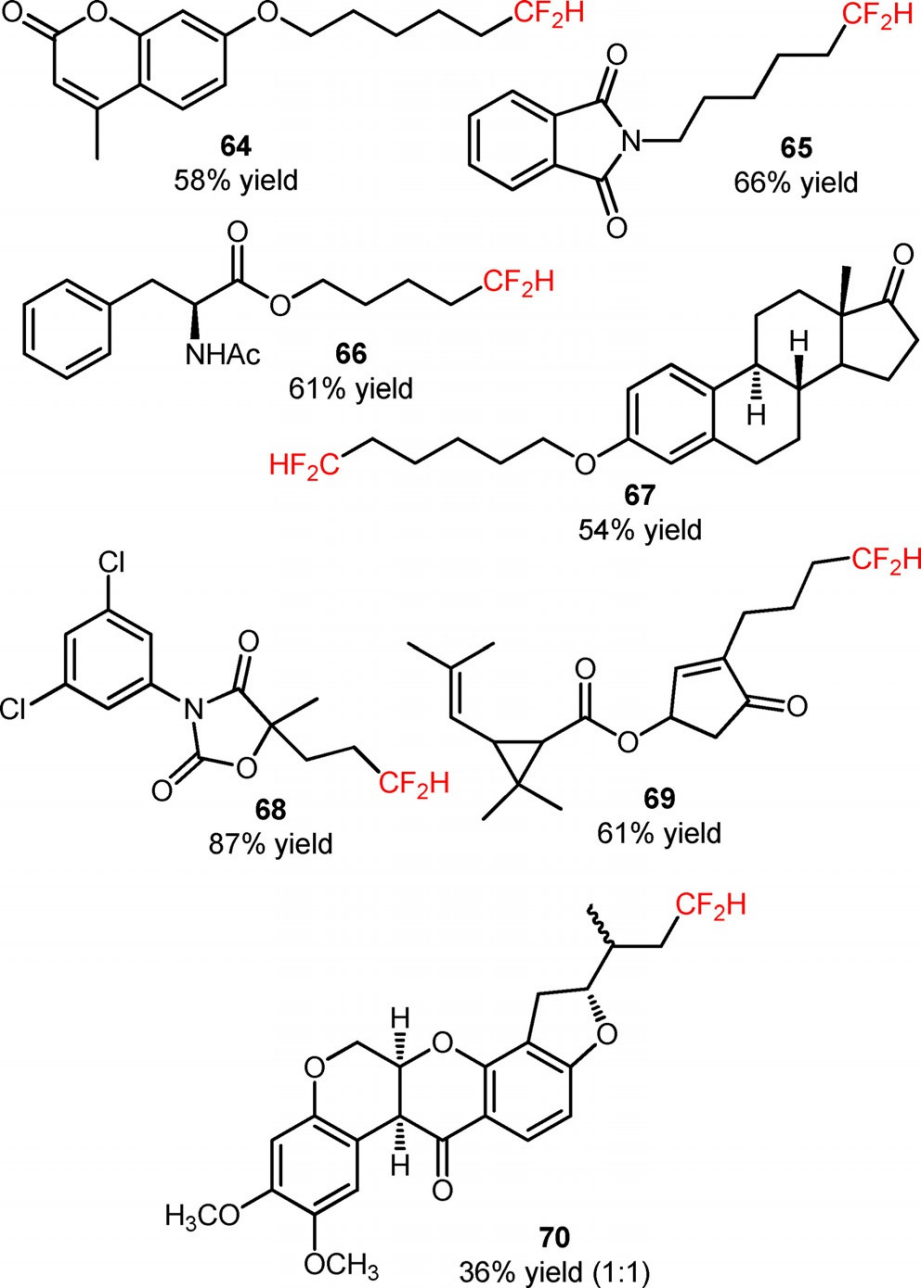
Chemical structures of hydro‐difluoromethylated analogues of 4‐methylumbelliferone (**64**), phthalimide (**65**), l‐phenylalanine (**66**), and estrone (**67**), vinclozolin (**68**), allethrin (**69**), and rotenone (**70**).

Organic compounds containing fluorine substituents or fluoroalkyl moieties are abundant and have attracted considerable attention because of their wide applications in agrochemical,^1^ pharmaceutical,^2^,^3^ and material science^4^ industries, and nuclear imaging.^5^,^6^ In pharmaceutical research and drug development, the incorporation of fluoroalkyl motifs, in particular the difluoromethyl (CF_2_H) group, has gained great interest for use in isostere‐based drug design. As a lipophilic hydrogen‐bond donor,^7^ the CF_2_H substitution offers a viable alternative to conventional hydrogen‐bond donors [e.g., hydroxy (OH), amino (NH_2_), thiol (SH), carbinol (CH_2_OH), amide (CONH_2_), and hydroxamic acid (CONHOH) groups] in terms of lipophilicity, cell membrane permeability, and metabolic stability, thus improving the biological activity.^8^,^9^ Given the relevance of difluoroalkyl substituents in life sciences, the implementation of efficient approaches for the preparation of CF_2_‐containing organic molecules has become a major research area in the field of organofluorine chemistry. Apart from the huge progress in the development of strategies for C−H functionalization involving fluorination and trifluoromethylaton reactions,^10^, ^11^, ^12^, ^13^, ^14^, ^15^, ^16^, ^17^, ^18^ significant research efforts have been directed toward the late‐stage introduction of CF_2_FG (FG=a functional group) and CF_2_H moieties in organic skeletons *via* nucleophilic, electrophilic, and radical approaches.^19^, ^20^, ^21^, ^22^ Among the mentioned approaches for the difluoroalkylation process, the radical‐triggered reactions *via* visible light photoredox catalysis have been the subject of intensive research by the chemical community, owing to their unique advantages such as the use of “green” and environmentally benign reaction conditions, excellent functional group versatility, and high reactivity.^23^, ^24^, ^25^, ^26^, ^27^, ^28^, ^29^, ^30^ In fact, the use of photoredox catalysis has provided a powerful and versatile tool to afford a large variety of fluorinated radicals under very mild conditions, compared with conventional radical reactions that usually demand the use of high‐energy ultraviolet (UV) light equipment or the employment of highly toxic radical initiators. In general, these visible light‐induced chemical transformations rely on the ability of photocatalysts, such as transition metal complexes,^31^, ^32^, ^33^ organic dyes^34^,^35^ or heterogeneous semiconductors^36^,^37^ to promote single‐electron transfer (SET) processes with organic molecules upon excitation with visible light. Remarkably, the lack of visible light absorbance of many organic molecules enables the application of these photocatalysts in these reactions, minimizing the occurence of unwanted side reactions resulting from the photoexcitation and the decomposition of reaction products. Visible light irradiation is often carried out using inexpensive light sources such as blue light‐emitting diode (LED) lamps and fluorescent light bulbs. A variety of transition metal photocatalysts, such as iridium {[Ir(dtbbpy)(ppy)_2_]PF_6_ (**1**, Figure 1), *fac*‐Ir(III)(ppy)_3_ (**2**, Figure 1), and [Ir(dF(CF_3_)ppy)_2_(dtbbpy)]PF_6_ (**3**, Figure 1)}, copper {[Cu(dap)_2_]Cl (**4**, Figure 1)}, platinum {Pt(II)[R(C^N^P^P)] (R=4‐CH_3_OC_6_H_4_) (**5**, Figure 1)}, and ruthenium {[Ru(bpy)_3_]Cl_2_ (**6**, Figure 1)} complexes, and organic photocatalysts, including *N*‐methyl‐9‐mesitylacridinium perchlorate ([Mes‐Acr]ClO_4_) (**7**, Figure 1), eosin Y (**8**, Figure 1), perylene (**9**, Figure 1), and 1,2,3,5‐tetrakis(carbazolyl)‐4,6‐dicyanobenzene (4CzIPN) (**10**, Figure 1) have been implemented in photochemistry for the difluoroalkylation of organic substrates. These photocatalysts are capable of absorbing light at a certain wavelength in the visible region, resulting in the generation of photoexcited species that possess the unique property of being both more oxidizing and more reducing than the species in the ground state. The standard reduction potentials are used to quantify the redox properties of a photocatalyst in the excited state under specific standard conditions (Table 1), and describe the electrochemical potential associated with a half‐reaction (E_1/2_) of reduction. The reduction potential determines the propensity of a chemical species to be reduced. In fact, the more positive the potential values, the greater is the tendency of a molecule to be reduced. For example, *fac*‐Ir(III)(ppy)_3_* is a much more potent electron donor [E_1/2_ (PC^+^/PC*)=−1.73 V *vs*. SCE] than the *fac*‐Ir(III)(ppy)_3_ in the ground state [E_1/2_ (PC^+^/PC)=+0.77 V *vs*. SCE]. Reduction potentials of difluoroalkylating reagents (**11**–**34**, Figure 2) are also highlighted in this review. Depending on the reduction potentials of the photocatalysts in the excited state and the difluoroalkylating reagents, the excited photocatalysts can act as SET reductants or oxidative quenchers (oxidative quenching cycle, OQC) and SET oxidants or reductive quenchers (reductive quenching cycle, RQC), allowing the formation of CF_2_H and CF_2_FG radicals. The resulting oxidized or reduced species will then undergo a second SET reduction or oxidation, respectively, returning the photocatalyst to its initial low‐energy state. The redox potentials of both difluoroalkylating reagents and photocatalysts must be taken into consideration in order to select the most appropriate partners for the design of a photocatalytic difluoroalkylation reaction.^38^,^39^ Subsequent addition of CF_2_FG and CF_2_H radicals in *sp* ^2^‐hybridized (C=C, C=N) and *sp*‐hybridized (C≡C, C≡N) carbon atoms of organic substrates and further chemical transformations would afford the corresponding CF_2_FG‐ and CF_2_H‐containing products. Alternatively, the CF_2_FG moiety of difluoroalkylated derivatives can be converted into other CF_2_‐containing functional groups, including CF_2_H, under certain reaction conditions. Interestingly, the radical difluoroalkylation of key organic molecules can provide useful intermediates for the formation of structurally complex and functionalized heterocycles of pharmaceutical and medical interest. Pioneering works in fluoroalkylation chemistry *via* visible light photoredox catalysis have been reported by MacMillan, Cho, and Sanford. In 2009, MacMillan's group achieved the enantioselective α‐trifluoromethylation and α‐perfluoromethylation of aldehydes with trifluoroiodomethane (CF_3_I) using the readily available [Ir(dtbbpy)(ppy)_2_]PF_6_ and an imidazolinone catalyst.^40^ Later, in 2011, the same group developed photoredox‐based protocols for the α‐trifluoromethylation of enol silanes, silylketene acetals and *N*,*O*‐acetals derived from ketones, esters, and amides using CF_3_I^41^ and for the trifluoromethylation of arenes as well as five‐, and six‐membered heteroarenes with trifluoromethanesulfonyl chloride (CF_3_SO_2_Cl), in the presence of [Ru(bpy)_3_]Cl_2_ and [Ru(phen)_3_]Cl_2_, respectively.^42^ In 2012, Cho and collaborators described a procedure for the trifluoromethylation of electron‐rich heterocycles *via* [Ru(bpy)_3_]Cl_2_ photocatalysis.^43^ In the same year, Sanford's group reported the trifluoromethylation and perfluoroalkylation of arylboronic acids by merging photoredox and copper catalysis.^44^ Since then, photoinduced fluoroalkylation reactions have mostly relied on the incorporation of trifluoromethyl (CF_3_) groups in organic substrates. Seminal works in visible light‐induced difluoroalkylation chemistry were reported in 2014 and, to date, a myriad of difluoroalkylating reagents (**11**–**34**, Figure 2) has been successfully implemented for structurally diverse organic molecules. Remarkably, one of the most critical challenges of late‐stage difluoroalkylation compared to trifluoromethylation is that the replacement of one electronegative fluorine atom in CF_3_‐containing reagents by a hydrogen atom or by other functional groups may induce a signficant diminution of the reduction potentials. For instance, the generation of CF_2_H radicals from electrophilic CF_2_H precursors requires the use of more strongly reducing catalysts when compared with the case of CF_3_ radicals.

**Table 1:** Redox potentials and photophysical properties of transition metal (**1–6**) and organic photocatalysts (**7–10**) utilized in difluoroalkylation reactions.^[a]^ (PC=a photocatalyst).

PC	E_1/2_ (PC^+^/PC*)	E_1/2_ (PC*/PC^−^)	E_1/2_ (PC^+^/PC)	E_1/2_ (PC/PC^−^)	Excited‐state lifetime *τ* [ns]	Excitation *λ*_max_ [nm]	Emission *λ*_max_ [nm]	Refs.
**1**	−0.96	+0.66	+1.21	−1.51	557	410	581	54,55
**2**	−1.73	+0.31	+0.77	−2.19	1900	375	494^[b]^	50
**3**	−0.89	+1.21	+1.69	−1.37	2300	380	470	55
**4**	−1.43		+0.62		270	400–600	670^[c]^	62
**5**	−1.90^[d]^	+0.82^[d]^	+0.61^[d]^	−1.69^[d]^	93^[d]^	350^[d]^	543^[d]^	81
**6**	−0.81	+0.77	+1.29	−1.33	1100	452	615	79,80
**7**		+2.06		−0.57	6.4	430	570	134, 135, 136, 137
**8**	−1.11^[e]^	+0.83^[e]^	+0.78^[e]^	−1.06^[e]^	24000^[e]^	539^[e]^		127
**9**	−2.23	+0.72	+0.61	−2.12	8.2	407, 434	670	133
**10**	−1.04	+1.35	+1.52	−1.21	5100	435	535	140, 141, 142

^[a]^ All potentials are given in volts *versus* the saturated calomel electrode (SCE). Measurements were performed in MeCN at room temperature unless otherwise noted. ^[b]^ Determined in 1:1 EtOH/MeOH at 77 K. ^[c]^ Determined in DCM. ^[d]^ Potentials are given in volts *versus* the ferrocene (Cp_2_Fe). ^[e]^ Determined in 1:1 MeCN/H_2_O.

Numerous reviews in fluoroalkylation chemistry have emphazised the various synthetic approaches for visible light‐mediated trifluoromethylation and other perfluoroalkylation reactions.^38^,^39^,^45^,^46^,^47^ Although some of these reviews have covered the area of difluoroalkylation chemistry in part,^46^,^47^ a review focusing exclusively on the incorporation of CF_2_H and CF_2_FG groups under visible light photoredox conditons will be convenient due to the increasing interest in the formed difluoroalkylated products in life sciences. In addition, major breakthroughs have been accomplished in this research field since the first reported works in 2014. Herein, the present review highlights the distinct synthetic strategies for transition metal‐ and organic‐photocatalyzed difluoroalkylation of a broad range of organic substrates by visible light irradiation that have been reported in the literature since 2014. Owing to the attractive characteristics of visible light photoredox catalysis and the late‐stage introduction of difluoroalkyl groups, we expect that the present review will inspire organic chemists to explore additional synthetic routes for installation of these moieties.

### 1.1 Transition Metal Photocatalyzed Difluoroalkylation Reactions

The importance of transition metal complexes as effective SET reductants and oxidants, upon excitation *via* irradiation with visible light, has been demonstrated by the considerable number of research works that were reported recently, involving the incorporation of CF_2_FG and CF_2_H moieties in a variety of substrates bearing unsaturated bonds, including C=C and C=N, and the concomitant formation of new C−C bonds. In most cases, iridium transition metal complexes have proven to be priviliged photocatalysts in the difluoroalkylation of unactivated alkenes, styrenes, enol derivatives, allylic alcohols, and α,β‐unsaturated carboxylic acids, arenes, and heteroarenes.

### 1.2 Difluoroalkylation of *sp* ^2^ Carbon Atoms in Unactivated Alkenes and Styrenes

**Scheme 1:**
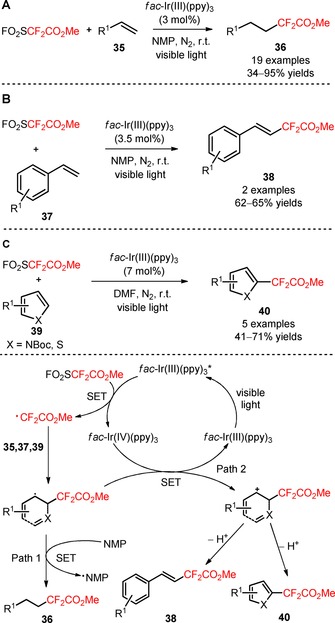
Methoxycarbonyldifluoromethylation of alkenes (**35**), styrenes (**37**), and heteroarenes (**39**) by visible light photoredox catalysis and the suggested mechanism.

The commercially available and easy‐to‐handle methyl 2,2‐difluoro‐2‐(fluorosulfonyl)acetate (FSO_2_CF_2_CO_2_Me, Chen's reagent, CAS number: 680‐15‐9) has been exclusively employed for the preparation of trifluoromethylated derivatives.^48^,^49^ Qing and collaborators disclosed the application of FSO_2_CF_2_CO_2_Me for the installation of CF_2_CO_2_Me substituents in alkenes (**36**, Scheme 1A: 19 examples, 34–95% yields), styrenes (**38**, Scheme 1B: 2 examples, 62–65% yields), and heteroarenes (**40**, Scheme 1C: 5 examples, 41–71% yields) under visible light photoredox conditions, in the presence of *fac*‐Ir(III)(ppy)_3._ 50 A plausible reaction mechanism involved the formation of CF_2_CO_2_Me radicals from the reduction of FO_2_SCF_2_CO_2_Me *via* oxidative quenching of *fac*‐Ir(III)(ppy)_3_* and the loss of SO_2_ and F^−^. The entrapment of these radicals by alkenes (**35**), styrenes (**37**), and heteroarenes (**39**) afforded the corresponding difluoroalkylated intermediates. The resulting radical intermediates can undergo two distinct pathways depending on the substrates. For alkenes (**35**), hydrogen abstraction of the radical intermediate from NMP gave the hydro‐difluoroalkylated alkanes (**36**). For styrenes (**37**) and heteroarenes (**39**), the oxidation of the radical intermediate and subsequent deprotonation provided the methoxycarbonyldifluoromethylated products (**38** and **40**).^51^

**Scheme 2:**
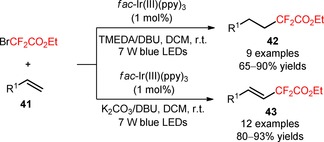
Hydro‐difluoroalkylation and alkenyl‐difluoroalkylation of unactivated alkenes (**41**) under visible light photoredox conditions in the presence of *fac*‐Ir(III)(ppy)_3_.

The difluoroalkylating reagent ethyl 2‐bromo‐2,2‐difluoroacetate (BrCF_2_CO_2_Et, CAS number: 565‐53‐7) was also implemented by Cho's group for the difluoroalkylation of unactivated alkenes (**41**).^52^ Interestingly, the authors found that the selection of the bases and solvents was critical for guiding the chemoselective synthesis of difluoroalkylated alkanes and alkenes. In fact, difluoroalkylated alkanes were preferentially obtained using a mixture of bases DBU/TMEDA in DCM (**42**, Scheme 2: 9 examples, 65–90% yields). On the other hand, the formation of difluoroalkyl‐containing alkenes with high levels of regio‐ and *E*/*Z* stereoselectivity (90–97%) was achieved by complete conversion of the aliphatic alkenes and styrenes to the bromodifluoroalkylated products using the base K_2_CO_3_ and subsequent dehydrobromination with DBU in DMF (**43**, Scheme 2: 12 examples, 80–93% yields).

**Scheme 3:**
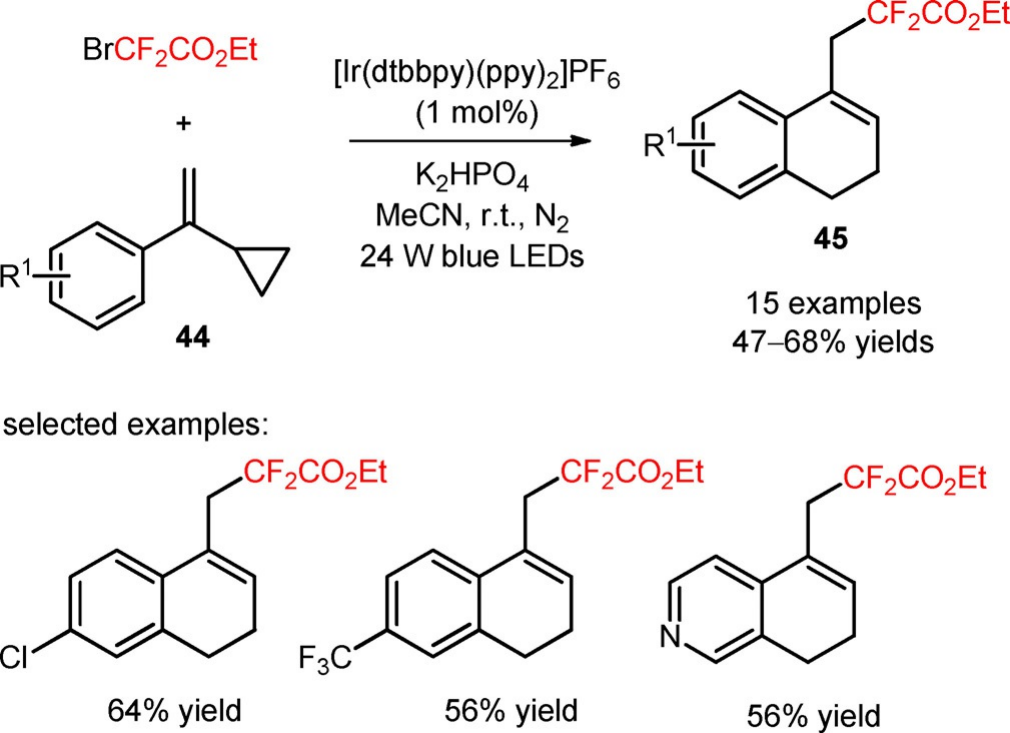
Photoinduced difluoroalkylation of α‐cyclopropylstyrenes and α‐cyclopropylpyridines (**44**).

Partially hydrogenated naphthalenes and quinolines containing difluoroalkyl moieties (**45**) were efficiently prepared *via* radical difluoroalkylation of α‐cyclopropylstyrenes and α‐cyclopropylpyridines (**44**) with BrCF_2_CO_2_Et, respectively, opening of cyclopropyl ring, and consecutive annulation reaction.^53^ In the presence of [Ir(dtbbpy)(ppy)_2_]PF_6,_ 54,55 a wide range of α‐cyclopropyl olefins bearing electron‐donating and electron‐withdrawing groups regioselectively afforded the corresponding products with moderate yields (**45**, Scheme 3: 15 examples, 47–68% yields). The developed methodology can be extended to other brominated compounds including bromodifluoroacetamides, ethyl 2‐bromo‐2‐fluoroacetate (BrCHFCO_2_Et), 2‐bromoacetonitrile, and diethyl 2‐bromomalonate.

**Scheme 4:**
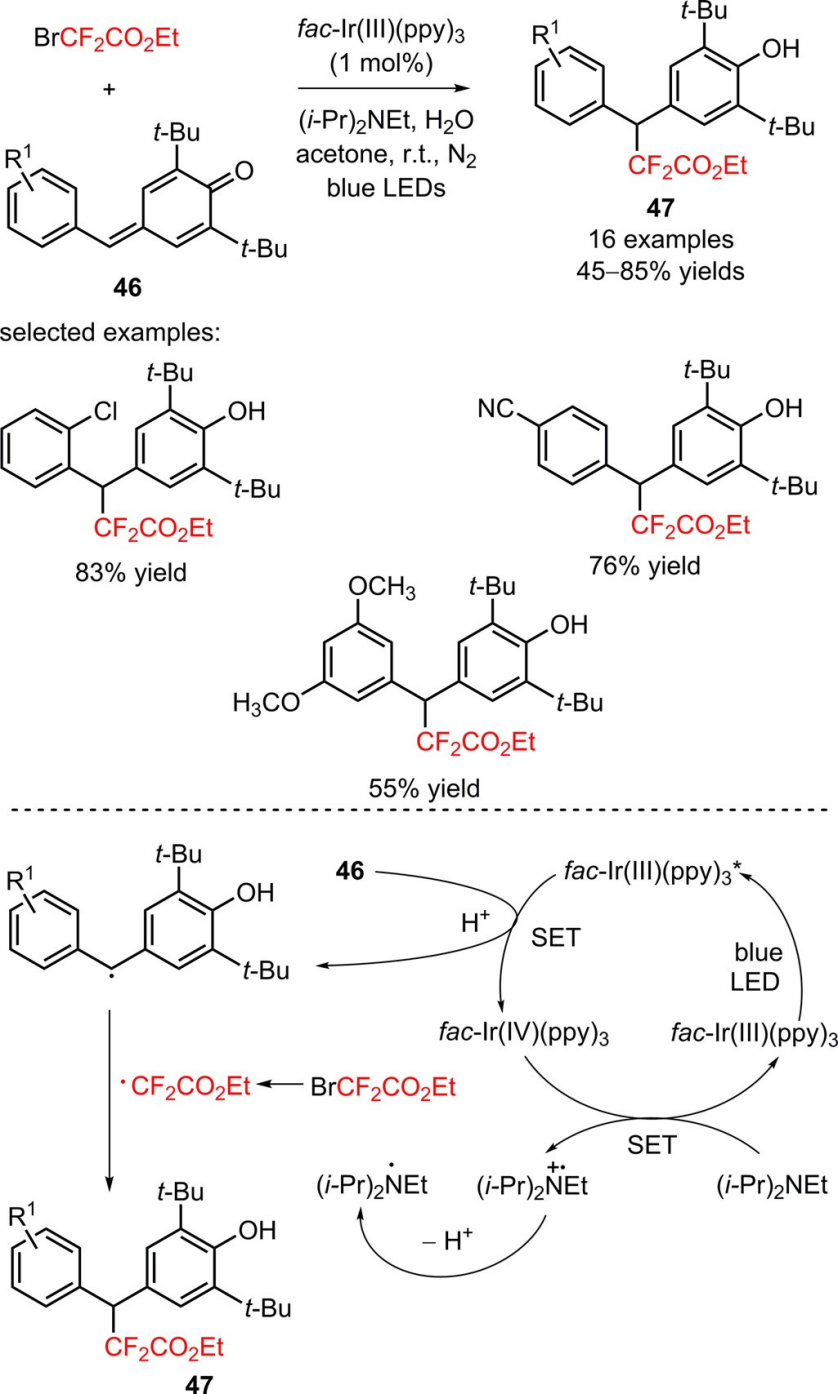
Visible light‐induced radical‐radical cross‐coupling difluoroalkylation of *para*‐quinone methides (**46**).

Xu and collaborators reported a new approach to access difluoroalkylated diarylmethanes (**47**) from *para*‐quinone methides (**46**) and BrCF_2_CO_2_Et *via* radical‐radical cross‐coupling, under irradiation with blue LEDs.^56^ In the presence of *fac*‐Ir(III)(ppy)_3_, the inclusion of H_2_O and the reductant (*i‐*Pr)_2_NEt in the reaction system was beneficial for the difluoroalkylation process. *para*‐Quinone methides bearing electron‐withdrawing and electron‐donating groups on the aromatic ring provided the corresponding products with moderate to excellent yields. Disubstitution with chloro and methoxy groups was also well tolerated (**47**, Scheme 4: 16 examples, 45–85% yields). Remarkably, the developed strategy can be implemented using other difluoroalkylating reagents with acylamino, carbonyl, esteryl, and heteroaryl substituents. Stern–Volmer fluorescence quenching studies and radical‐trapping experiments suggested the formation of diarylmethyl radicals *via* oxidative quenching of *fac*‐Ir(III)(ppy)_3_* species and the CF_2_CO_2_Et radicals *via* oxidation of the (*i‐*Pr)_2_NEt radical intermediate. Cross‐coupling between diarylmethyl and CF_2_CO_2_Et radicals afforded the difluoroalkylated diarylmethanes (**47**).

**Scheme 5:**
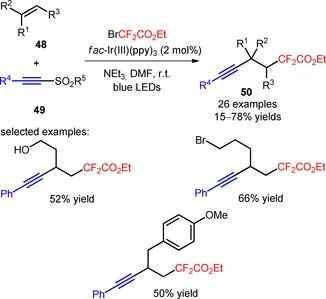
Visible light‐induced three‐component alkynyl‐difluoroalkylation of unactivated alkenes (**48**), BrCF_2_CO_2_Et, and alkynyl sulfones (**49**).

Recently, Zhu and co‐workers described a novel tactic for the intermolecular alkynyl‐difluoroalkylation of unactivated alkenes (**48**) *via* a three‐component condensation with BrCF_2_CO_2_Et and alkynyl sulfones (**49**), under visible light photoredox conditions.^57^ The combined use of *fac*‐Ir(III)(ppy)_3_ with the DMF and the base NEt_3_ was critical for the selective formation of β‐difluoroalkylated alkynes (**50**), minimizing the unwanted bromine addition and direct difluoroalkylation of the alkynyl sulfones (**49**). Terminal and internal alkenes with a variety of functional groups (**48**), and alkynyl sulfones bearing aryl and heteroaryl substituents (**49**) were all suitable substrates for the alkynyl‐difluoroalkylation process (**50**, Scheme 5: 26 examples, 15–78% yields). Bromodifluoroacetamides can also provide the corresponding β‐fluoroalkylated alkynes under the developed methodology.

**Scheme 6:**
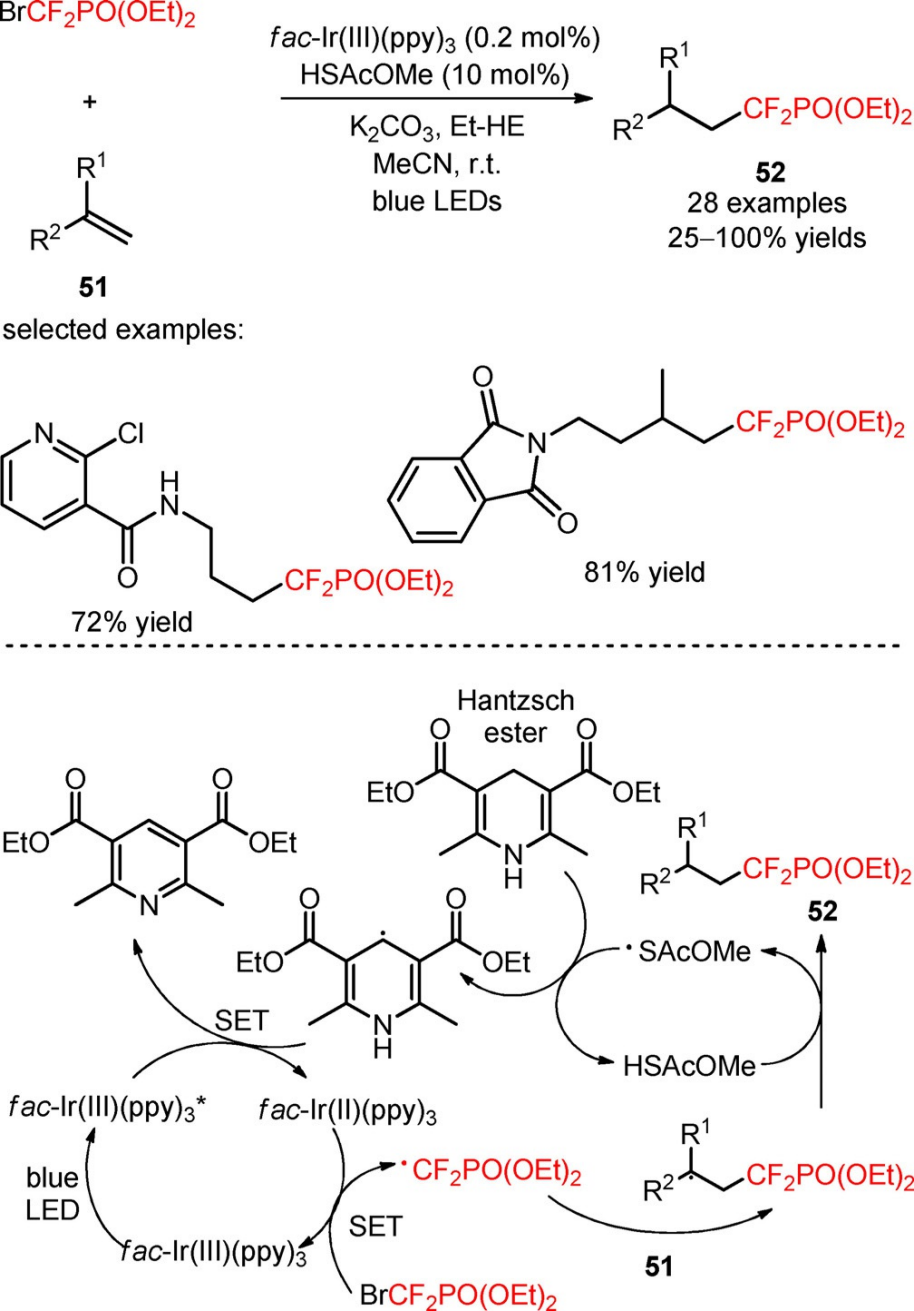
Hydro‐phosphonodifluoromethylation of alkenes (**51**) *via* thiyl radical/photoredox catalysis.

Diethyl (bromodifluoromethyl)phosphonate [(BrCF_2_PO(OEt)_2_, CAS number: 65094‐22‐6] was implemented by Li and co‐workers in the hydro‐phosphonodifluoromethylation of alkenes (**51**) using the Hantzsch ester Et‐HE as a hydrogen source and the thiyl radical precursor HSAcOMe, under irradiation with blue LEDs.^58^ The authors found that combining the thiyl radical‐catalyzed hydrogen atom transfer ability of the HE with RQC may avoid the use of strongly basic conditions and block the undesirable halogen atom transfer addition pathway *via* OQC. In the presence of *fac*‐Ir(III)(ppy)_3_, mono‐ and disubstituted alkenes bearing electron‐rich and electron‐deficient aromatic groups as well as heterocyclic and aliphatic groups (**51**) were compatible substrates with the hydro‐difluoroalkylation process (**52**, Scheme 6: 28 examples, 25–100% yields). This procedure was applied to the single‐step synthesis of the intermediate of a purine nucleoside phosphorylase (PNP) inhibitor. Mechanistic studies with radical scavengers and alternative reductants suggested a reductive quenching of *fac*‐Ir(III)(ppy)_3_*, and consecutive generation of CF_2_PO(OEt)_2_ radicals *via* reduction of BrCF_2_PO(OEt)_2_. Radical difluoroalkylation of the alkenes (**51**) followed by hydrogen atom transfer between HSAcOMe and the difluoroalkylated radical intermediate afforded the corresponding products (**52**).

**Scheme 7:**
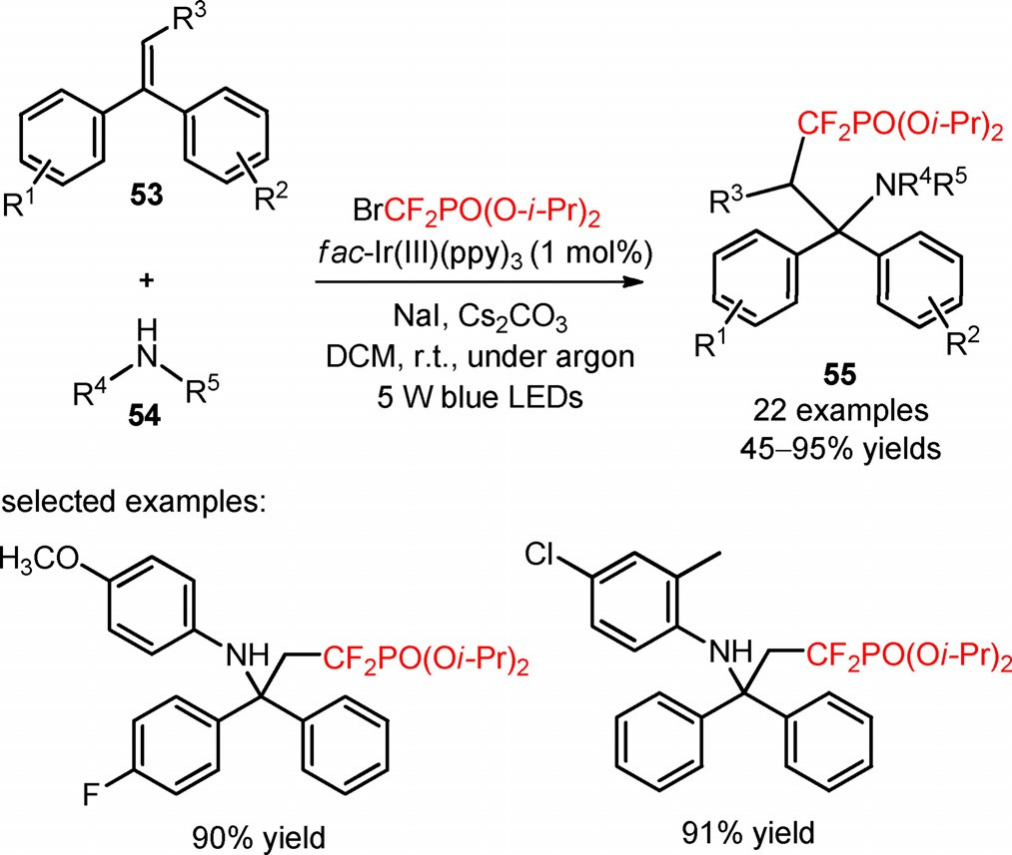
Photocatalyzed intermolecular amino‐phosphonodifluoromethylation of alkenes (**53**).

The synthesis of α,α‐difluoro‐γ‐aminophosphonates (**55**) was described by Qiang and collaborators through intramolecular amino‐phosphonodifluoromethylation of diarylalkenes (**53**) with diisopropyl (bromodifluoromethyl)phosphonate [BrCF_2_PO(O‐*i‐*Pr)_2_, CAS number: 65094‐24‐8] under irradiation with 5 W blue LEDs.^59^ A palette of electron‐rich and electron‐deficient diarylalkenes (**53**), and arylamines (**54**) can be effectively converted into the difluoroalkylated products (Scheme 7, **55**, 22 examples, 45–95% yields). Interestingly, this procedure was applied to the synthesis of phosphonodifluoromethylated chiral binaphthylamines using (*R*)‐(+)‐1,1′‐binaphthyl‐2,2′‐diamine [(*R*)‐BINAM] and (*R*)‐(+)‐2′‐amino‐1,1′‐binaphthalen‐2‐ol [(*R*)‐NOBIN] as the substrates, and of the α,α‐difluoro‐γ‐aminophosphoric acid. Radical‐trapping and light on/off experiments suggested the intermediacy of CF_2_PO(O‐*i‐*Pr)_2_ radicals *via* oxidative quenching of *fac*‐Ir(III)(ppy)_3_*.

**Scheme 8:**
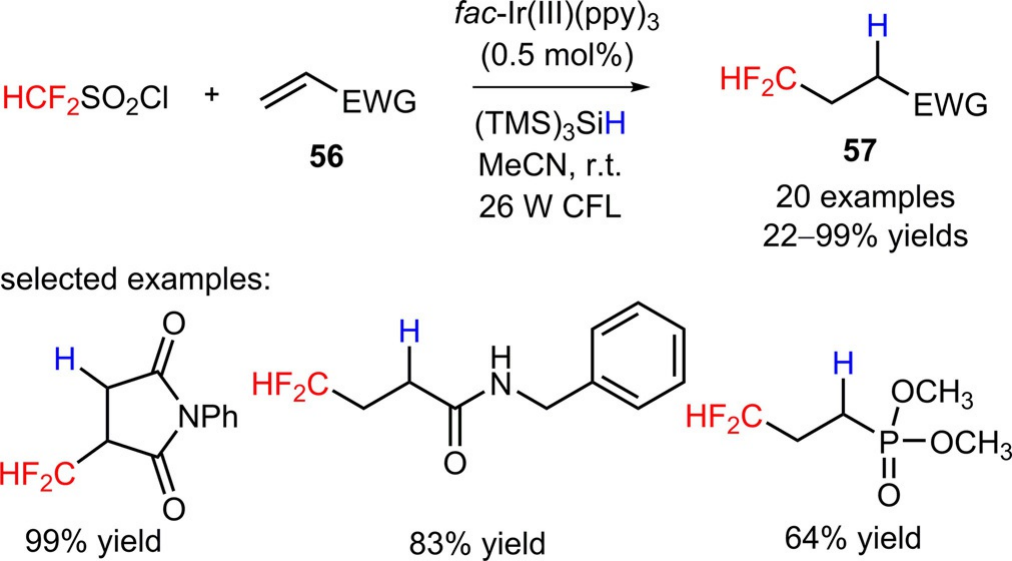
Visible light‐catalyzed hydro‐difluoromethylation of electron‐deficient alkenes (**56**) with HCF_2_SO_2_Cl.

Dolbier's group developed a novel strategy for the hydro‐difluoromethylation of alkenes (**56**) bearing a large variety of electron‐withdrawing groups with the reagent difluoromethanesulfonyl chloride (HCF_2_SO_2_Cl, CAS number: 1512‐30‐7) as the source of CF_2_H radicals, under irradiation with 26 W compact fluorescent lamp (CFL) (**57**, Scheme 8: 20 examples, 22–99% yields).^60^ In addition to the photocatalyst *fac*‐Ir(III)(ppy)_3_, the introduction of tris(trimethylsilyl)silane [(TMS)_3_SiH] with hydrogen atom donor properties was pivotal for the direct hydro‐difluoromethylation of alkenes, circumventing the formation of chloro‐difluoromethylated products. Other difluoroalkylating compounds, including (bromodifluoromethyl)benzene (PhCF_2_Br), 1,1‐difluoroethane‐1‐sulfonyl chloride (CH_3_CF_2_SO_2_Cl), and 2‐azido‐1,1‐difluoroethane‐1‐sulfonyl chloride (N_3_CH_2_CF_2_SO_2_Cl) can be implemented in the hydro‐difluoroalkylation of the substrates under the described reaction conditions.

**Scheme 9:**
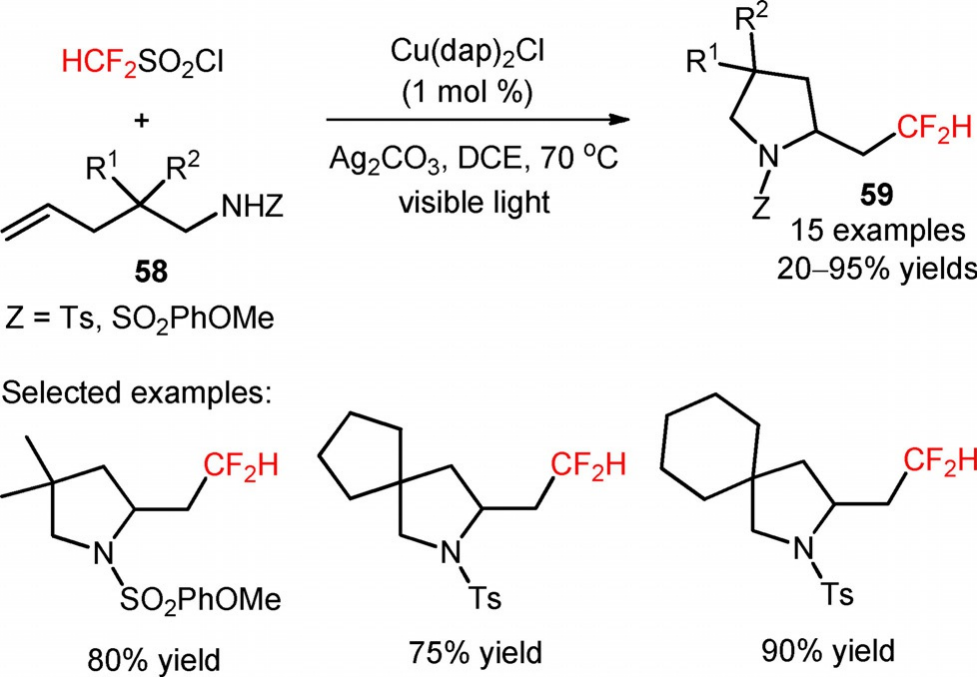
Visible light‐induced difluoromethylation of sulfonamides and esters (**58**) and subsequent radical cyclization in the presence of [Cu(dap)_2_]Cl.

The reagent HCF_2_SO_2_Cl was also efficiently employed in the preparation of difluoromethylated pyrrolidines and lactones through installation of difluoromethyl groups in sulfonamides and esters (**58**), respectively, and subsequent radical cyclization by visible light photoredox catalysis (**59**, Scheme 9: 15 examples, 20–95% yields).^61^ The implementation of [Cu(dap)_2_]Cl^62^ as photocatalyst and the base Ag_2_CO_3_ was crucial for supression of the chloro‐difluoromethylation process.

**Scheme 10:**
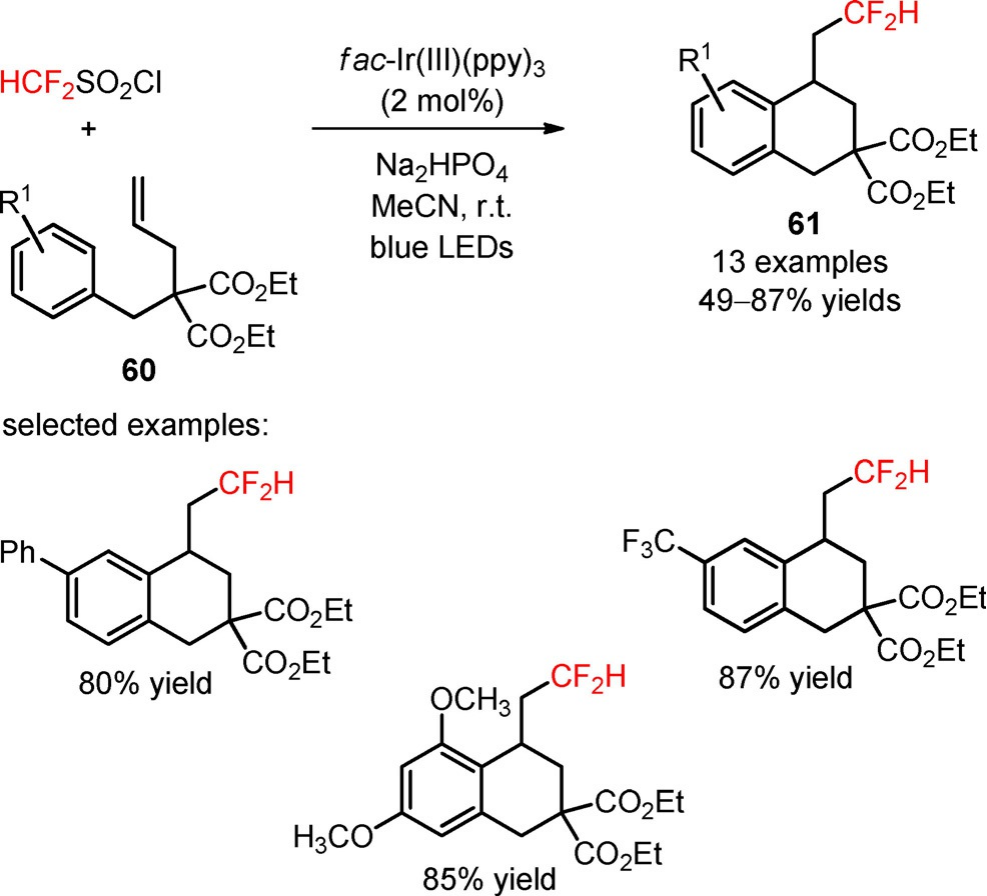
Visible light‐catalyzed difluoromethylation/6‐*exo* cyclization of unactivated alkenes (**60**) with HCF_2_SO_2_Cl.

Alkenes containing *gem*‐dialkoxycarbonyl substituents (**60**) were employed as substrates for photoinduced intramolecular difluoromethylation using the reagent HCF_2_SO_2_Cl, in the presence of *fac*‐Ir(III)(ppy)_3._ 63 A spectrum of difluoromethylated tetralin derivatives possessing electron‐donating and electron‐withdrawing groups in the aromatic ring and alkyl substituents at the β‐position was efficiently obtained in moderate to good yields (**61**, Scheme 10: 13 examples, 49–87% yields). Alternative difluoroalkyl R_f_X radical precursors [*R*_f_=CF_2_CH_3_, CF_2_CO_2_Et, CF_2_CONHPh, CF_2_CON(CH_2_CH_2_)_2_O; X=SO_2_Cl, Br] were also compatible with the developed synthetic methodology.

**Scheme 11:**
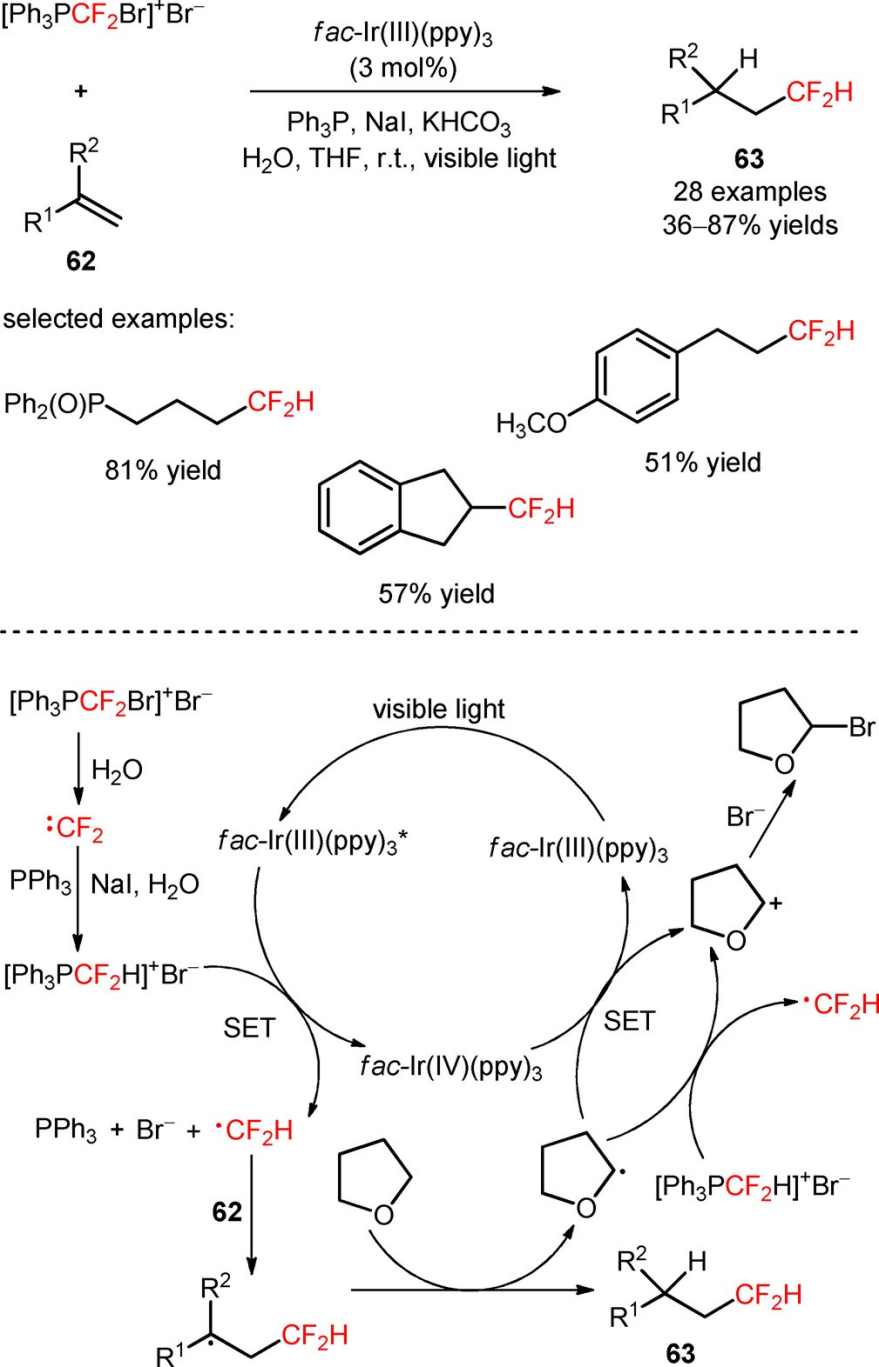
Hydro‐difluoromethylation of unactivated alkenes (**62**) with [Ph_3_PCF_2_Br]^+^Br^−^ under visible light photoredox conditions.

Qing and co‐workers developed a methodology for visible light‐driven hydro‐difluoromethylation of alkenes (**62**) with the easy‐to‐handle (bromodifluoromethyl)triphenylphosphonium bromide [(Ph_3_PCF_2_Br)^+^Br^−^] for the insertion of CF_2_H groups, in the presence of H_2_O and THF.^64^ The reagent (Ph_3_PCF_2_Br)^+^Br^−^ was recognized exclusively as a difluorocarbene precursor.^65^, ^66^, ^67^, ^68^, ^69^ Nevertheless, the authors found that (Ph_3_PCF_2_Br)^+^Br^−^ can be implemented as a CF_2_Br donor, under visible light irradiation. The additional formation of hydro‐difluoromethylated derivatives was solely observed in the presence of the photocatalyst *fac*‐Ir(III)(ppy)_3_. The authors suggested the formation of (difluoromethyl)triphenylphosphonium bromide [(Ph_3_PCF_2_H)^+^Br^−^] resulting from the reaction between (Ph_3_PCF_2_Br)^+^Br^−^ and H_2_O to explain the unexpected hydro‐difluoromethylation. Interestingly, the presence of H_2_O, PPh_3_, NaI, and KHCO_3_ in the reaction medium was critical for selective synthesis of hydro‐difluoromethylated alkanes (**63**). Terminal and internal alkenes bearing various functional groups (**62**) were compatible with the desired organic transformation, affording the hydro‐difluoromethylated products in moderate to high yields (**63**, Scheme 11: 28 examples, 36–87% yields). In addition, this synthetic approach can be extended to more structurally complex substrates such as analogues of 4‐methyl‐umbelliferone (**64**, Figure 3), phthalimide (**65**, Figure 3), l‐phenylalanine (**66**, Figure 3), and estrone (**67**, Figure 3), as well as to biologically active compounds, including the fungicide vinclozolin (**68**, Figure 3) and the two insecticides allethrin (**69**, Figure 3) and rotenone (**70**, Figure 3). Isotopic mechanistic experiments involving D_2_O and THF‐*d*_8_ demonstrated that both H_2_O and THF were the sources of hydrogen atoms for the hydro‐difluoromethylation process. The authors proposed a mechanism of oxidative quenching of *fac*‐Ir(III)(ppy)_3_* and concomitant reduction of (Ph_3_PCF_2_H)^+^Br^−^ to CF_2_H radicals. Electrophilic addition of CF_2_H radicals to the alkenes (**62**) and subsequent abstraction of a hydrogen atom from THF afforded the respective hydro‐difluoromethylated derivatives (**63**).

**Scheme 12:**
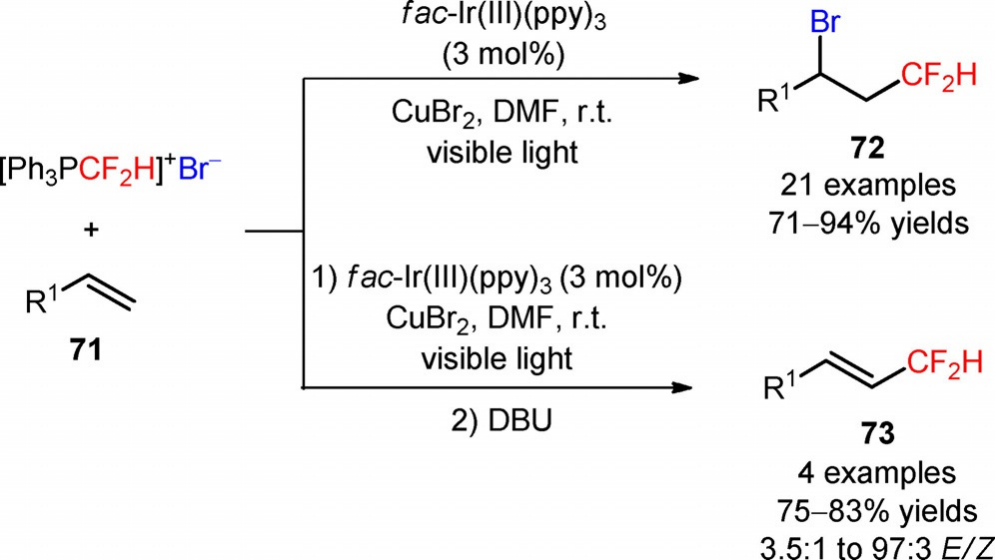
Visible light‐induced photocatalytic bromo‐difluoromethylation and direct difluoromethylation of alkenes (**71**) with [Ph_3_PCF_2_H]^+^Br^−^.

**Figure 4:**
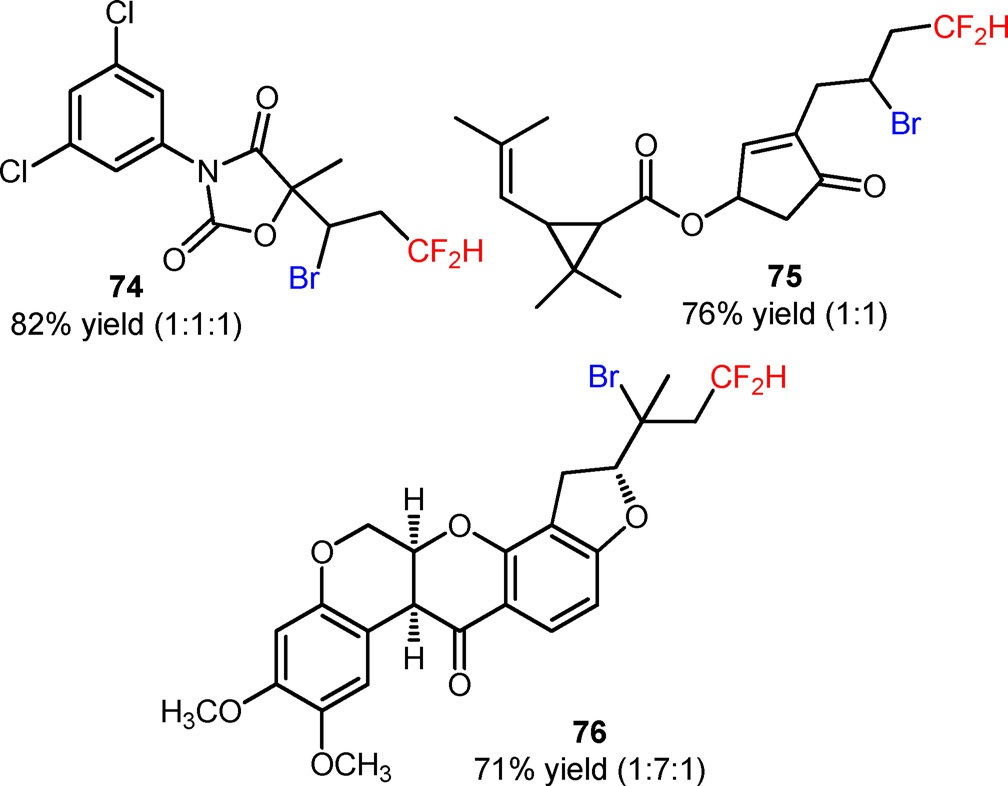
Chemical structures of bromo‐difluoromethylated vinclozolin (**74**), allethrin (**75**), and rotenone (**76**).

Later, the same group described the application of (Ph_3_PCF_2_H)^+^Br^−^ in the bromo‐difluoromethylation of alkenes (**71**) under visible light photoredox conditions.^70^ The use of catalytic amounts of *fac*‐Ir(III)(ppy)_3_ and CuBr_2_ allowed the selective preparation of bromo‐difluoromethylated alkanes (**71**), suppressing the unwanted hydro‐difluoromethylation of the substrates (**72**, Scheme 12: 21 examples, 71–94% yields). The protocol was also applied to the direct bromo‐difluoromethylation of more complex and biologically active molecules, including the fungicide vinclozolin (**74**, Figure 4) and the insecticides allethrin (**75**, Figure 4) and rotenone (**76**, Figure 4). Difluoromethylated alkenes were achieved *via* a one‐pot bromo‐difluoromethylation/dehydro‐bromination process (**73**, Scheme 12: 4 examples, 75–83% yields).

**Scheme 13:**
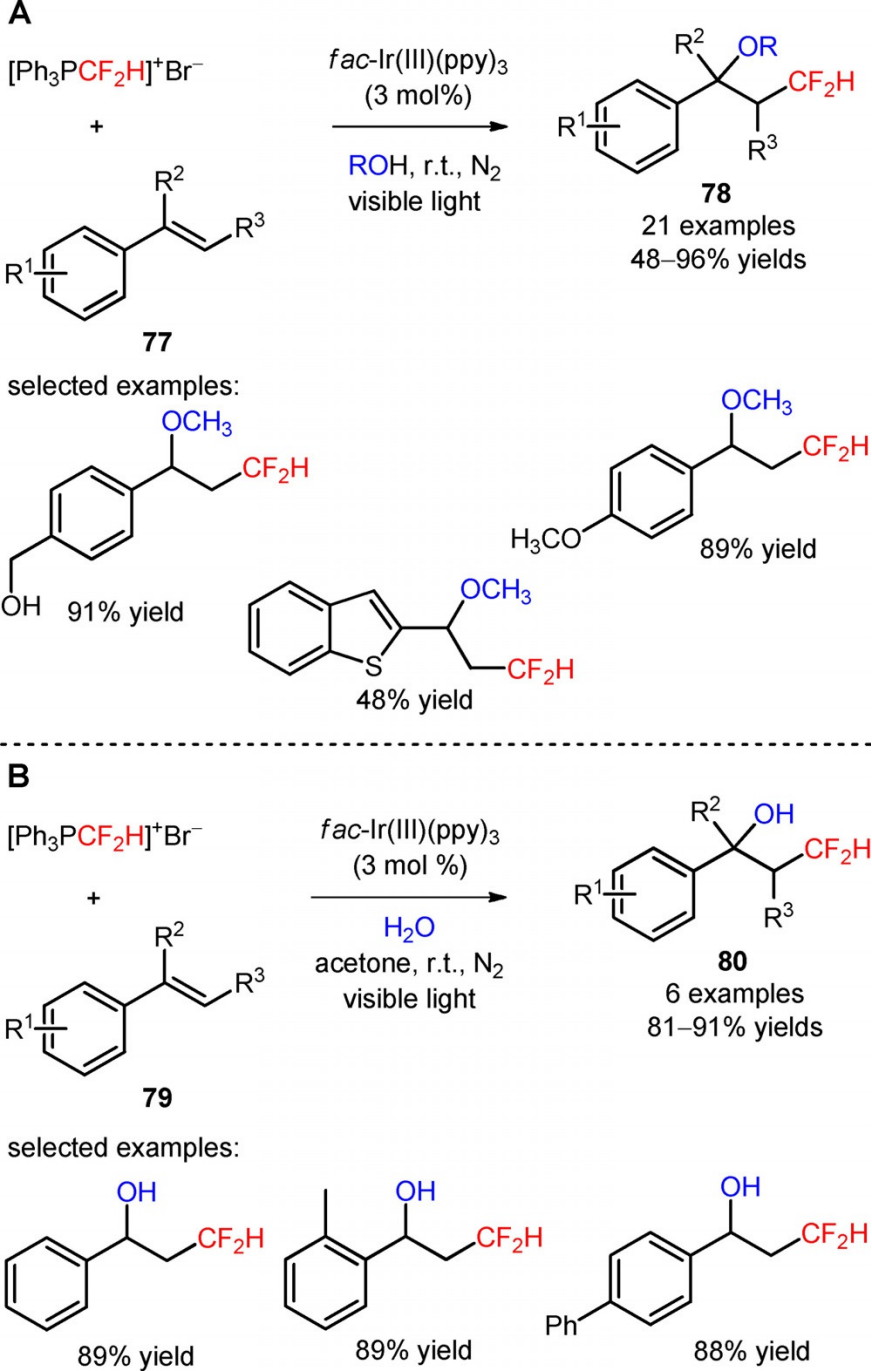
Visible light‐induced oxy‐difluoromethylation of styrenes with [Ph_3_PCF_2_H]^+^Br^−^ using alcohol derivatives (**A**) and water (**B**).

**Figure 5:**
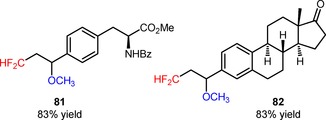
Chemical structures of the products of oxy‐difluoromethylation of vinyl‐*N*‐benzoyl‐l‐tyrosine ethyl ester (**81**) and vinylestrine (**82**).

Difluoromethylated ethers and alcohols were efficiently obtained *via* visible light‐mediated oxy‐difluoromethylation of styrenes (**77**, **79**) with the difluoromethylating reagent [Ph_3_PCF_2_H]^+^Br^−^ using alcohol derivatives and water, respectively, as nucleophiles (**78**, Scheme 13A: 21 examples, 48–96% yields; **80**, Scheme 13B: 6 examples, 81–91% yields).^71^ The protocol was applicable to the late‐stage oxy‐difluoromethylation of vinyl‐*N*‐benzoyl‐l‐tyrosine ethyl ester (**81**, Figure 5) and vinylestrine (**82**, Figure 5).

**Scheme 14:**
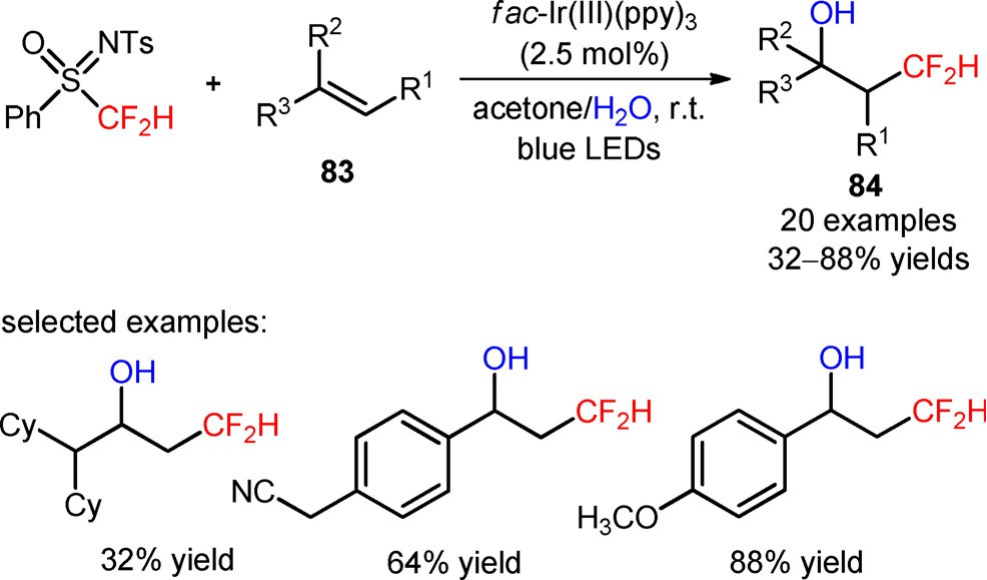
Oxy‐difluoromethylation of alkenes (**83**) using *N*‐tosyl‐*S*‐difluoromethyl‐*S*‐phenylsulfoximine and H_2_O in the presence of *fac*‐Ir(III)(ppy)_3_.

**Figure 6:**
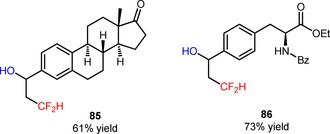
Chemical structures of the products of oxy‐difluoromethylation of vinylestrone (**85**) and vinyl‐*N*‐benzoyl‐l‐tyrosine ethyl ester (**86**).

The shelf‐stable and easy‐to‐handle *N*‐tosyl‐*S*‐difluoromethyl‐*S*‐phenylsulfoximine (CAS number: 1097192‐99‐8, so‐called Hu's reagent), with the electron‐withdrawing sulfoximine group was initially conceived as a difluorocarbene source for the introduction of CF_2_H groups to C‐, N‐, and S‐nucleophiles.^72^ Recently, it has been found that this reagent can also be implemented as a precursor of CF_2_H radicals under photoredox conditions. In fact, Akita and co‐workers reported an efficient protocol to achieve the oxy‐difluoromethylation of alkenes and styrenes (**83**) using *N*‐tosyl‐*S*‐difluoromethyl‐*S*‐phenylsulfoximine and the nucleophile H_2_O under irradiation with blue LEDs.^73^ In the presence of *fac*‐Ir(III)(ppy)_3_, a broad range of difluoromethylated alcohols containing electron‐donating and electron‐withdrawing groups was sucessfully synthesized (**84**, Scheme 14: 20 examples, 32–88% yields). Moreover, structurally complex alkenes, such as vinylestrone (**85**, Figure 6) and vinyl‐*N*‐benzoyl‐l‐tyrosine ethyl ester (**86**, Figure 6), as well as other oxygen nucleophiles, such as alcohols and carboxylic acids, were also compatible with the described oxy‐difluoromethylation strategy. Mechanistic experiments with the radical scavenger 2,2,6,6‐tetramethylpiperidine *N*‐oxide (TEMPO) suggested the intermediacy of CF_2_H radicals *via* oxidative quenching of *fac*‐Ir(IV)(ppy)_3_*.

### 1.3 Difluoroalkylation of *sp* ^2^ Carbon Atoms in Enol Derivatives, α,β‐Unsaturated Carboxylic Acids, and Allylic Alcohols

**Scheme 15:**
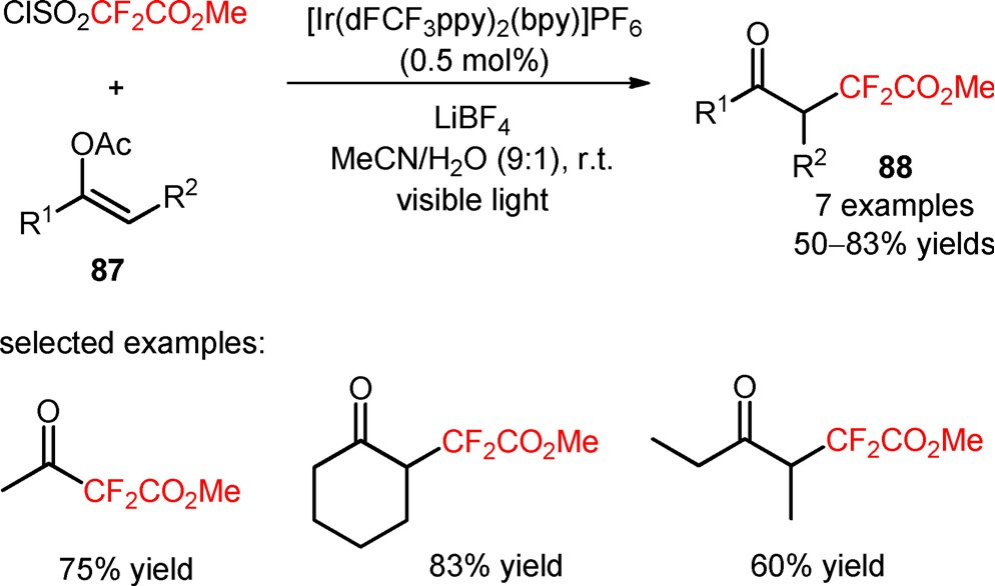
Methoxycarbonyldifluoromethylation of enol acetates (**87**) by visible light photoredox catalysis.

Dolbier and collaborators described a methodology for the visible light‐mediated insertion of methoxycarbonyldifluoromethyl groups in enol acetates (**87**) with methyl 2,2‐difluoro‐2‐(chlorosulfonyl)acetate (ClSO_2_CF_2_CO_2_Me, CAS number: 18225‐68‐8).^74^ A wide array of 2,2‐difluoro‐γ‐keto esters was efficiently prepared in moderate to very good yields (**88**, Scheme 15: 7 examples, 50–83% yields) using the catalyst Ir{[dF(CF_3_)ppy]_2_(dtbbpy)}PF_6_ 55 and the additive LiBF_4_ to enable removal of the acetyl groups.

**Scheme 16:**
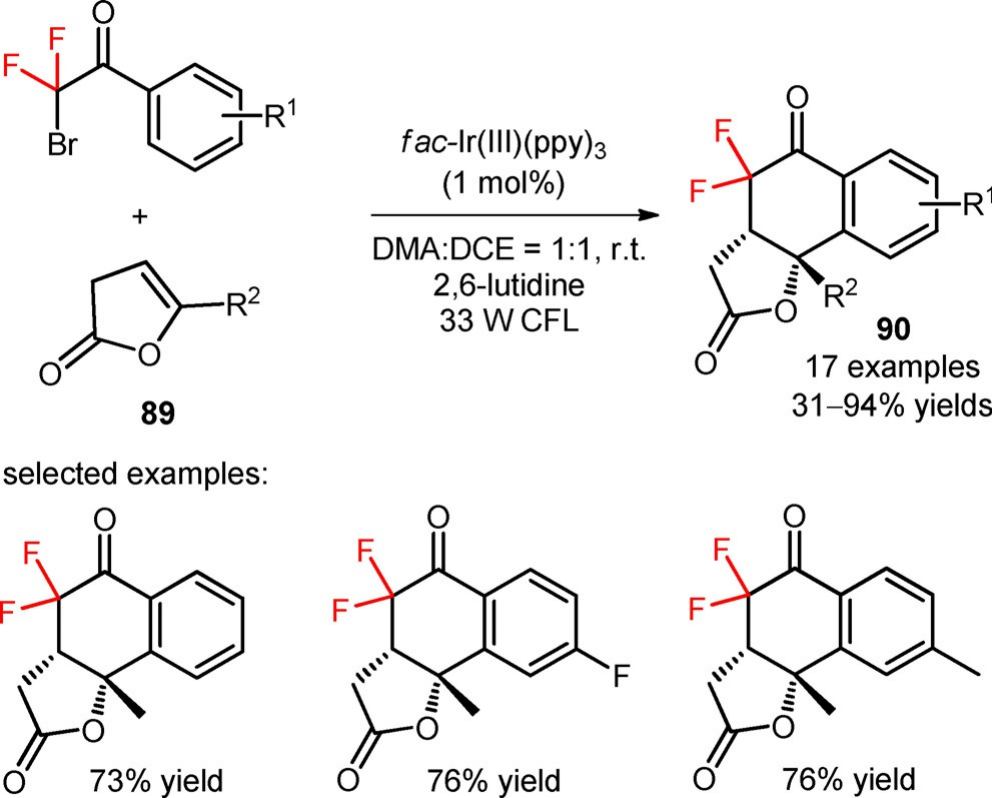
Visible light‐mediated photocatalytic difluoroalkylation of 2‐oxo‐2,3‐dihydrofuran derivatives (**89**) with α‐bromo‐α,α‐difluoroacetophenones. CFL=compact fluorescent lamp.

Difluoroalkylated polycyclic lactones (**90**) were synthesized by radical difluoroalkylation of 2‐oxo‐2,3‐dihydrofuran derivatives (**89**) with α‐bromo‐α,α‐difluoroacetophenones and a consecutive annulation reaction, under irradiation with 33 W fluorescent light bulbs.^75^ The strategy of cascade difluoroalkylation/annulation was efficiently performed in the presence of *fac*‐Ir(III)(ppy)_3_, the base 2,6‐lutidine, and using a solvent mixture of DMA and DCE in a ratio of 1:1 (Scheme 16). Alkyl‐substituted enol lactones (**89**), and *α*‐bromo‐*α*,*α*‐difluoroacetophenones bearing electron‐rich and electron‐withdrawing groups on the aromatic ring provided a wide range of annulated difluoroalkyl‐containing products with an excellent diastereoselectivity (only *cis* products were generated) in moderate to excellent yields (**90**, 17 examples, 31–94% yields).

**Scheme 17:**
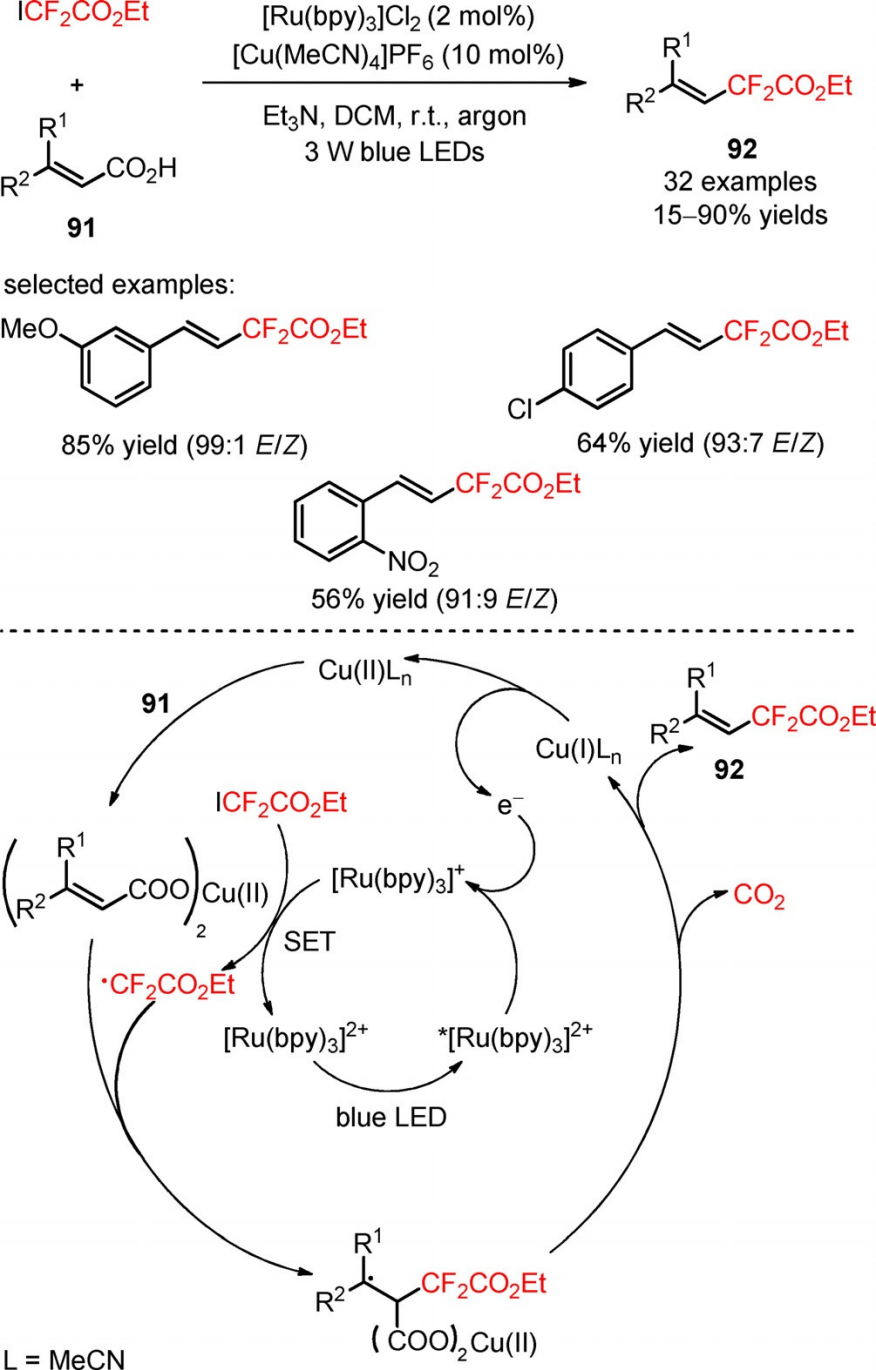
Photoredox‐ and copper‐catalyzed decarboxylative difluoroalkylation of α,β‐unsaturated carboxylic acids (**91**) with ICF_2_CO_2_Et and the proposed mechanism.

α,β‐Unsaturated carboxylic acids have been used as substrates for decarboxylative difluoroalkylation under transition metal catalysis.^76^,^77^ Visible light‐driven methodologies using these substrates have been described by several groups. In 2016, Liu and co‐workers have developed a methodology for the decarboxylative functionalization of α,β‐unsaturated carboxylic acids (**91**) with the difluoroalkylating reagent ethyl 2,2‐difluoro‐2‐iodoacetate (ICF_2_CO_2_Et, CAS number: 7648‐30‐8) by using a dual‐catalytic system merging photocatalysis and copper catalysis.^78^ The photocatalyst [Ru(bpy)_3_]Cl_2,_ 79,80 the copper catalyst [Cu(MeCN)_4_]PF_6_, and the solvent DCM constituted the selected conditions for the difluoroalkylation reaction (Scheme 17). A wide array of α,β‐unsaturated carboxylic acids possessing electron‐rich and electron‐deficient (hetero)aromatic groups gave the corresponding difluoroalkylated styrenes with high *E*/*Z* selectivity in moderate to excellent yields (**92**, 32 examples, 15–90% yields). The authors hypothesized a mechanism involving a reductive quenching of *[Ru(bpy)_3_]^2+^ *via* [Cu(MeCN)_4_]^+^. Subsequent reduction of ICF_2_CO_2_Et to CF_2_CO_2_Et radicals led to the regeneration of the photocatalyst in its ground state. Electrophilic radical addition to the α‐position of the double bond in the substrates followed by elimination of CO_2_ and [Cu(MeCN)_4_]^+^ afforded the difluoroalkylated styrenes (**92**).

**Scheme 18:**
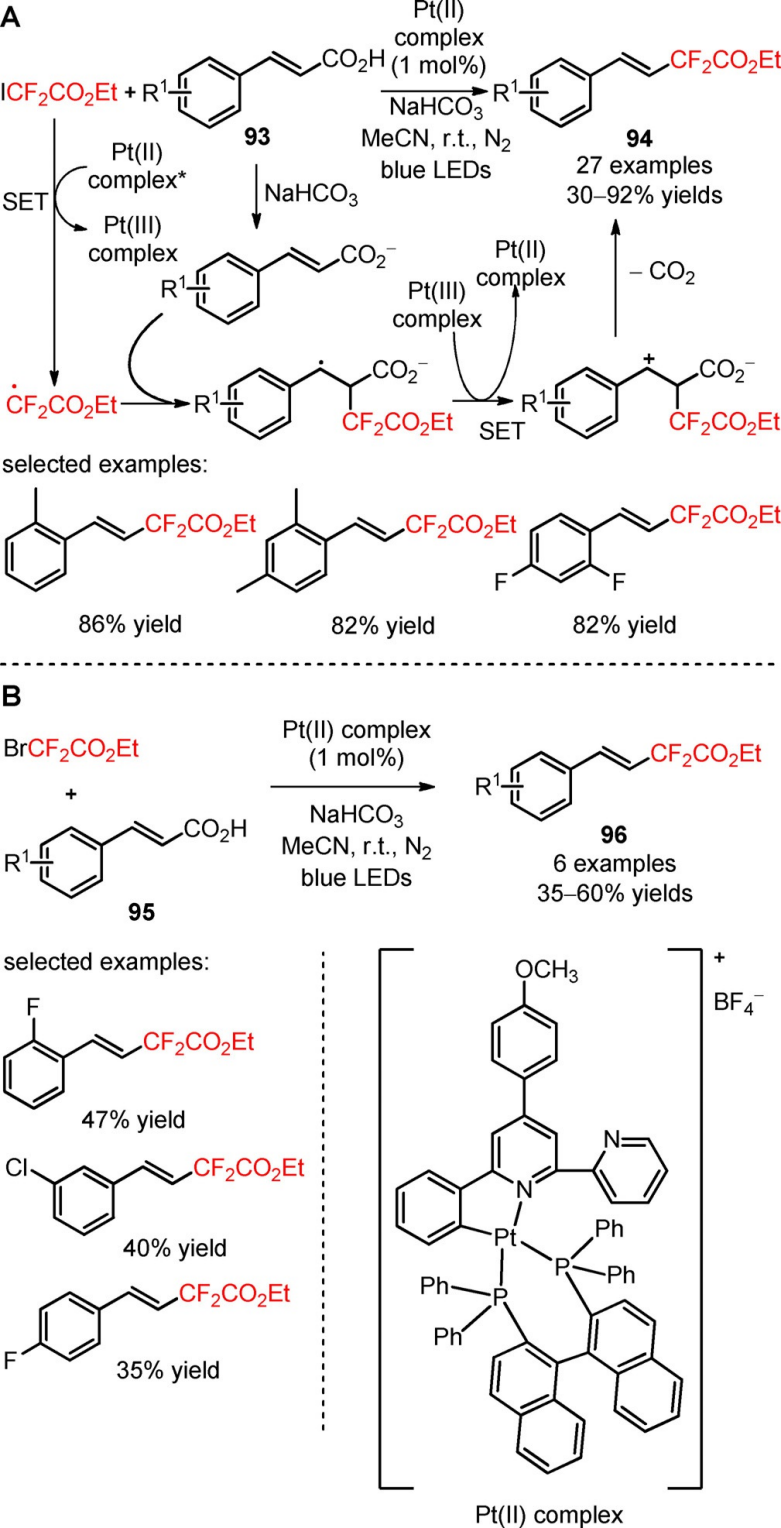
Platinum‐catalyzed difluoroalkylation of α,β‐unsaturated carboxylic acids (**93**, **95**) with the reagents ICF_2_CO_2_Et (**A**) and BrCF_2_CO_2_Et (**B**).

The application of the platinum photocatalyst Pt(II)[R(C^N^P^P)] (R=4‐CH_3_OC_6_H_4_) was described as an alternative approach for the construction of *E*‐difluoroalkylstyrenes from reaction between α,β‐unsaturated carboxylic acids (**93**) and ICF_2_CO_2_Et, under irradiation with blue LEDs (**94**, Scheme 18A: 27 examples, 30–92% yields).^81^ A mechanism for the difluoroalkylation mediated by oxidation of the Pt(II) complex and formation of CF_2_CO_2_Et radicals was proposed along with an initial deprotonation of the α,β‐unsaturated carboxylic acids (**93**) by NaHCO_3_. The developed methodology for the difluoroalkylation of α,β‐unsaturated carboxylic acids (**95**) can also be performed using the reagent BrCF_2_CO_2_Et (**96**, Scheme 18B: 6 examples, 35–60% yields). In the presence of *N*,*N*‐diisopropylethylamine (DIPEA), the reagent ICF_2_CO_2_Et can also be used for the synthesis of difluoroalkyl‐containing alkenyl iodides and *Z*‐difluoroalkylstyrenes *via* photoinduced difluoroalkylation of terminal arylalkynes.

**Scheme 19:**
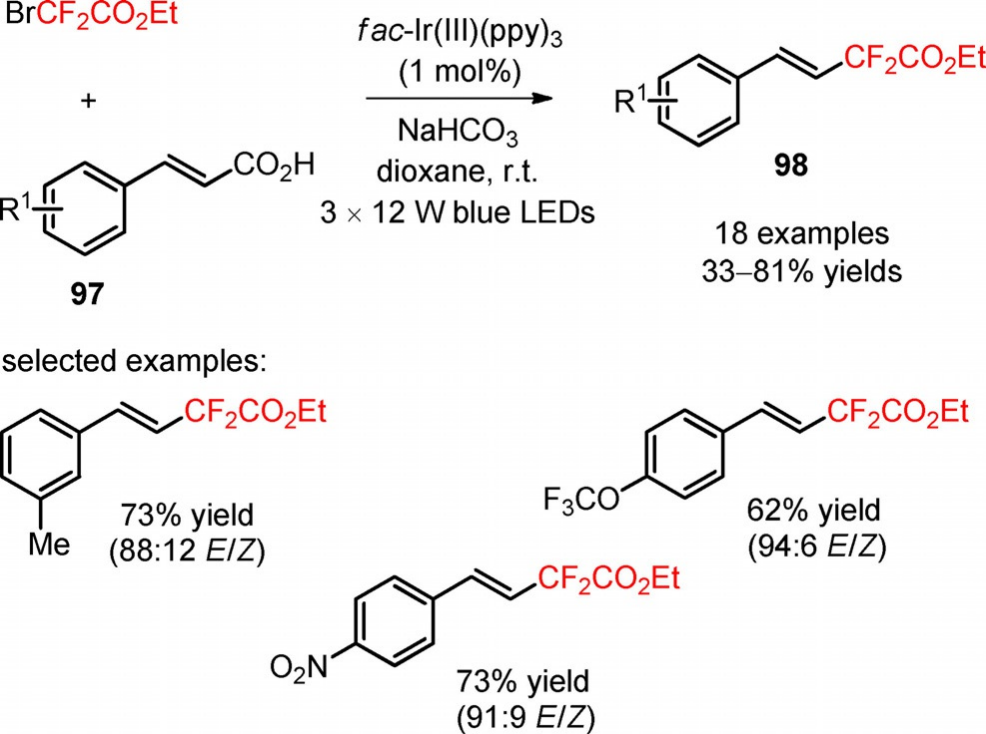
Visible light‐promoted photocatalytic decarboxylative difluoroalkylation of *meta*‐ and *para*‐substituted α,β‐unsaturated carboxylic acids (**97**).

**Scheme 20:**
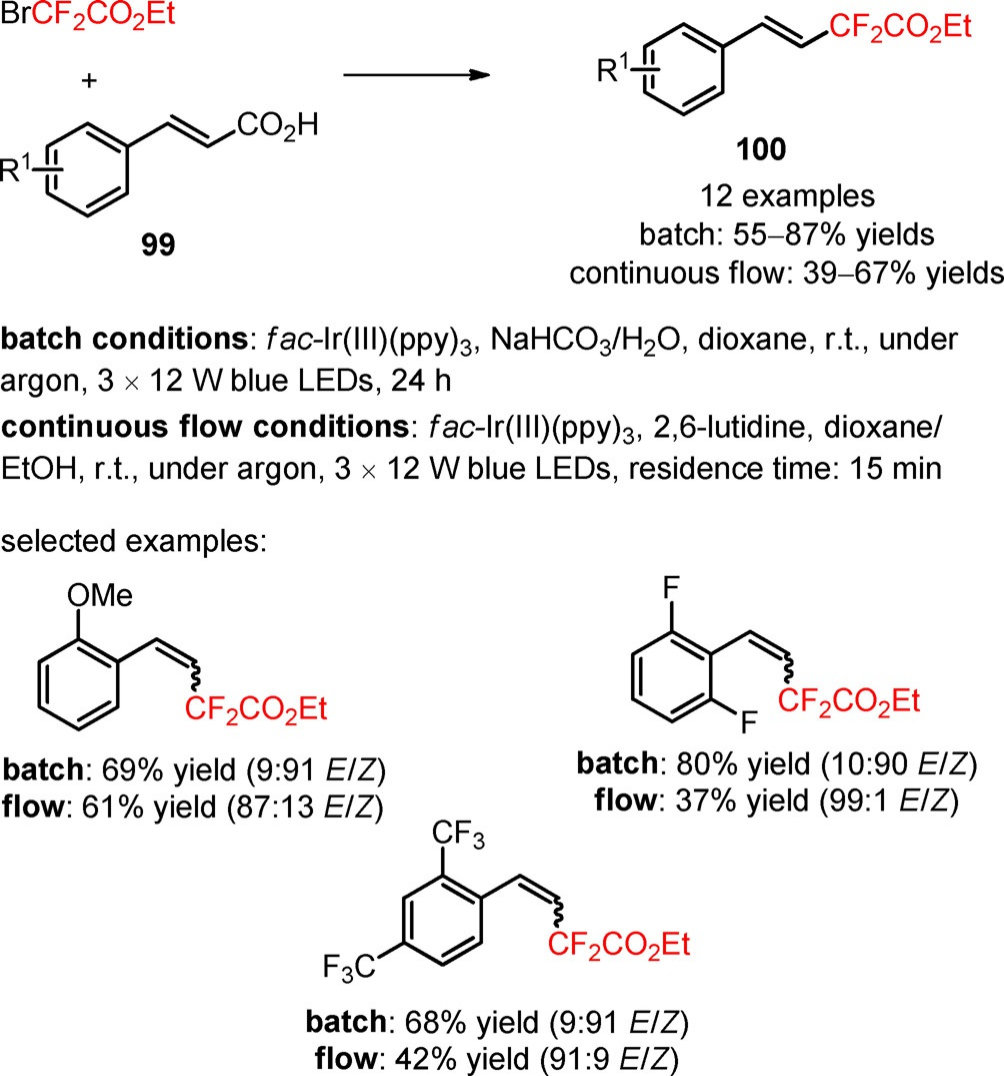
Visible light‐promoted photocatalytic decarboxylative difluoroalkylation of *ortho‐*substituted α,β‐unsaturated carboxylic acids (**99**) under batch and continuous flow conditions.

**Scheme 21:**
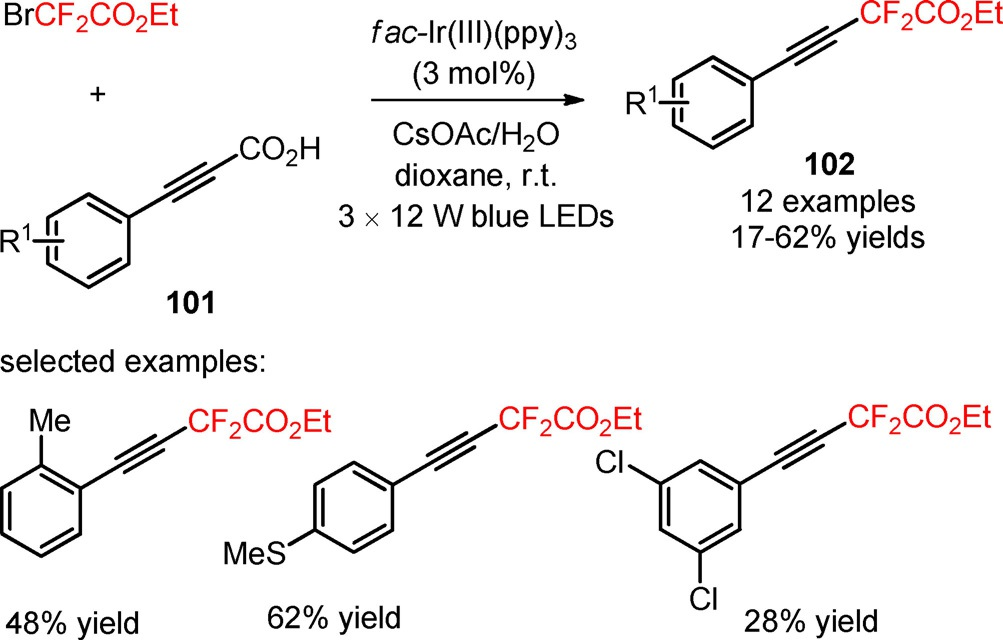
Visible light‐promoted photocatalytic decarboxylative difluoroalkylation of arylpropiolic acids (**101**).

As an alternative to ICF_2_CO_2_Et, Noël and co‐workers employed the reagent BrCF_2_CO_2_Et for the decarboxylative difluoroalkylation of α,β‐unsaturated carboxylic acids (**97**) in the presence of *fac*‐Ir(III)(ppy)_3._ 82 The developed strategy required no higher temperatures, no metal co‐catalysts, or hypervalent iodine reagents to facilitate the decarboxylation process. A spectrum of *meta*‐ and *para*‐substituted α,β‐unsaturated carboxylic acids bearing electron‐neutral, electron‐donating, and electron‐withdrawing substituents on the aromatic ring and heterocyclic substituents (pyridine and thiophene) (**97**) afforded the respective difluoroalkylated styrenes with a good to excellent *E*‐stereoselectivity (**98**, Scheme 19: 18 examples, 33–81% yields). In contrast, the decarboxylative difluoroalkylation of *ortho*‐substituted α,β‐unsaturated carboxylic acids (**99**) provided the corresponding *Z*‐products (**100**) under batch conditions. A switch in the stereoselectivity was observed when the decarboxylative functionalization of the substrates was performed under continuous‐flow conditions (**100**, Scheme 20: 12 examples, batch: 55–87% yields, continuous flow: 39–67% yields). Interestingly, this methodology can be successfully applied to *ortho*‐, *meta*‐, *para*‐substituted arylpropiolic acids (**101**) for the synthesis of difluoroalkylated phenylacetylenes (**102**, Scheme 21: 12 examples, 17–62% yields).

**Scheme 22:**
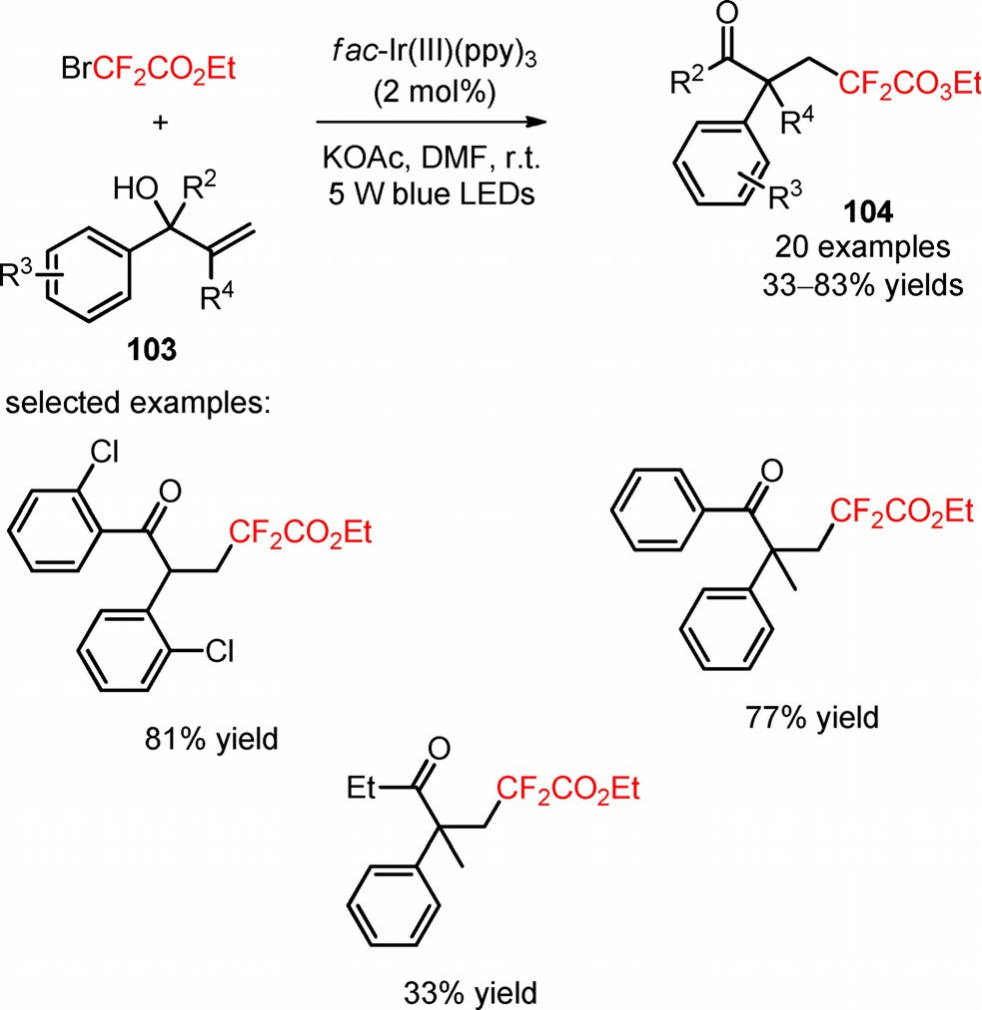
Photoinduced carbo‐difluoroalkylation of α,α‐diarylallylic alcohols (**103**).

The synthesis of carbodifluoroalkylated ketones by visible light‐promoted difunctionalization of allylic alcohols through a sequential difluoroalkylation and functional group migration process has been reported in the literature. Zhu and co‐workers disclosed an efficient methodology for the carbodifluoroalkylation of α,α‐diarylallylic alcohols with electron‐donating and electron‐withdrawing substituents (**103**) attached to the aromatic rings and a subsequent 1,2‐aryl migration process, in the presence of *fac*‐Ir(III)(ppy)_3_, the base KOAc, and BrCF_2_CO_2_Et (**104**, Scheme 22: 20 examples, 33–83% yields).^83^ The difluoroalkylation/functional group migration process was also achievable using bromodifluoroacetamides.

**Scheme 23:**
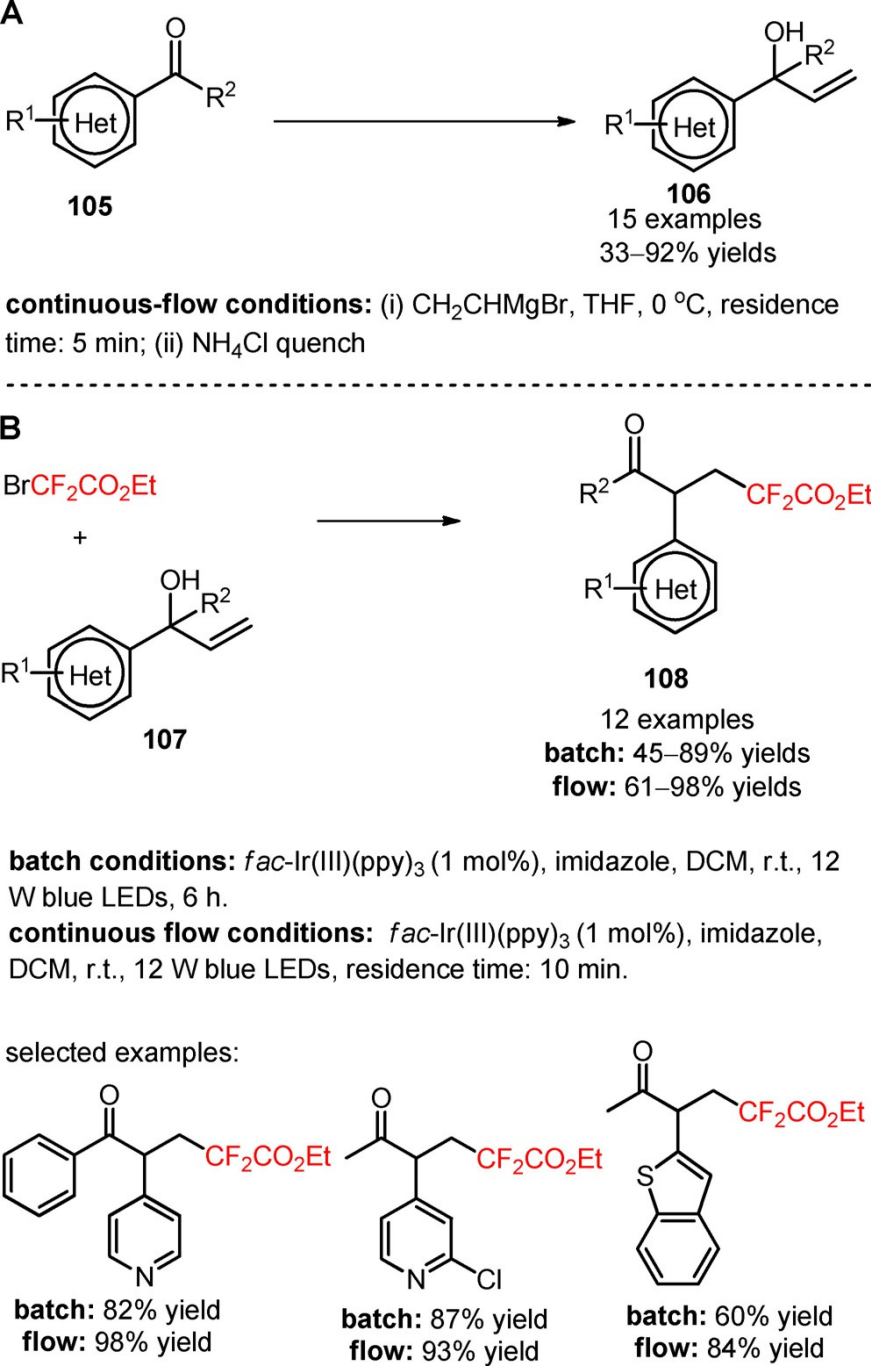
Photocatalytic difluoroalkylation‐induced 1,2‐heteroaryl migration of allylic alcohols (**106**).

Recently, Noël's group reported a similar synthetic strategy for the difluoroalkylation of heteroaryl‐containing allylic alcohols (**106**) and concomitant 1,2‐heteroaryl migration with BrCF_2_CO_2_Et.^84^ Heteroaryl‐containing allylic alcohols were synthesized *via* reactions between heteroaryl ketones (**105**) and vinylmagnesium bromide, under continuous‐flow conditions (**106**, Scheme 23A: 15 examples, 33–92% yields). A higher efficiency for the difluoroalkylation/1,2‐heteroaryl migration process was achieved when *fac*‐Ir(III)(ppy)_3_ and imidazole were chosen as photocatalyst and base, respectively. Under the optimized photochemical conditions, the 4‐pyridyl, 3‐pyridyl, 2‐pyridyl, pyrazyl, and benzothiophenyl groups exhibited a migratory aptitude induced by incorporation of CF_2_CO_2_Et groups, affording the final products in good yields under batch conditions (**107**, Scheme 23B: 12 examples, batch: 45–89% yields). A switch to continuous‐flow conditions enabled a reduction of the reaction time with a concomitant increase of the reaction yields (batch: 45–89% yields *vs*. continuous‐flow: 61–98% yields). The radical addition of two CF_2_CO_2_Et groups was observed with benzofuranyl‐ and thiophenyl‐containing substrates, yielding the respective bis‐functionalized derivatives. Other difluoroalkyl precursors including BrCF_2_PO(OEt)_2_ and bromodifluoroacetamide derivatives efficiently promoted the heteroaryl migration. Mechanistic experiments with the radical scavenger 2,6‐di‐*tert*‐butyl‐4‐methylphenol (BHT) corroborated the involvement of a radical‐mediated difluoroalkylation.

**Scheme 24:**
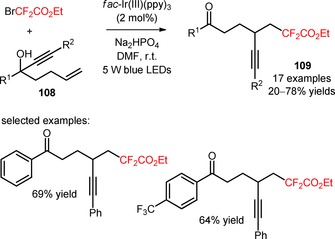
Photoinduced difluoroalkylation of unactivated alkenes (**108**) in the presence of *fac*‐Ir(III)(ppy)_3_.

Alkynyl‐substituted difluoroalkyl ketones were achieved by photoinduced difluoroalkylation of unactivated alkenes (**108**) with BrCF_2_CO_2_Et and subsequent migration of the alkynyl groups (**109**, Scheme 24: 17 examples, 20–78% yields).^85^ A series of aromatic alkynyl motifs bearing electron‐donating and electron‐withdrawing groups exhibited this migratory aptitude. The developed methodology can be extended to different difluoroalkyl reagents such as bromodifluoroacetamides and 2‐bromo‐2,2‐difluoro‐1‐morpholinoethan‐1‐one.

**Scheme 25:**
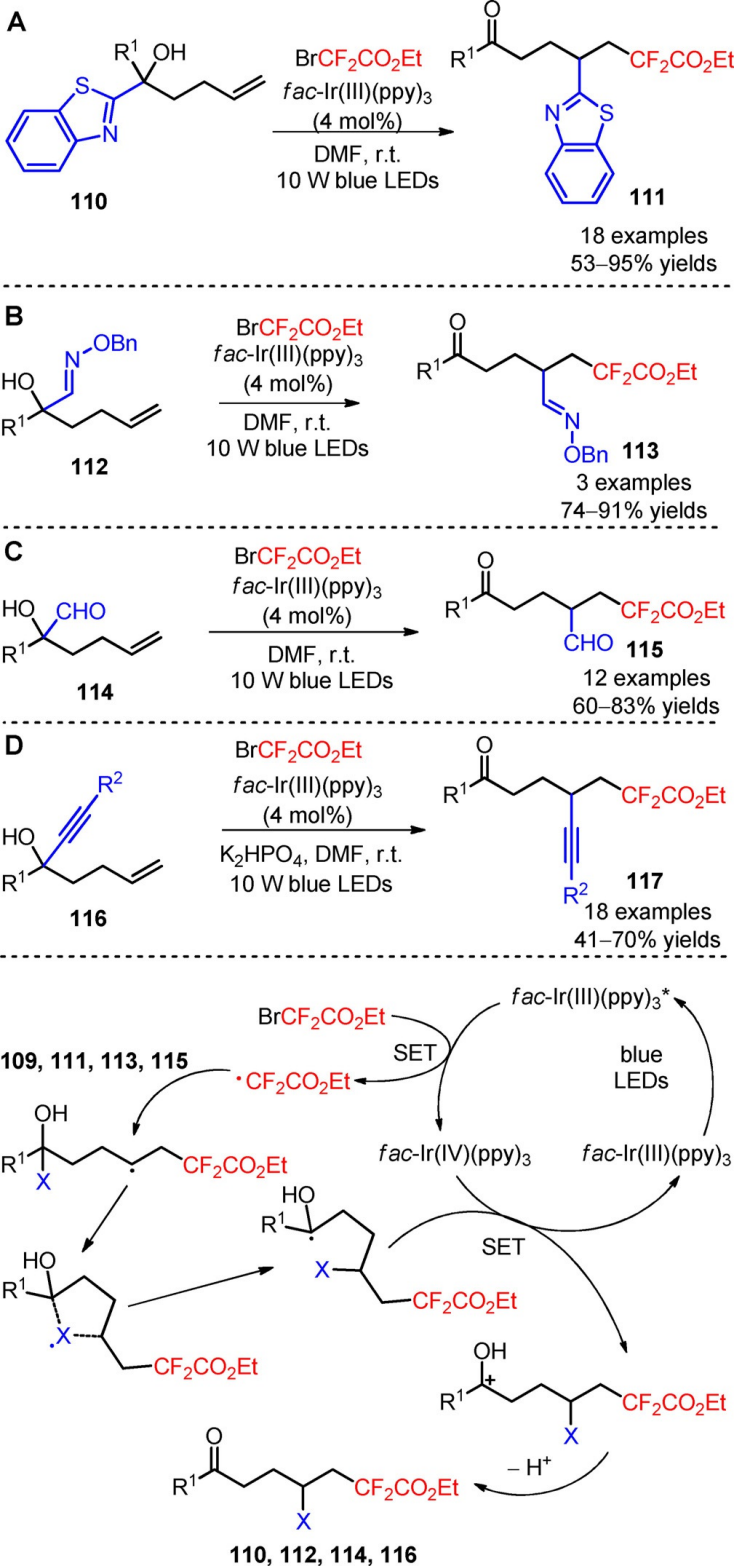
Visible light‐induced heteroaryl‐ (**110**), imino‐ (**112**), formyl‐ (**114**), and alkynyl‐difluoroalkylation of unactivated alkenes (**116**) based on distal functional group migration and the proposed mechanism.

The strategy of distal functional group migration was also implemented by Zhu and collaborators for the carbodifluoroalkylation of unactivated alkenes (**110**, **112**, **114**, **116**) in combination with visible light photocatalysis.^86^ For the carbodifluoroalkylation process, intramolecular migration was observed for products bearing a series of functional groups including heteroaryl (**111**, Scheme 25A: 18 examples, 53–95% yields), imino (**113**, Scheme 25B: 3 examples, 74–91% yields), formyl (**115**, Scheme 25C: 12 examples, 60–83% yields), and alkynyl groups (**117**, Scheme 25D: 18 examples, 41–70% yields), in the presence of *fac*‐Ir(III)(ppy)_3_ and BrCF_2_CO_2_Et. The authors suggested a mechanism involving electrophilic addition of CF_2_CO_2_Et radicals to the alkene moiety and subsequent cyclization with the radical acceptor groups (heteroaryl, imino, formyl, and alkynyl groups). Ring‐opening homolysis followed by oxidation *via fac*‐Ir(IV)(ppy)_3_ and base‐mediated deprotonation gave the respective difluoroalkylated ketones (**111**, **113**, **115**, **117**).

**Scheme 26:**
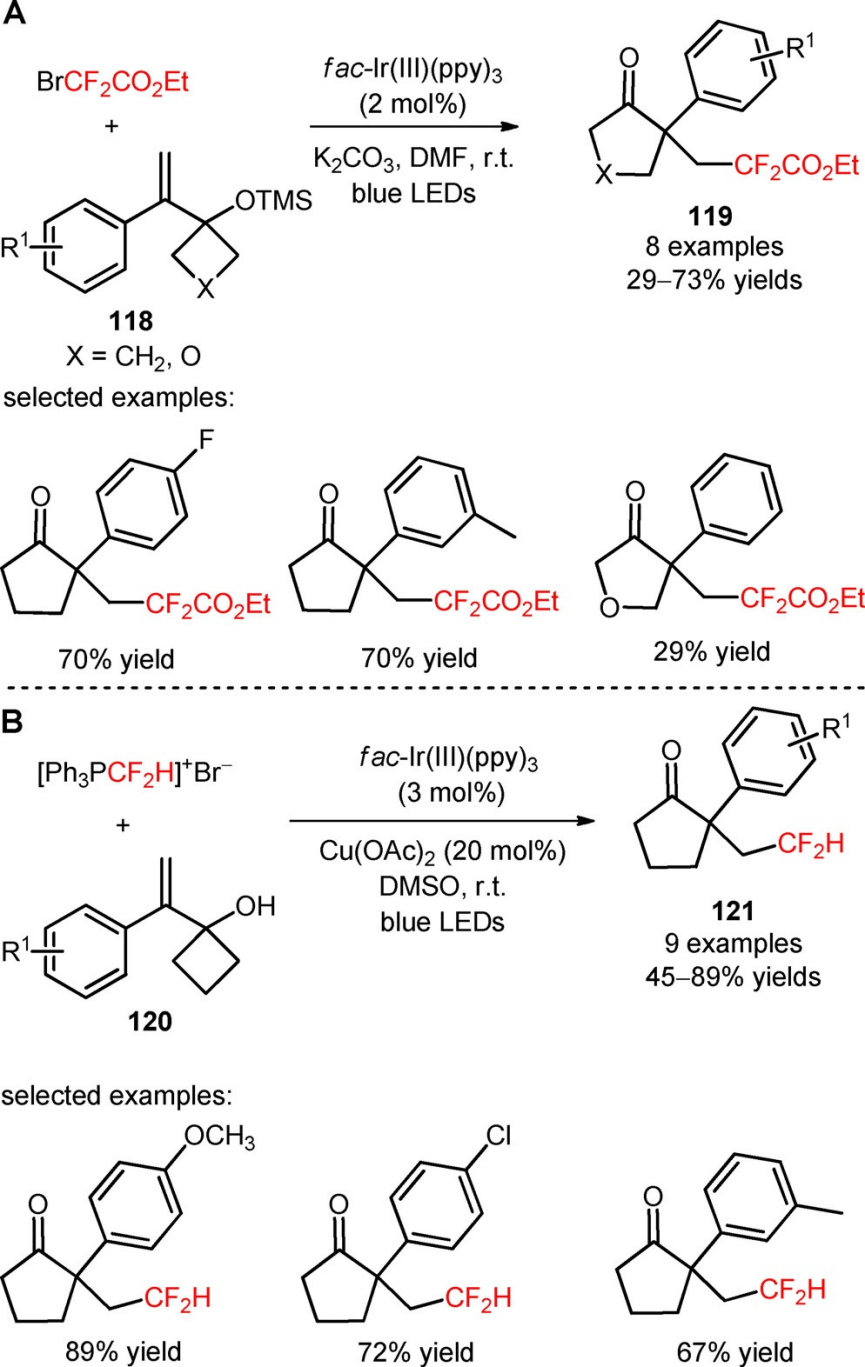
Visible light‐induced photocatalytic difluoroalkylation/1,2‐carbon migration of 1‐(1‐arylvinyl)cyclobutanol derivatives (**118**, **120**) with BrCF_2_CO_2_Et (**A**) and [Ph_3_PCF_2_H]^+^Br^−^ (**B**).

Visible light‐mediated difluoroalkylation of 1‐(1‐arylvinyl)cyclobutanol derivatives (**118**, **120**) and ring expansion *via* 1,2 carbon migration was described by Kim and collaborators using the difluoroalkyl precursors BrCF_2_CO_2_Et^87^ and [Ph_3_PCF_2_H]^+^Br^−^.^88^ A wide range of 1‐(1‐arylvinyl)cyclobutanols bearing electron‐donating, electron‐neutral, and electron‐withdrawing groups furnished the difluoroalkyl‐substituted cyclic ketones with moderate to good yields (**119**, Scheme 26A: 8 examples, 29–73% yields; **121**, Scheme 26B: 9 examples, 45–89% yields).

**Scheme 27:**
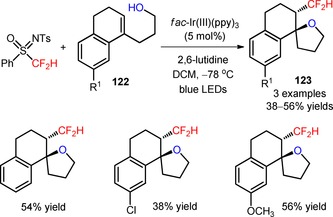
Oxy‐difluoromethylation of aryl‐fused cycloalkenylalkanols (**122**) with *N*‐tosyl‐*S*‐difluoromethyl‐*S*‐phenylsulfoximine under visible light photoredox conditions.

The difluoromethyl precursor *N*‐tosyl‐*S*‐difluoromethyl‐*S*‐phenylsulfoximine was effectively implemented in the diastereoselective synthesis of *anti*‐difluoromethyl‐substituted spiroethers through visible light‐mediated oxy‐difluoromethylation of aryl‐fused cycloalkenyl alcohol derivatives (**122**) in the presence of *fac*‐Ir(III)(ppy)_3_ (**123**, Scheme 27: 3 examples, 38–56% yields).^89^

### 1.4 Difluoroalkylation of *sp* ^2^ Carbon Atoms in Unsaturated Amides, Hydrazones, and Allylamines

**Scheme 28:**
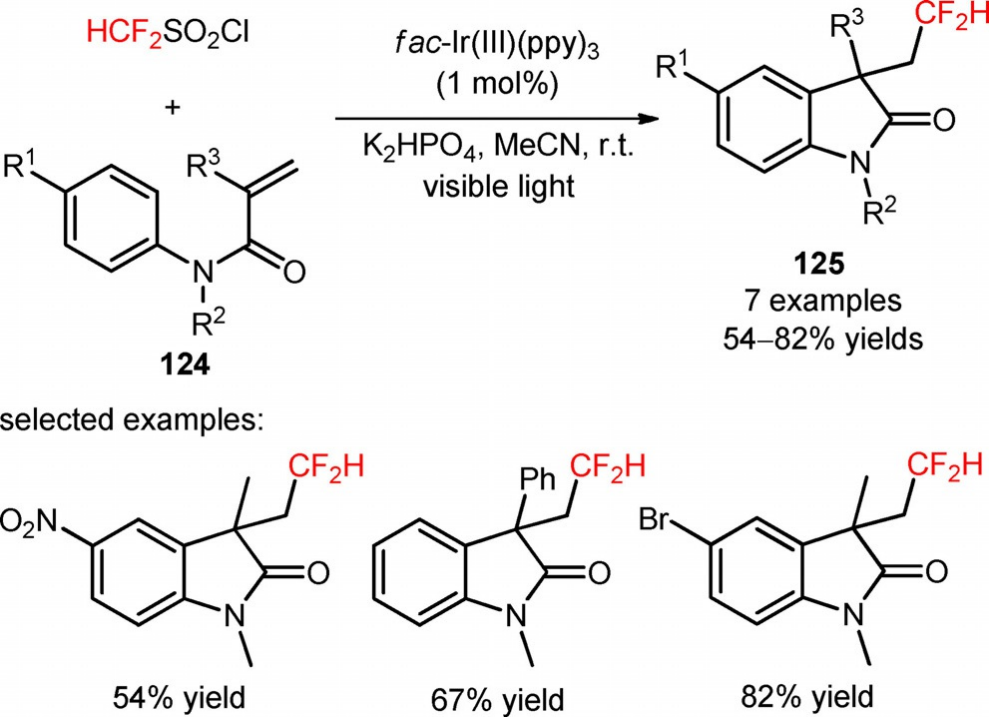
Visible light‐mediated tandem difluoromethylation/cyclization of *N*‐arylacrylamides (**124**) with HCF_2_SO_2_Cl.

The radical difluoroalkylation of a series of unsaturated amides has been employed as an efficient strategy of producing synthetic precursors to access more complex and functionalized heterocyclic derivatives. In 2014, Dolbier and collaborators disclosed a methodology for tandem difluoromethylation of *N*‐arylacrylamides (**124**) with HCF_2_SO_2_Cl and consecutive cyclization, under irradiation with visible light.^90^ The authors found that the *fac*‐Ir(III)(ppy)_3_ was the most effective catalyst for the reduction of HCF_2_SO_2_Cl and generation of CF_2_H radicals under mild conditions. The introduction of electron‐donating and electron‐withdrawing groups in the aromatic ring of *N‐*arylacrylamides (**124**) was well tolerated with the desired organic transformation affording the respective difluoromethylated 3,3‐disubstituted 2‐oxindoles in moderate to good yields (**125**, Scheme 28: 7 examples, 54–82% yields).

**Scheme 29:**
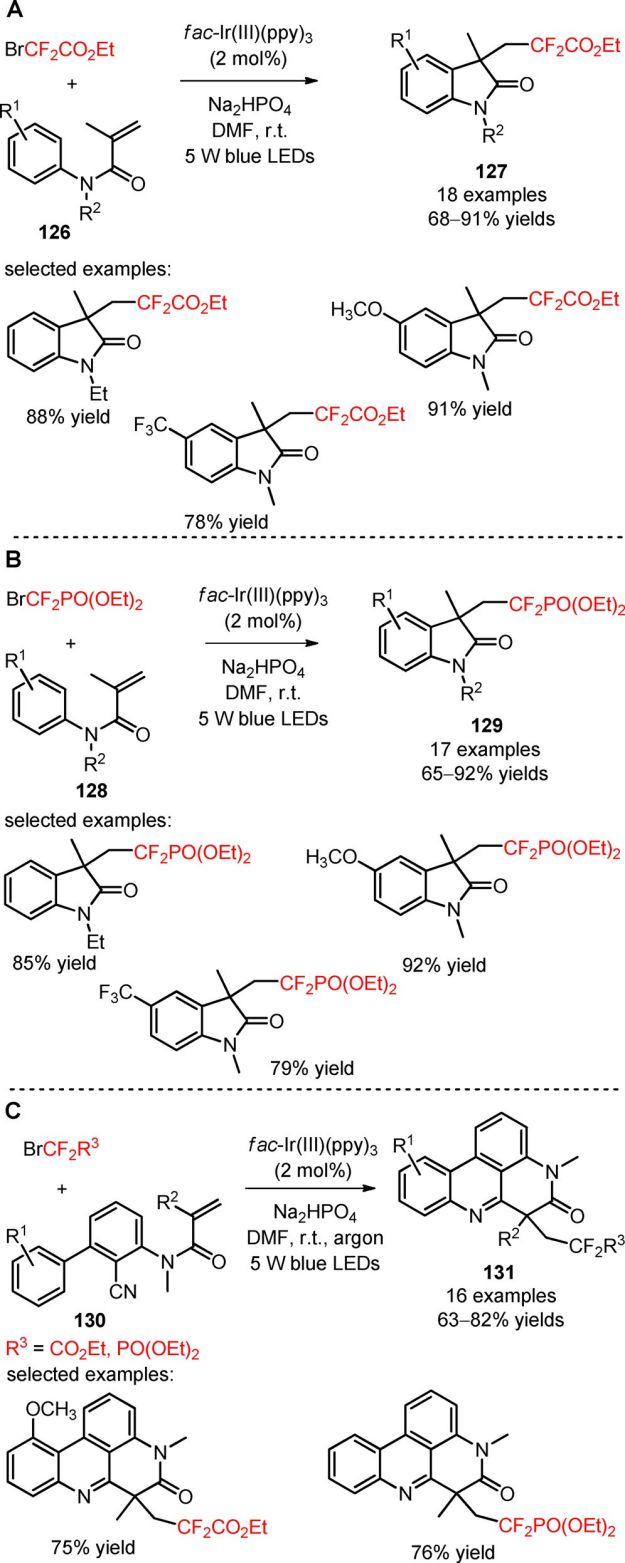
Visible light‐driven difluoroalkylation of *N*‐arylacrylamides with BrCF_2_CO_2_Et (**A**), BrCF_2_PO(OEt)_2_ (**B**) and both reagents (**C**).

Other difluoroalkylating reagents, including BrCF_2_CO_2_Et and BrCF_2_PO(OEt)_2_, have been implemented in the preparation of difluoroalkylated oxindoles from *N‐*arylacrylamides (**126**, **128**), in the presence of *fac*‐Ir(III)(ppy)_3_ and the base Na_2_HPO_4_ (**127**, Scheme 29A: 18 examples, 68–91% yields; **129**, Scheme 29B: 17 examples, 65–92% yields).^91^,^92^ In 2017, Sun and co‐workers developed a methodology for the photoinduced difluoroalkylation with BrCF_2_CO_2_Et and BrCF_2_PO(OEt)_2_ of *N*‐arylacrylamides (**130**) and consecutive intramolecular radical addition to the cyano groups and homolytic aromatic substitution.^93^ A variety of *N*‐arylacrylamides bearing electron‐donating and electron‐withdrawing groups on the aromatic ring furnished the difluoroalkylated phenanthridines (**131**, Scheme 29C: 16 examples, 63–82% yields). Alternative approaches for the synthesis of functionalized phenanthridines from biphenyl isocyanides will be discussed in Section 2.5.

**Scheme 30:**
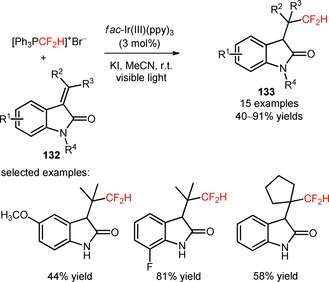
Photoinduced difluoromethylation of oxindole‐derived alkenes (**132**).

An alternative methodology for the synthesis of difluoromethylated oxindoles was developed by Qing and co‐workers through the photoinduced hydro‐difluoromethylation of oxindole‐derived alkenes (**132**) using the difluoromethylating reagent [Ph_3_PCF_2_H]^+^Br^−^.^94^ In the presence of *fac*‐Ir(III)(ppy)_3_, a wide range of oxindole‐derived alkenes bearing electron‐donating and electron‐withdrawing substituents on their aromatic ring and polysubstituted oxindole derived‐alkenes afforded the hydro‐difluoromethylated derivatives in moderate to high yields (**133**, Scheme 30: 15 examples, 40–91% yields).

**Scheme 31:**
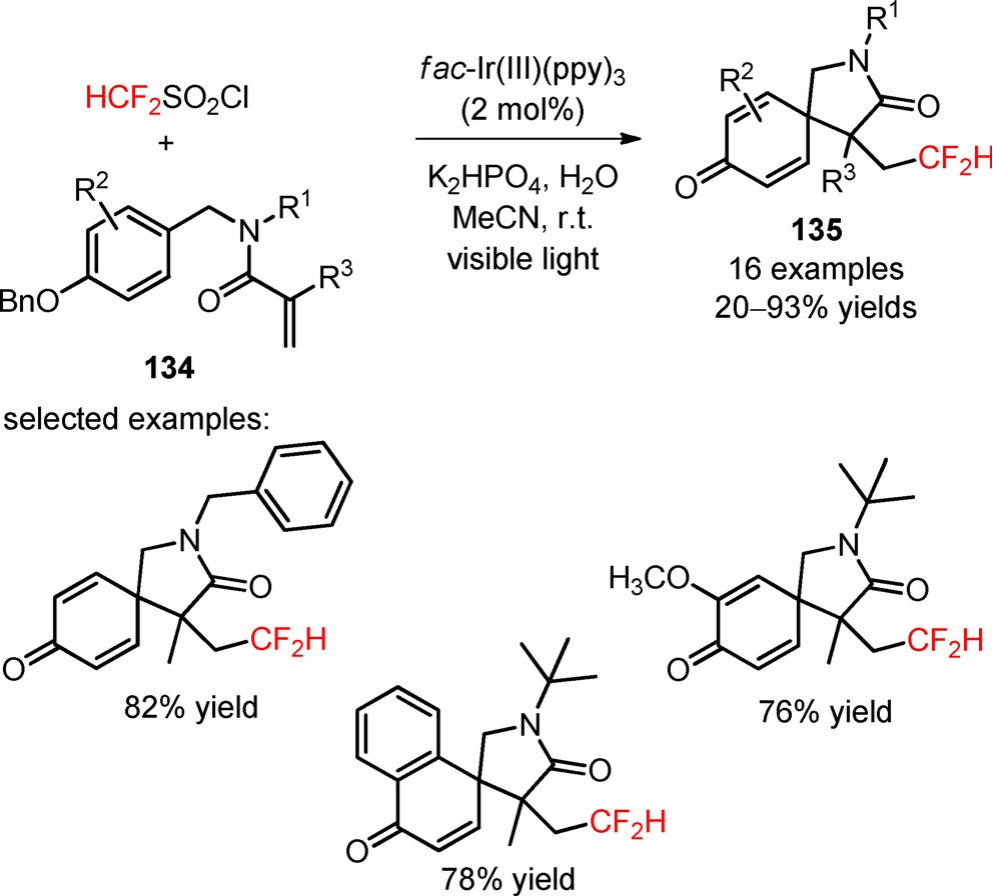
Photoinduced difluoromethylation/5‐*exo* radical cyclization of *N*‐benzylacrylamides (**134**).

Difluoromethylated 2‐azaspiro[4.5]deca‐6,9‐diene‐3,8‐diones (**135**) were prepared by Dolbier and collaborators *via* the photoinduced difluoromethylation of *N*‐benzylacrylamides (**134**) with HCF_2_SO_2_Cl and subsequent 5‐*exo*‐cyclization.^95^ Apart from the relevance of *fac*‐Ir(III)(ppy)_3_ and the base K_2_HPO_4_ in the difluoromethylation/5‐*exo*‐cyclization process, the addition of water to the reaction system influenced significantly its efficiency. A wide scope of *N*‐benzylacrylamides containing *N*‐substituents such as cyclohexyl, isopropyl, *n*‐butyl, and *tert*‐butyl, and electron‐rich and electron‐deficient aromatic substituents furnished the desired products (**135**, Scheme 31: 16 examples, 20–93% yields). The authors found that the steric properties of the *N*‐substituents may influence the efficiency of the spirocyclization process. This synthetic approach can be extended to other fluoroalkyl radical sources, in particular BrCF_2_CO_2_Et.

**Scheme 32:**
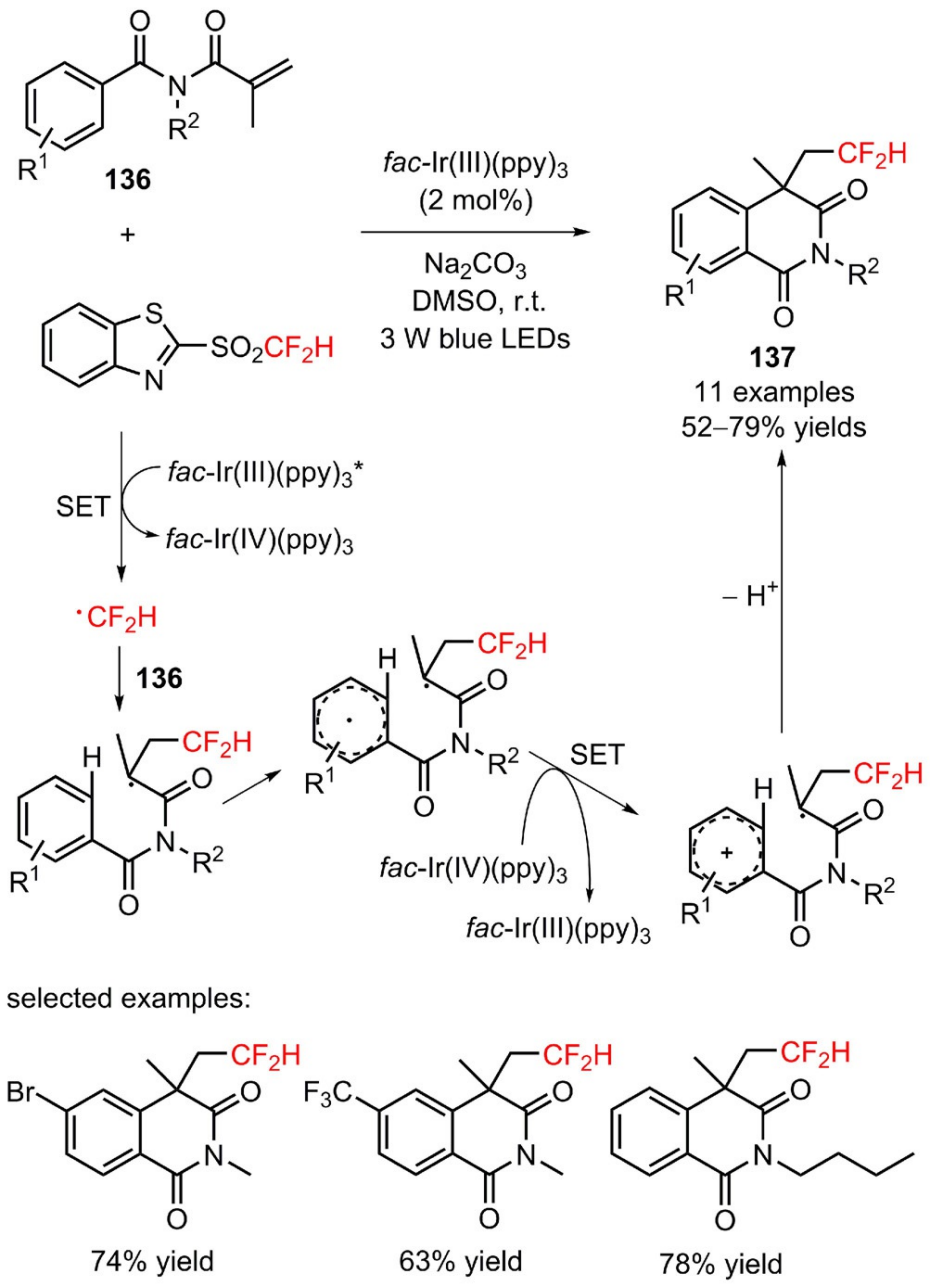
Visible light‐driven difluoromethylation of *N*‐methacryloylbenzamides (**136**) with 2‐[(difluoromethyl)sulfonyl]benzo[*d*]thiazole (2‐BTSO_2_CF_2_H).

Later, Wang's group used 2‐[(difluoromethyl)sulfonyl]benzo[*d*]thiazole (2‐BTSO_2_CF_2_H, CAS number: 186204‐66‐0) for the radical difluoromethylation of *N*‐methacryloylbenzamides (**136**) and consecutive intramolecular cyclization, under visible light photoredox conditions.^96^ A palette of difluoromethylated isoquinoline‐1,3(2*H*,4*H*)‐diones bearing *N*‐alkyl substituents, electron‐rich, and electron‐deficient aromatic substituents on the benzamide moiety was successfully obtained in moderate to good yields (**137**, Scheme 32: 11 examples, 52–79% yields). The authors suggested a mechanism involving oxidative quenching of *fac*‐Ir(III)(ppy)_3_* and reduction of 2‐BTSO_2_CF_2_H to CF_2_H radicals for the preparation of the difluoromethylated products (**137**).

**Scheme 33:**
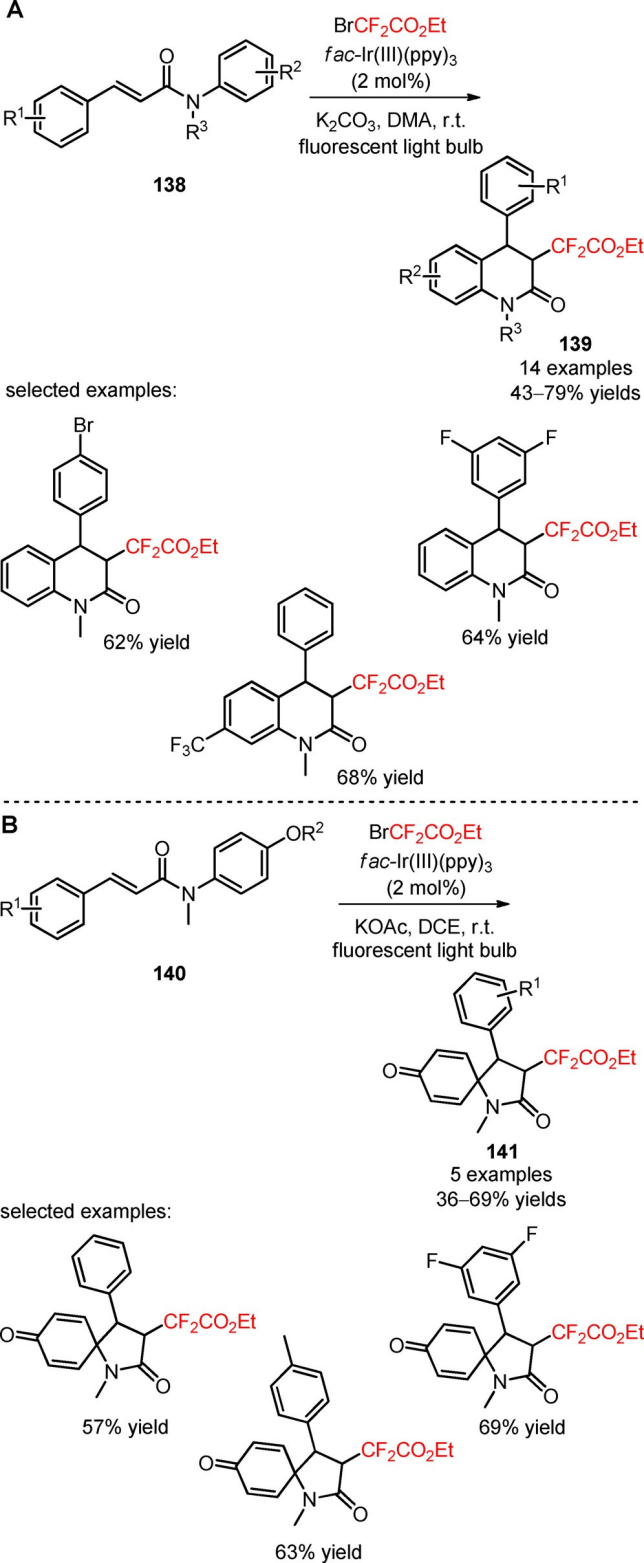
Photoinduced difluoroalkylation of *N*‐phenylcinnamamides (**138**, **140**) for the regioselective synthesis of difluoroalkylated quinoline‐2‐ones (6‐*endo* cyclization) (**139**) and 1‐azaspiro[4.5]decanes (5‐*exo* cyclization/dearomatization) (**141**).

*N*‐Phenylcinnamamides were applied as substrates for the visible light‐catalyzed difluoroalkylation using the reagent BrCF_2_CO_2_Et.^97^ In the presence of *fac*‐Ir(III)(ppy)_3_, a broad scope of *N*‐phenylcinnamamides possessing bulky groups at the *N*‐position and electron‐withdrawing groups attached to the aromatic rings (**138**) proved to be compatible substrates for the synthesis of difluoroalkylated quinoline‐2‐ones after intramolecular 6‐*endo* cyclization (**139**, Scheme 33A: 14 examples, 43–79% yields). A different pattern of cyclization was found when methoxy or hydroxy groups were attached to the aromatic amide moiety in the *para* position of the aromatic rings (**140**). The unexpected 5‐*exo* cyclization/dearomatization process afforded the respective difluoroalkylated spiro[4.5]decanes under the defined reaction conditions (**141**, Scheme 33B: 5 examples, 36–69%).

**Scheme 34:**
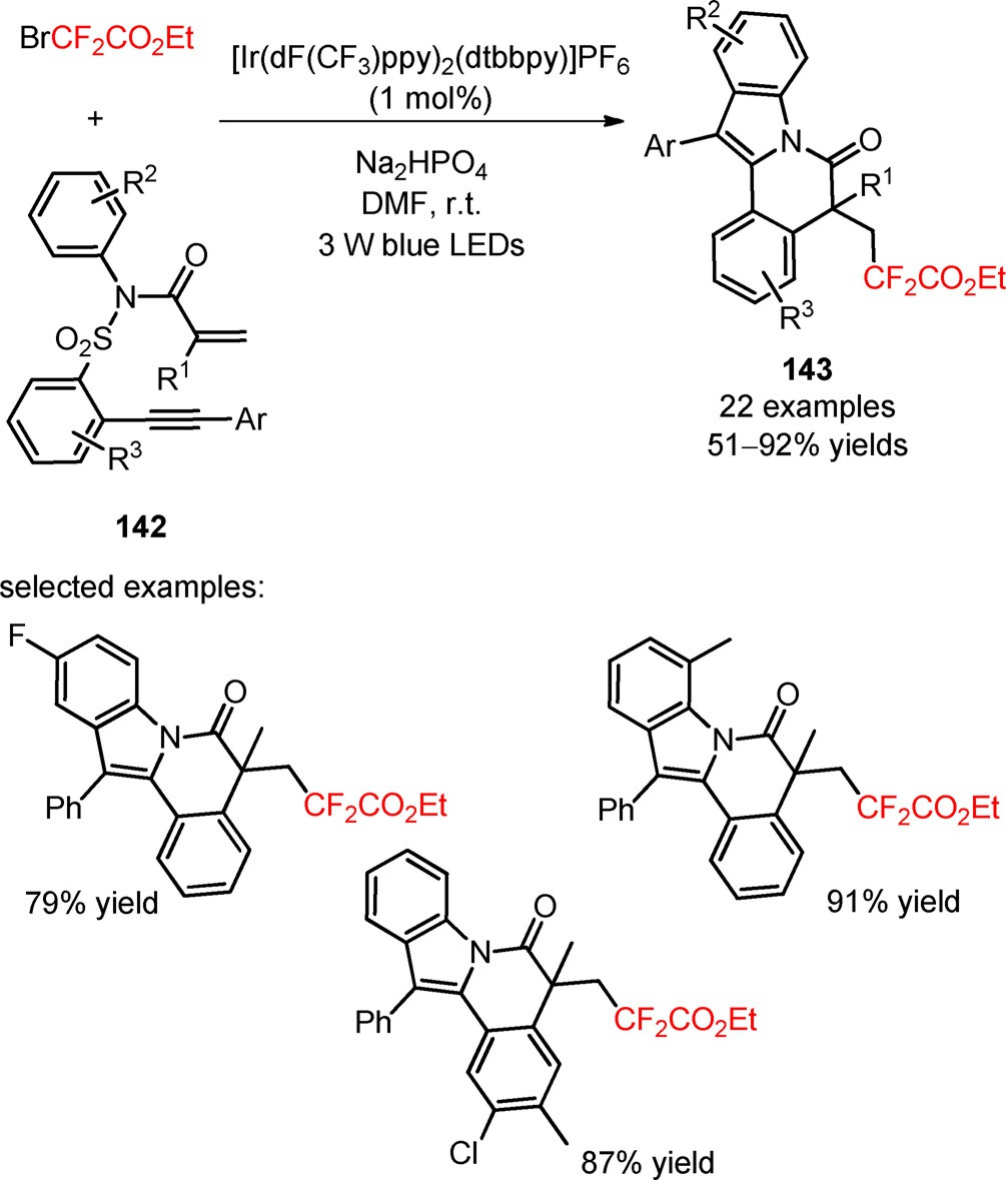
Visible light‐mediated radical difluoroalkylation/cyclization of 1,8‐enynes (**142**).

Difluoroalkylated tetracycles embedded with indole and dihydroisoquinolinone scaffolds (**143**) were effectively constructed by the photoinduced difluoroalkylation/cyclization of 1,8‐enynes (**142**) with the reagent BrCF_2_CO_2_Et, in the presence of [Ir(dF(CF_3_)ppy)_2_(dtbbpy)]PF_6._ 98 Under irradiation with 3 W blue LEDs, the combination of the selected photocatalyst with the base Na_2_HPO_4_ provided the best reaction conditions for the respective difluoroalkylation process (**143**, Scheme 34: 22 examples, 51–92% yields). The difluoroalkyl group of the corresponding products can undergo postfunctionalization steps and be converted into difluoroalkylated alcohol, amide or carboxylic acid derivatives.

**Scheme 35:**
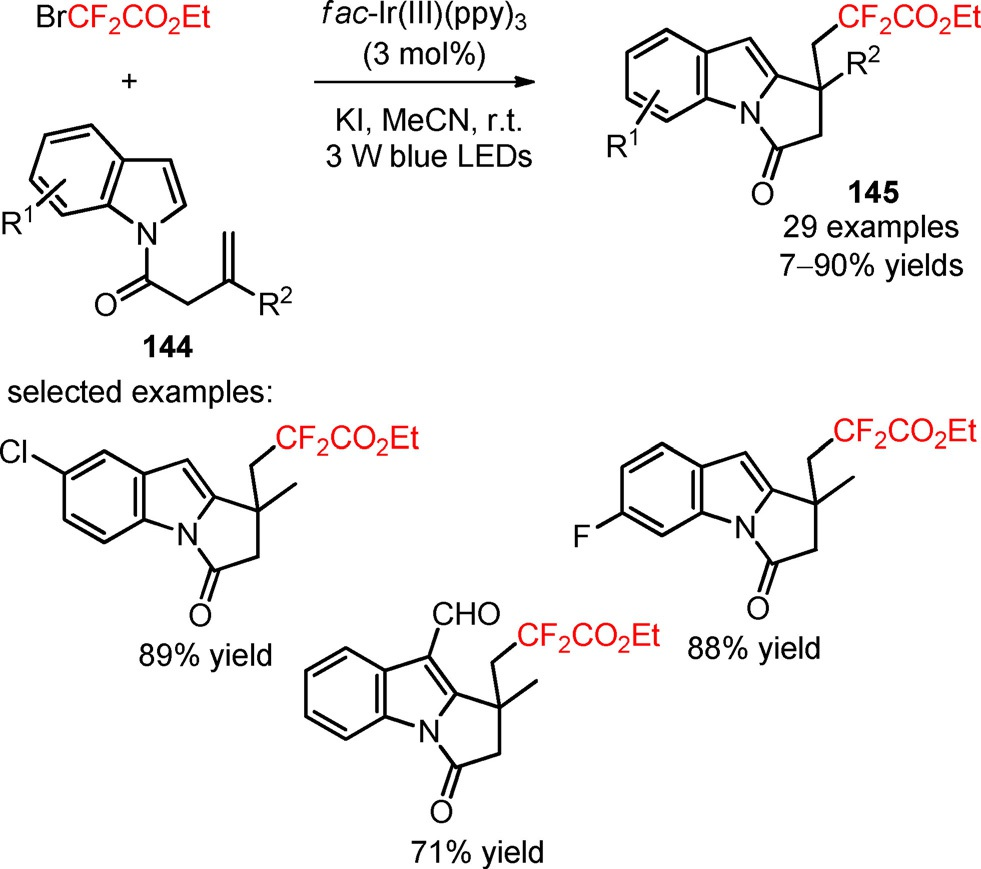
Photoinduced difluoroalkylation/cyclization reaction of *N*‐(but‐2‐enoyl)indoles (**144**).

Recently, Li and co‐workers applied the difluoroalkylating reagent BrCF_2_CO_2_Et for the preparation of difluoroalkylated pyrrolo[1,2‐*a*]indoles (**145**) from *N*‐(but‐2‐enoyl)indoles (**144**), under irradiation with 3 W blue LEDs.^99^ The introduction of electron‐donating and electron‐withdrawing groups on the aromatic ring and of structurally distinct functional groups on the heteroarene ring of the indole moiety (e.g., cyano, ester, formyl, phenyl, and methyl substituents) was compatible with the desired organic transformation (**145**, Scheme 35: 29 examples, 7–90% yields). Remarkably, difluoroalkylation with alternative reagents derived from difluoroalkyl bromides, including bromoacetates, bromodifluoromethyl ketones, bromodifluoroacetamides, and BrCF_2_PO(OEt)_2_ proceeded smoothly with the described methodology.

**Scheme 36:**
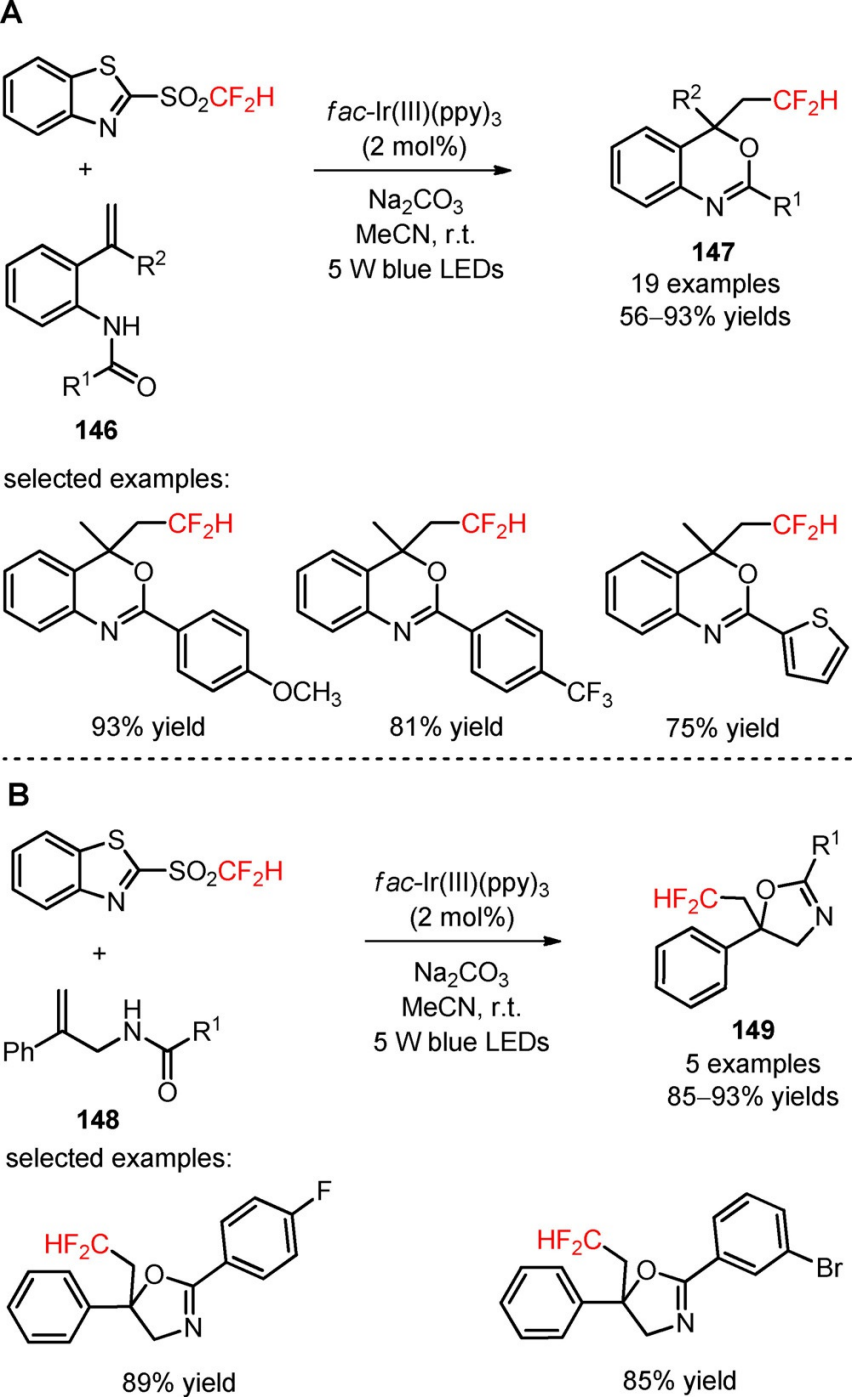
Visible light‐mediated radical oxy‐difluoromethylation of benzamides (**146**) and *N*‐allylamides (**148**) and with 2‐BTSO_2_CF_2_H.

An efficient methodology involving the visible light‐induced incorporation of CF_2_H groups in benzamides (**146**) with 2‐BTSO_2_CF_2_H and concomitant cyclization to a benzoxazine ring has been reported.^100^ A variety of benzamides possessing electron‐neutral, electron‐donating, and electron‐withdrawing groups onto the aromatic rings, heterocyclic, and aliphatic groups (**146**) was efficiently converted into the difluoromethylated benzoxazines (**147**, Scheme 36A: 19 examples, 56–93% yields). Mechanistic experiments with radical scavengers suggested the occurrence of radical‐mediated difluoromethylation process and reduction of 2‐BTSO_2_CF_2_H to CF_2_H radicals *via* oxidative quenching of *fac*‐Ir(III)(ppy)_3_*. This protocol was expanded to the difluoromethylation of *N*‐allylamides (**148**) in excellent yields (**149**, Scheme 36B: 5 examples, 85–93% yields).

**Scheme 37:**
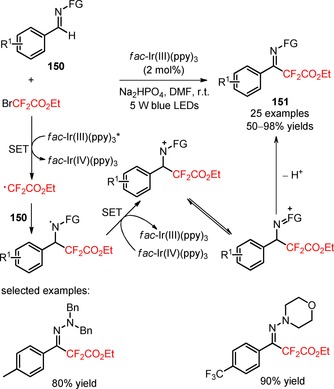
Visible light‐induced difluoroalkylation of aldehyde‐derived hydrazones (**150**) with BrCF_2_CO_2_Et and the proposed mechanism *via* an aminyl radical/polar pathway.

Radical difluoroalkylation of the *sp* ^2^‐hybridized carbon atom of C=N bonds in aldehyde‐derived hydrazones (**150**) with BrCF_2_CO_2_Et was reported by Zhu and co‐workers.^101^ The selection of the photocatalyst *fac*‐Ir(III)(ppy)_3_, the base Na_2_HPO_4_, and the use of 5 W LEDs as light source resulted in an enhanced efficiency of the difluoroalkylation process (Scheme 37). The introduction of an *N*,*N*‐dialkyl structural motif was critical for the reactivity of *N*‐substituted aldehyde‐derived hydrazones. Interestingly, a large diversity of *N,N*‐dialkyl aldehyde‐derived hydrazones bearing electron‐rich and electron‐deficient aromatic groups, heteroaryl, and aliphatic groups (**150**) furnished the difluoroalkylated hydrazones with moderate to excellent yields (**151**, 25 examples, 50–98% yields). Other difluoroalkyl motifs, including bromodifluoroacetamides and phenylalanine‐derived bromodifluoroamide, were also compatible with the developed difluoroalkylation procedure. Two possible reaction pathways were proposed for the formation of difluoroalkyl‐containing hydrazones (**151**): an aminyl radical/polar process and a carbon radical/polar process. Computational calculation of Gibbs free‐energy profiles for both reaction pathways excluded the occurrence of a carbon radical/polar process. Therefore, the authors proposed a mechanism involving the addition of CF_2_CO_2_Et radicals to the C=N bond of the substrates (**150**) and generation of an aminyl radical intermediate. Concurrently, the aminyl radical was oxidized *via fac*‐Ir(IV)(ppy)_3_ to an aminyl cation (aminyl radical/polar cross‐over step). Further tautomerization and deprotonation then furnished the corresponding difluoroalkylated products (**151**).

**Scheme 38:**
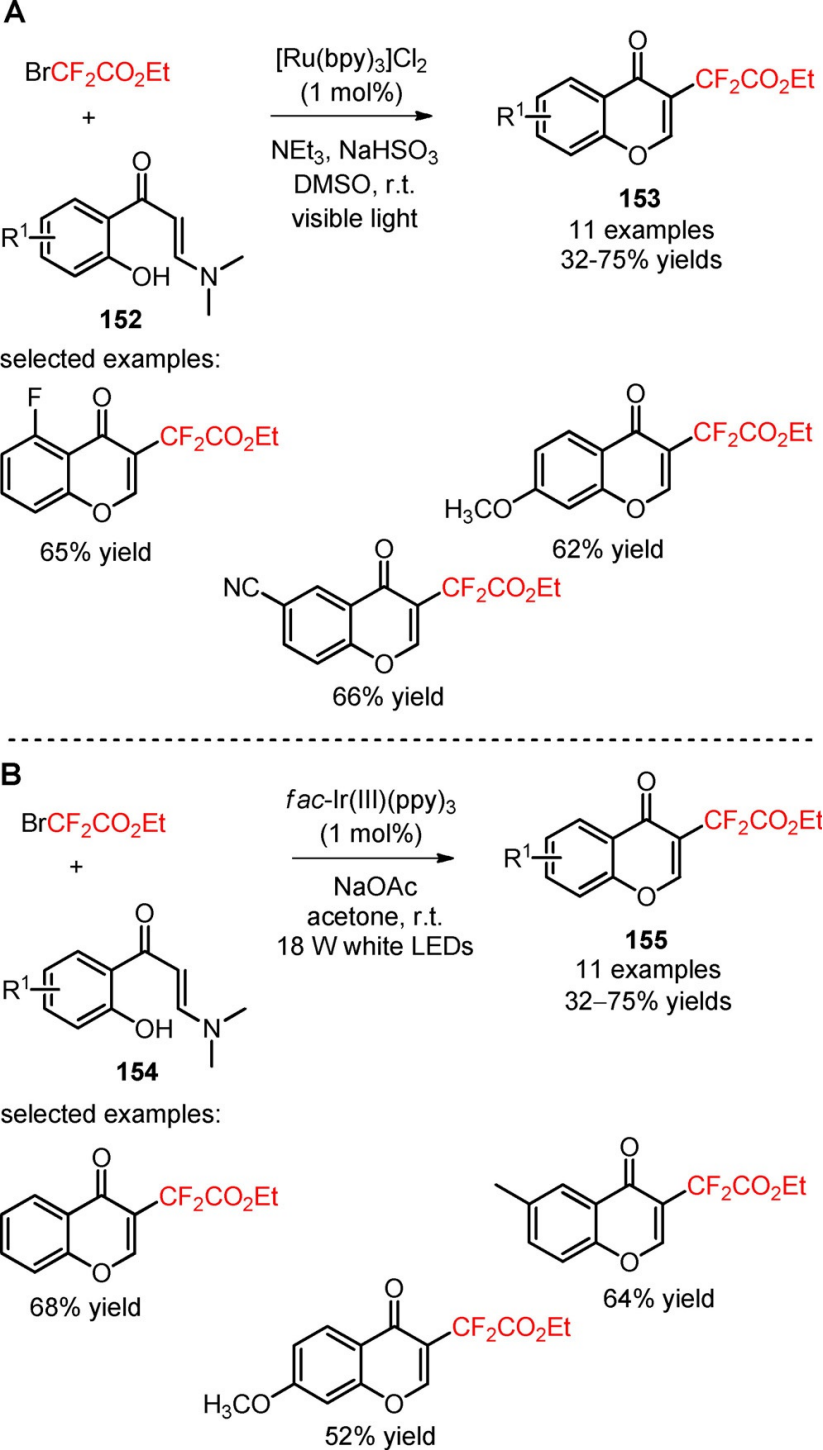
Visible light‐induced difluoroalkylation of *ortho*‐hydroxyaryl enaminones (**152**, **154**) in the presence of [Ru(bpy)_3_]Cl_2_ (**A**) and *fac*‐Ir(III)(ppy)_3_ (**B**).

Apart from unsaturated amides and hydrazones, the difluoroalkylation of allylamine derivatives such as *ortho*‐hydroxyaryl enaminones has also been regarded as a promising approach to access more complex heterocyclic scaffolds of biological relevance. In 2017, two independent works reported by the Zhang^102^ and Yang^103^ groups have described the synthesis of functionalized chromones by the visible light‐mediated difluoroalkylation of *ortho*‐hydroxyaryl enaminones bearing electron‐donating and electron‐withdrawing groups (**152**, **154**) using the reagent BrCF_2_CO_2_Et (**153**, Scheme 38A: 11 examples, 32–75% yields; **155**, Scheme 38B: 11 examples, 32–75% yields).

**Scheme 39:**
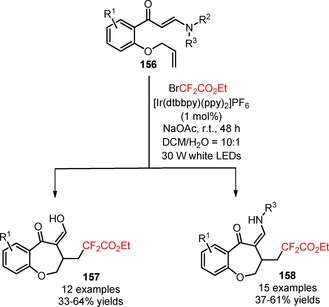
Photoinduced difluoroalkylation of *N,N*‐disubstituted and *N*‐monosubstituted (*E*)‐1‐[2‐(allyloxy)phenyl]‐3‐(substituted amino)prop‐2‐en‐1‐ones (**156**).

Difluoroalkylated benzoxepines were prepared by the introduction of CF_2_CO_2_Et groups into (*E*)‐1‐[2‐(allyloxy)phenyl]‐3‐(substituted amino)prop‐2‐en‐1‐ones (**156**) under visible light photoredox conditions.^104^ Yang's group envisioned that the design of a range of substrates bearing an enaminone moiety and an olefin functionality could trigger an efficient installation of difluoroalkyl moieties and simultaneous intramolecular annulation to afford seven‐membered rings. The combined use of the photocatalyst [Ir(dtbbpy)(ppy)_2_]PF_6_ with BrCF_2_CO_2_Et, the base NaOAc, and the solvent mixture DCM/H_2_O (10:1) proved to be the optimal conditions for the difluoroalkyl radical‐triggered annulation process (Scheme 39). Unexpectedly, the authors found that *N*‐disubstituted enaminones could be hydrolyzed to the corresponding benzoxepines with a 1,3‐dicarbonyl moiety. Substitution at both *meta*‐ and *para*‐positions of the aromatic ring of *N*‐disubstituted enaminones gave the corresponding products in moderate to good yields (**157**, 12 examples, 33–64% yields). In addition, *N*‐monosubstituted substrates bearing structurally diverse acyclic and cyclic groups provided the desired benzoxepine derivatives without the occurrence of a deamination process (**158**, 15 examples, 37–61% yields).

**Scheme 40:**
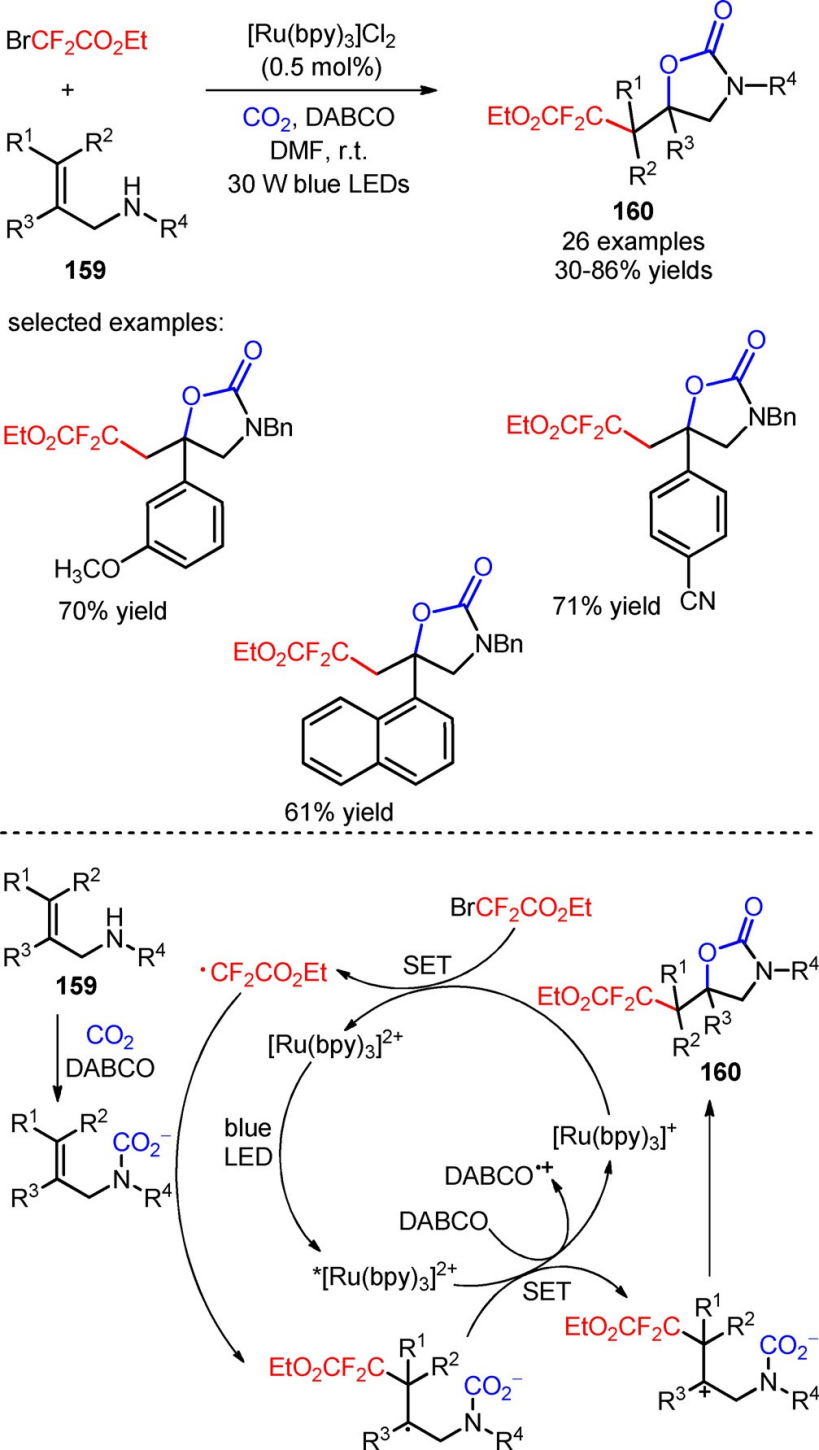
Radical difluoroalkylation/carboxylative cyclization of allylamines (**159**) with CO_2_ and BrCF_2_CO_2_Et *via* visible light photoredox catalysis and the proposed mechanism.

Yu and collaborators developed a novel approach for the utilization of CO_2_ and BrCF_2_CO_2_Et in the difluoroalkylation of allylamines (**159**) and subsequent carboxylative cyclization *via* visible light photoredox catalysis, under atmospheric conditions.^105^ In the presence of [Ru(bpy)_3_]Cl_2_ and the base DABCO, a large variety of difluoroalkylated 2‐oxazolidinones bearing electron‐rich and electron‐poor aromatic groups was obtained without detection of any amino‐difluoroalkylated by‐products (**160**, Scheme 40: 26 examples, 30–86% yields). The developed protocol was applied to the oxy‐difluoroalkylation of substrates using other difluoroalkyl reagents such as BrCF_2_PO(OEt)_2_, bromodifluoroacetamides, and 2‐BTSO_2_CF_2_H. The authors suggested a mechanism involving the intermediacy of CF_2_CO_2_Et radicals *via* reductive quenching of *[Ru(bpy)_3_]^2+^ and oxidation of DABCO for the difluoroalkylation/carboxylative cyclization process.

### 1.5 Difluoroalkylation of *sp* ^2^ Carbon Atoms in Arenes and Heteroarenes

Transition metal photocatalysis can also be considered as a very valuable tool in organic synthesis for the introduction of difluoroalkyl substituents in arenes and heteroarenes using various difluoroalkylating reagents.

**Scheme 41:**
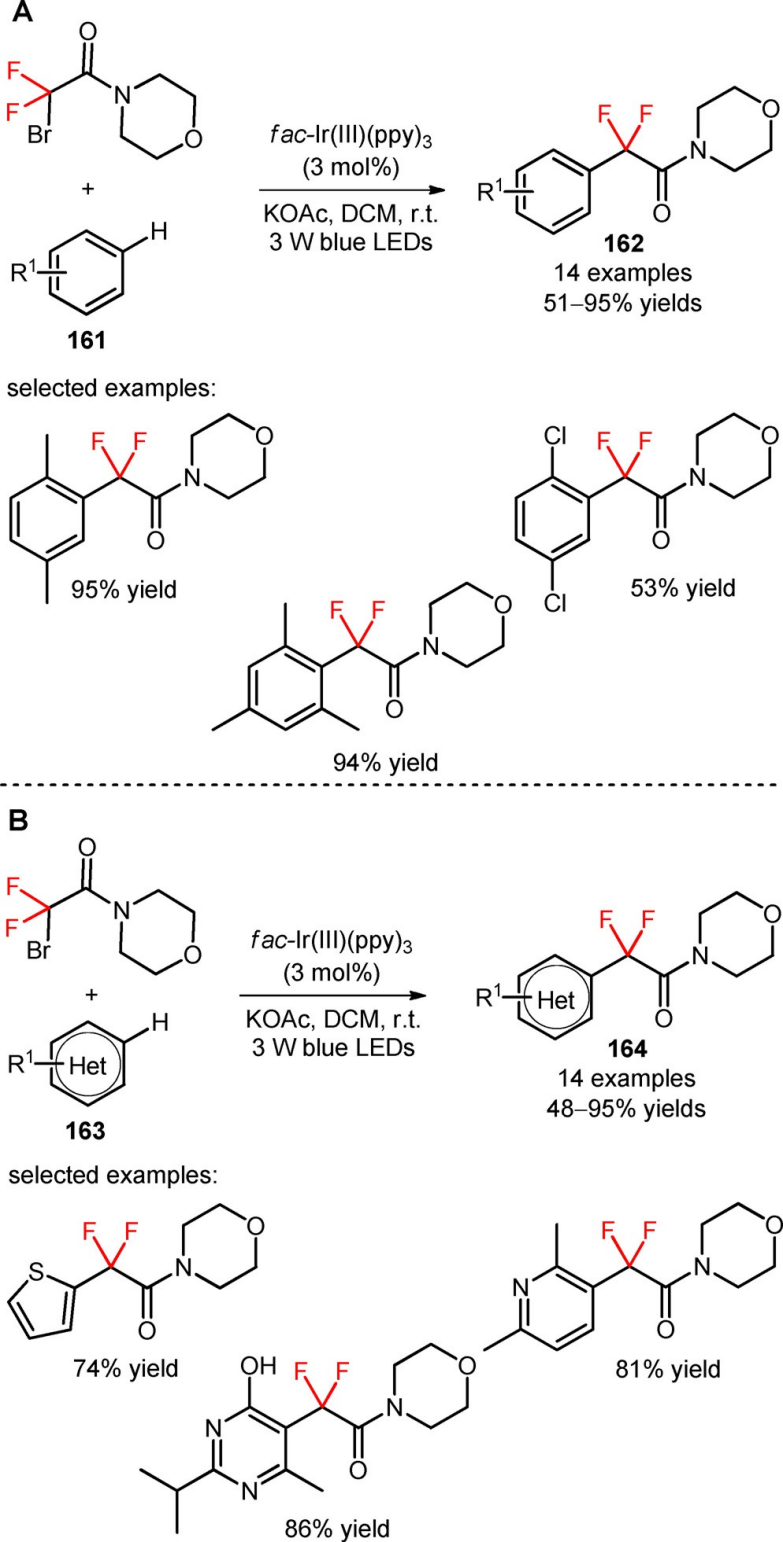
Difluoroacetamidation of unactivated arenes (**161**) and heteroarenes (**163**) with 2‐bromo‐2,2‐difluoro‐1‐morpholinoethan‐1‐one.

**Figure 7:**
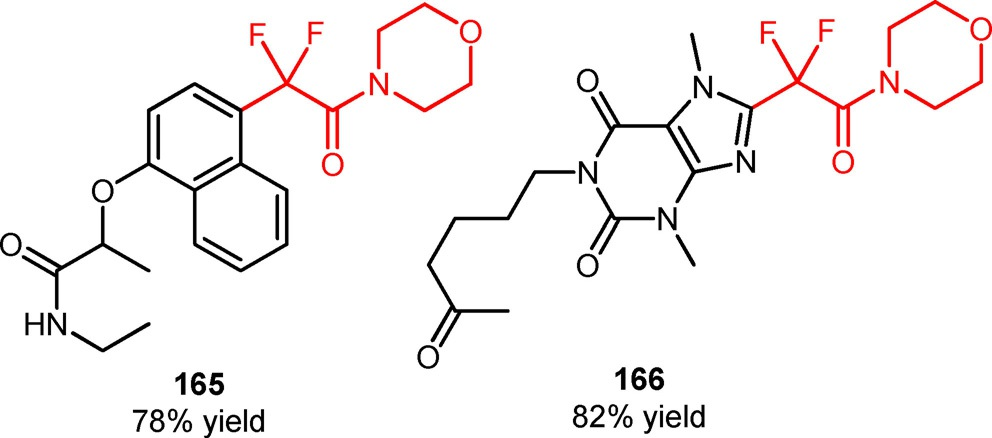
Chemical structures of difluoroacetamidated napropamide (**165**) and pentoxyfylline (**166**).

The reagent 2‐bromo‐2,2‐difluoro‐1‐morpholinoethan‐1‐one (CAS number: 149229‐27‐6) was employed by Liu and co‐workers for the visible light‐induced incorporation of difluoroalkyl moieties in unactivated arenes (**161**) and heteroarenes (**163**).^106^ An investigation of the reaction conditions using benzene as the organic substrate showed that the corresponding difluoroalkylated derivative was obtained in higher reaction yield when using DCM and the base KOAc, in the presence of *fac*‐Ir(III)(ppy)_3_ (Scheme 41). The authors found that the difluoroalkylation procedure can be extended to a wide range of mono‐, di‐, and trisubstituted arenes bearing electron‐donating and electron‐withdrawing substituents (**162**, Scheme 41A: 14 examples, 51–95% yields) and heteroarenes (pyrazines, pyridazines, pyridines, pyrimidines, and thiophenes) (**164**, Scheme 41B: 14 examples, 48–95% yields). Alternative substrates with more complex aromatic rings such as napropamide (**165**, Figure 7) and pentoxifylline (**166**, Figure 7) can also be successfully difluoroalkylated. In addition, diverse bromodifluoroacetamides possessing distinct amino groups on the amide moiety, including aniline, cyclooctanamine, cyclopropylmethanamine, piperazine, piperidine, were all compatible for the desired organic transformation. A radical‐mediated mechanism was suggested by photoluminescence quenching, electron spin resonance (ESR), spin‐trapping, and kinetic isotope effect (KIE) experiments.

**Scheme 42:**
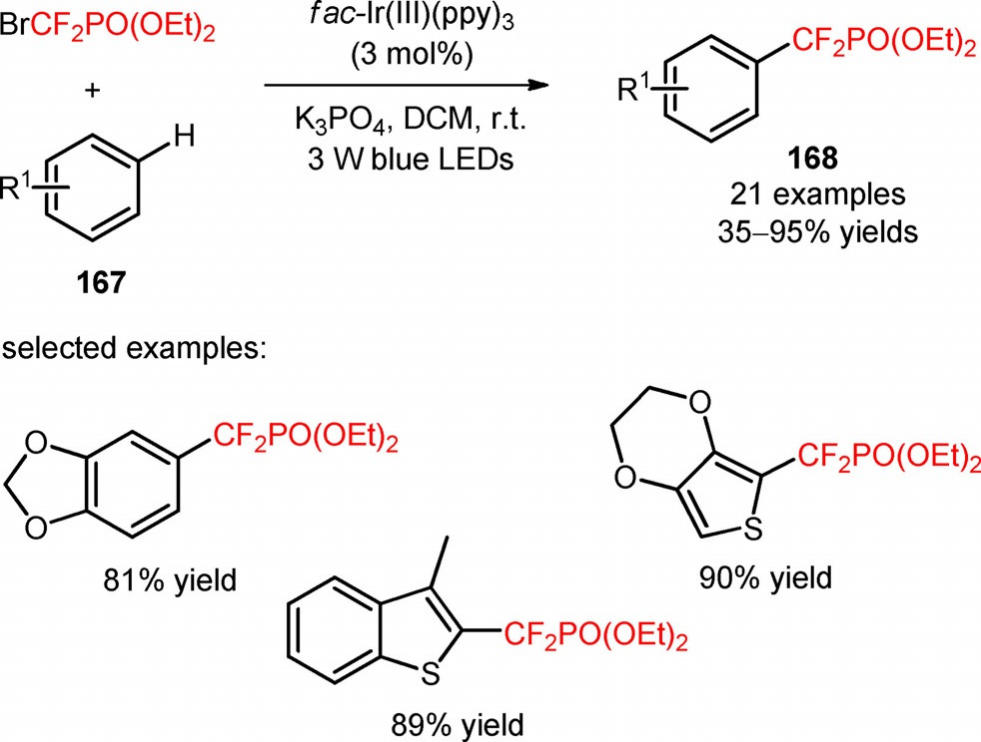
Visible light‐mediated phosphonodifluoromethylation of arenes and heteroarenes (**167**) with BrCF_2_PO(OEt)_2_.

The same group implemented the reagent BrCF_2_PO(OEt)_2_ for the introduction of CF_2_PO(OEt)_2_ moieties in arenes and heteroarenes (**167**) under irradiation with 3 W blue LEDs.^107^ Di‐ and trisubstituted arenes containing electron‐donating and electron‐withdrawing groups, and heteroarenes (benzofurans, benzothiophenes, furans, indoles, pyridines, pyrimidines, selenophenes, and thiophenes) afforded the phosphonodifluoromethylated derivatives in moderate to excellent yields (**168**, Scheme 42: 21 examples, 35–95% yields).

**Scheme 43:**
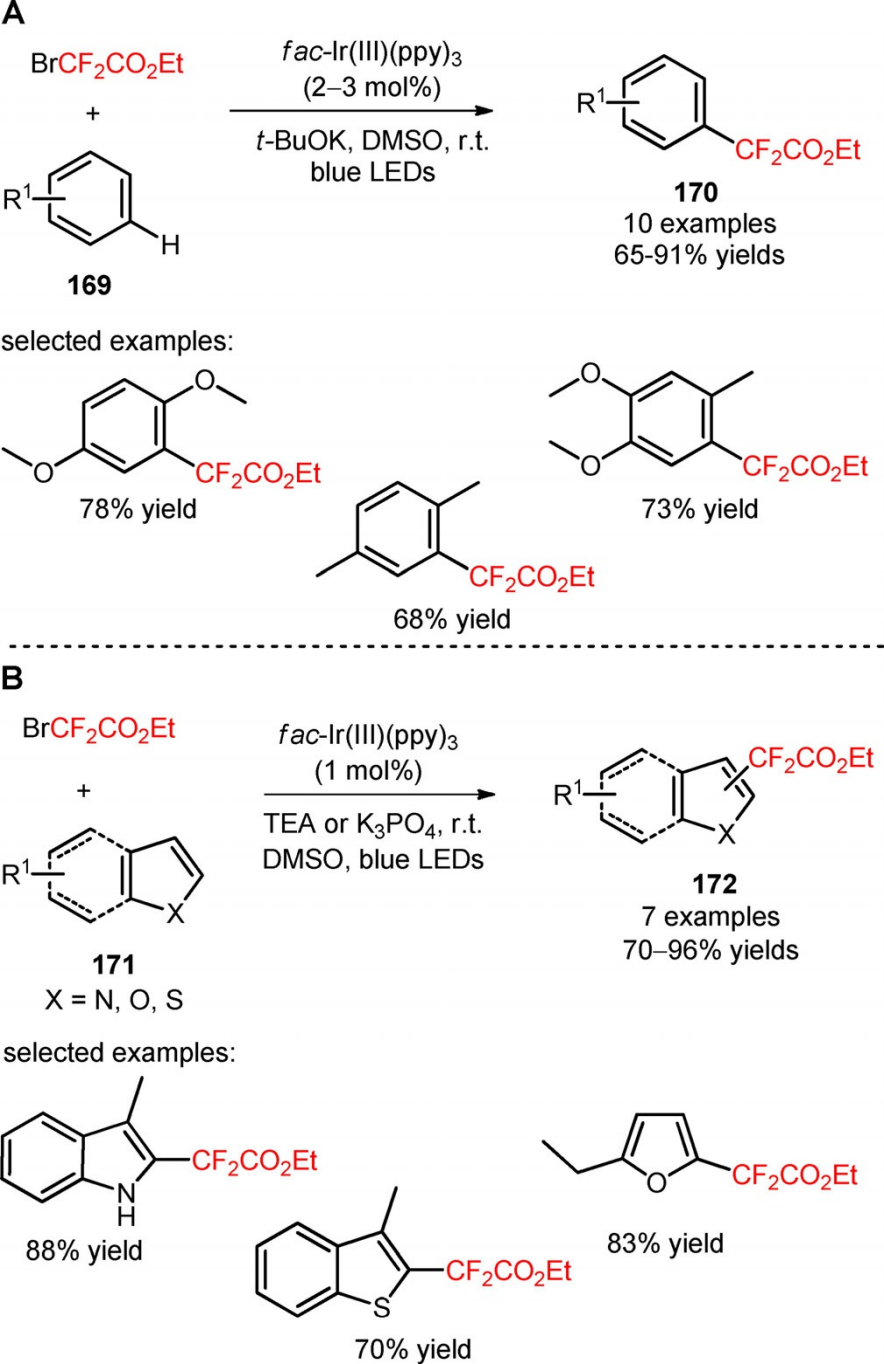
Visible light‐mediated difluoroalkylation of arenes (**169**) and heteroarenes (**171**) with BrCF_2_CO_2_Et.

An efficient photocatalytic method for the synthesis of difluoroalkylated arenes and heteroarenes was developed by Cho's group.^108^ The visible light‐promoted difluoroalkylation of unactivated electron‐rich arenes (**169**) was successfully achieved using BrCF_2_CO_2_Et in the presence of *fac*‐Ir(III)(ppy)_3_ and the base *t‐*BuOK (**170**, Scheme 43A: 10 examples, 65–91% yields). Phosphorescence quenching experiments suggested that the difluoroalkylation process was mediated by the oxidative quenching of photoexcited *fac*‐Ir(III)(ppy)_3_ and reduction of BrCF_2_CO_2_Et to CF_2_CO_2_Et radicals. Compared with electron‐rich arenes, the heteroarenes (**171**) exhibited a higher reactivity, requiring a lower amount of photocatalyst and BrCF_2_CO_2_Et, and the weak bases TEA and K_3_PO_4_. Various difluoroalkylated heteroaromatics, including benzofurans, benzothiophenes, furans, indoles, pyrroles, thiophenes can be obtained using this protocol (**172**, Scheme 43B: 7 examples, 70–96% yields).

**Scheme 44:**
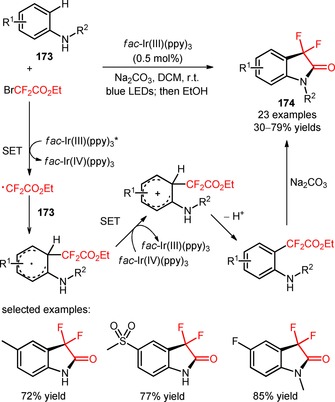
Photoinduced difluoroalkylation and intramolecular amidation of anilines (**173**) with BrCF_2_CO_2_Et and the proposed mechanism.

3,3‐Difluoro‐2‐oxindoles (**174**) were successfully synthesized through *ortho*‐difluoroalkylation of aniline derivatives (**173**) with BrCF_2_CO_2_Et and consecutive intramolecular amidation_._ 109 The difluoroalkylation/intramolecular amidation process exhibited an extensive substrate scope and a high functional group tolerance. In fact, the introduction of electron‐neutral, electron‐withdrawing, and electron‐donating substituents on the aromatic ring of the aniline derivatives was well tolerated in the desired organic transformation (**174**, Scheme 44: 23 examples, 30–79% yields). Radical‐trapping experiments with TEMPO suggested the intermediacy of CF_2_CO_2_Et radicals *via* oxidative quenching of *fac*‐Ir(III)(ppy)_3_*.

**Scheme 45:**
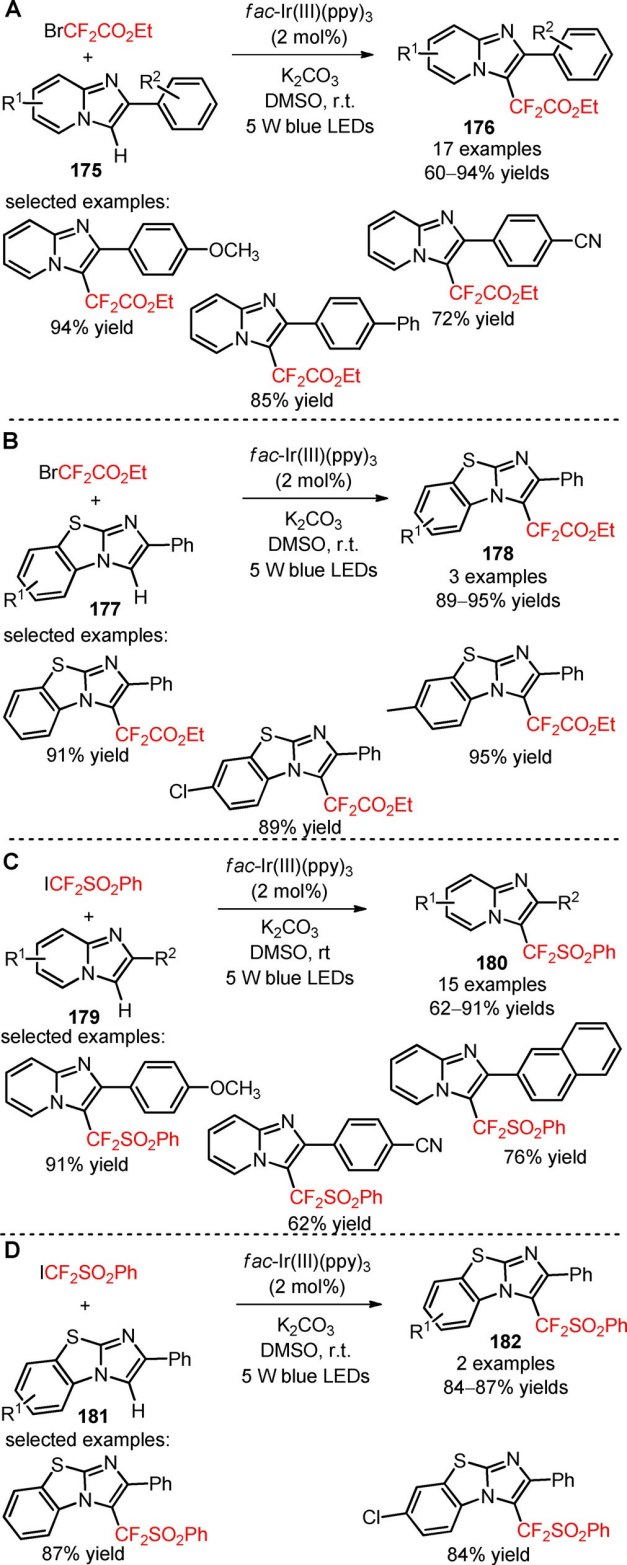
Visible light‐mediated difluoroalkylation of imidazo[1,2‐*a*]pyridines (**175**, **179**) and benzo[*d*]‐imidazo[2,1‐*b*]thiazoles (**177**, **181**) with BrCF_2_CO_2_Et and ICF_2_SO_2_Ph.

In 2017, Fu and collaborators reported two related research works concerning a methodology for the visible light difluoroalkylation of imidazo[1,2‐*a*]pyridines (**175**, **179**) using the reagents BrCF_2_CO_2_Et (Scheme 45A)^110^ and [(difluoroiodomethyl)sulfonyl]benzene (ICF_2_SO_2_Ph, CAS number: 802919‐90‐0) (Scheme 45C),^111^ in the presence of *fac*‐Ir(III)(ppy)_3_. Electron‐rich, electron‐neutral, and electron‐deficient 2‐arylimidazo[1,2‐*a*]pyridines were compatible with the difluoroalkylation process using both reagents, and gave the corresponding products in moderate to excellent yields (**176**, Scheme 45A: 17 examples, 60–94% yields; **180**, Scheme 45C: 15 examples, 62–91% yields). The functionalization of benzo[*d*]imidazo[2,1‐*b*]thiazoles (**177**, **181**) was possible under the described reaction conditions (**178**, Scheme 45B: 3 examples, 89–95% yields; **182**, Scheme 45D: 2 examples, 84–87% yields).

**Scheme 46:**
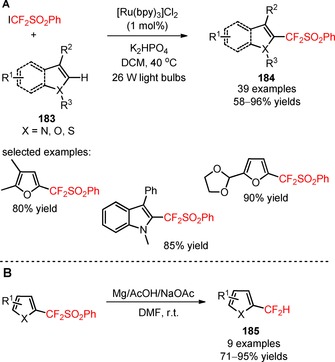
(**A**) Visible light‐driven difluoroalkylation of N‐, O‐, and S‐containing heteroarenes (**183**) with ICF_2_SO_2_Ph. (**B**) Reductive desulfonylation of CF_2_SO_2_Ph‐containing heteroarenes.

**Scheme 47:**
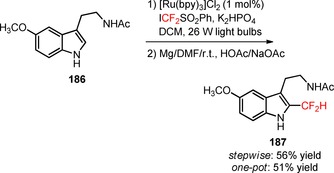
Difluoromethylation of an analogue of melatonin (**186**) in *stepwise* and *one‐pot* procedures.

The reagent ICF_2_SO_2_Ph was also efficiently applied in the difluoromethylation of N‐, O‐, and S‐containing electron‐rich heteroarenes (**183**) under irradiation with 26 W light bulbs.^112^ In the presence of [Ru(bpy)_3_]Cl_2_, electron‐rich and electron‐deficient pyrroles, furans, thiophenes, indoles and other heteroarenes containing two heteroatoms furnished the respective CF_2_SO_2_Ph‐containing heteroarenes (**184**, Scheme 46A: 39 examples, 58–96% yields). Mechanistic investigations involving radical scavengers suggested a radical‐mediated difluoroalkylation process *via* oxidative quenching of *[Ru(bpy)_3_]^2+^. Removal of the −SO_2_Ph group through reductive desulfonylation afforded the difluoromethylated derivatives (**185**, Scheme 46B: 9 examples, 71–95% yields). Interestingly, an analogue of the natural product melatonin (**186**) can be difluoromethylated in *stepwise* and *one‐pot* procedures (**187**, Scheme 47: *stepwise*: 56% yield; *one‐pot*: 51% yield).

**Scheme 48:**
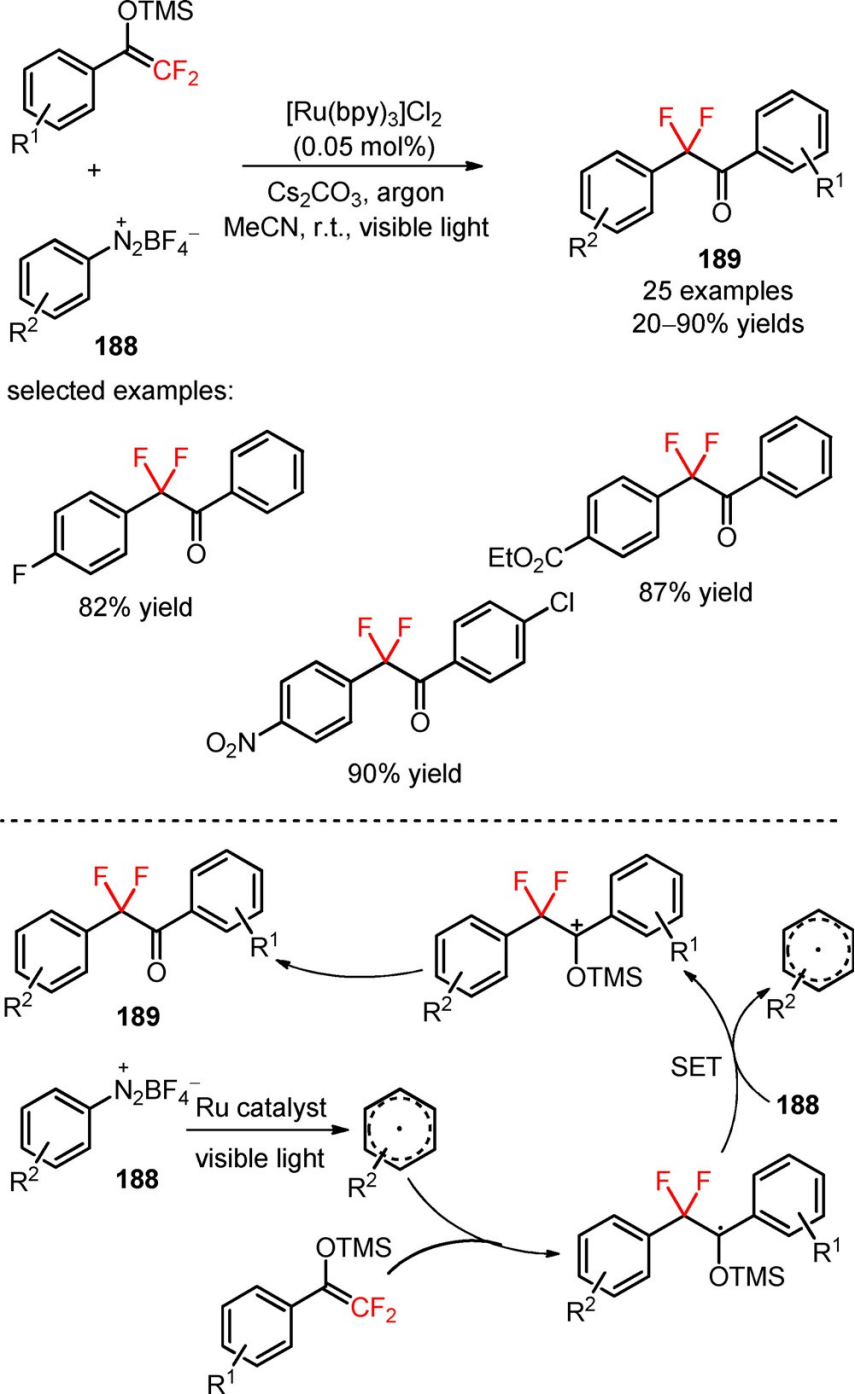
Difluoroalkylation of arenediazonium tetrafluoroborates (**188**) under visible light photoredox conditions and the proposed mechanism.

Difluoroalkylation of arenediazonium tetrafluoroborates (**188**) was achieved using α‐aryl‐β,β‐difluoroenol silyl ethers as the difluoroalkyl precursors under irradiation of visible light.^113^ The selection of the photocatalyst [Ru(bpy)_3_]Cl_2_ and the base Cs_2_CO_3_ was critical for preferential difluoroalkylation on the aromatic ring of the substrates and elimination of the unwanted difluoroalkylation of N≡N bonds (Scheme 48). A wide range of arenediazonium tetrafluoroborates bearing electron‐neutral and electron‐withdrawing groups furnished the corresponding α‐aryl‐α,α‐difluoro ketones in moderate to high yields (**189**, 25 examples, 20–90% yields). Quantum mechanical density functional theory calculations suggested a preferential mechanism involving the *in situ* generation of aryl radicals from arenediazonium tetrafluoroborates (**188**) *via* *[Ru(bpy)_3_]^2+^ species. Radical difluoroalkylation of aryl radicals, SET oxidation from another substrate, and abstraction of the trimethylsilyl group gave the respective α‐aryl‐α,α‐difluoro ketones (**189**).

**Scheme 49:**
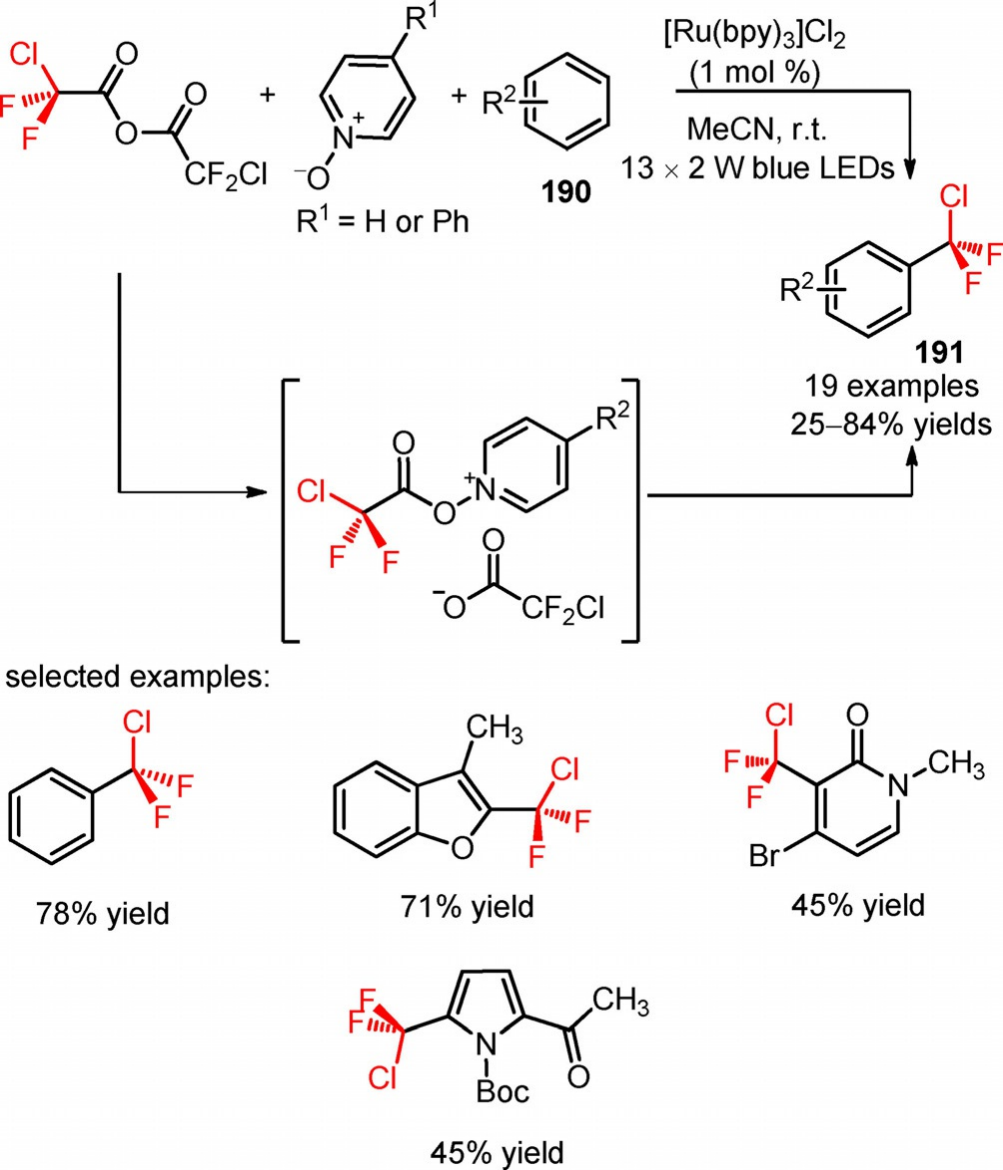
Chlorodifluoromethylation of arenes and heteroarenes (**190**) under visible light photoredox conditions.

Stephenson's group achieved the radical chloro‐difluoromethylation of arenes and heteroarenes (**190**) by *in situ* formation of a redox‐active complex resulting from the combination of heterocyclic *N*‐oxides (pyridine *N*‐oxide and 4‐phenylpyridine *N*‐oxide) with the commercially available chlorodifluoroacetic anhydride [(ClCF_2_CO)_2_O, CAS number: 2834‐23‐3], in the presence of [Ru(bpy)_3_]Cl_2._ 114 Electron‐rich heteroarenes and other pharmaceutically valuable agents with diverse functional groups were competent substrates for the desired organic transformation under both batch and flow conditions (**191**, Scheme 49: 19 examples, 25–84% yields). Interestingly, the chlorodifluoromethyl group‐containing compounds (**191**) can be used as synthetic percursors to access electron‐rich difluoromethylated arenes and heteroarenes.

**Scheme 50:**
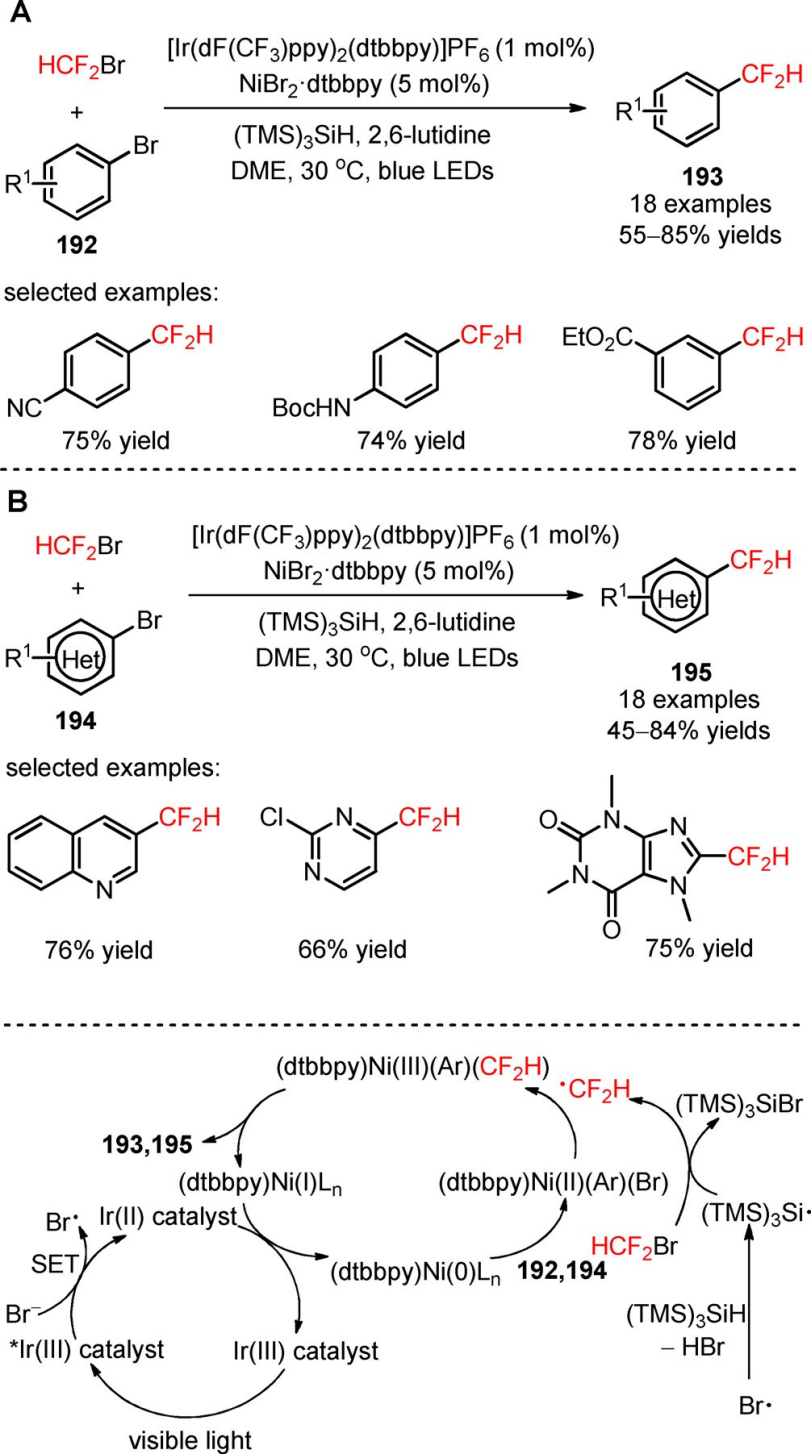
Metallaphotoredox difluoromethylation of aryl (**192**) and heteroaryl bromides (**194**) with HCF_2_Br.

**Figure 8:**
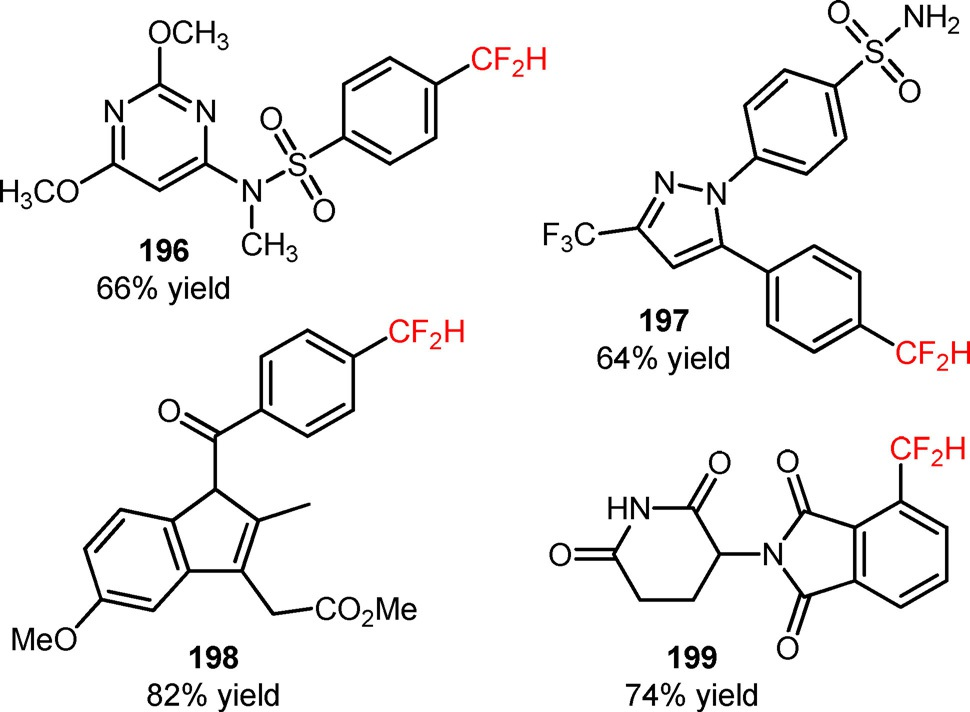
Chemical structures of difluoromethylated analogues of sulfadimethoxine (**196**), celecoxib (**197**), indomethacin (**198**), and pomalidomide (**199**).

Recently, MacMillan's group reported a convenient approach for the direct difluoromethylation of aryl (**192**) and heteroaryl bromides (**194**) by combining nickel catalysis (NiBr_2_⋅dtbbpy) with iridium photocatalysis {[Ir(dF(CF_3_)ppy)_2_(dtbbpy)]PF_6_}.^115^ Bromodifluoromethane (HCF_2_Br, CAS number: 1511‐62‐2) was employed as a direct source of CF_2_H radicals *via* a (TMS)_3_Si radical‐mediated halogen abstraction pathway. The authors suggested a mechanism of reductive quenching of [Ir(dF(CF_3_)ppy)_2_(dtbbpy)]PF_6_* and simultaneous oxidation of the bromide anion. The resulting bromine radical can then induce the formation of (TMS)_3_Si radicals that, in turn, can promote the bromine elimination from HCF_2_Br to afford the CF_2_H radicals. Concomitantly, an oxidative addition of the nickel catalyst to (hetero)aryl bromides and subsequent trapping of the CF_2_H radicals afforded a CF_2_H‐Ni(II)‐(hetero)aryl intermediate. Reductive elimination gave the respective difluoromethylated arenes (**193**) and heteroarenes (**195**). Under irradiation with blue LEDs, a variety of aryl bromides bearing electron‐withdrawing, electron‐neutral, and electron‐donating groups was compatible with the desired organic transformation (**193**, Scheme 50A: 18 examples, 55–85% yields). The developed strategy was extended to the late‐stage difluoromethylation of heteroaryl bromides (**194**), including bromo‐1*H*‐benzoimidazoles, bromo‐1*H*‐indazoles, bromopyrazines, bromopyrazoles, bromopyridines, bromopyrimidines, bromoquinolines, and bromoquinoxalines (**195**, Scheme 50B: 18 examples, 45–84% yields) and analogues of sulfadimethoxine (**196**, Figure 8), celecoxib (**197**, Figure 8), indomethacin (**198**, Figure 8), and pomalidomide (**199**, Figure 8).

### 1.6 Difluoroalkylation of *sp* Carbon Atoms in Alkynes and Biphenyl Isocyanides

Apart from the huge progress in visible light photocatalytic difluoroalkylation of *sp* ^2^‐hybridized carbon atoms in organic substrates, determined efforts have been also devoted to the exploration of efficient methodologies for the introduction of difluoroalkyl groups to *sp*‐hybridized carbon atoms, including C≡C bonds of alkynes and C≡N bonds of biphenyl isocyanides. Direct difluoroalkylation of these substrates has been demonstrated to afford synthetically useful precursors for the construction of functionalized heterocyclic molecules, such as difluoroalkylated coumarins, quinolines, and phenanthridines, under visible light irradiation.

**Scheme 51:**
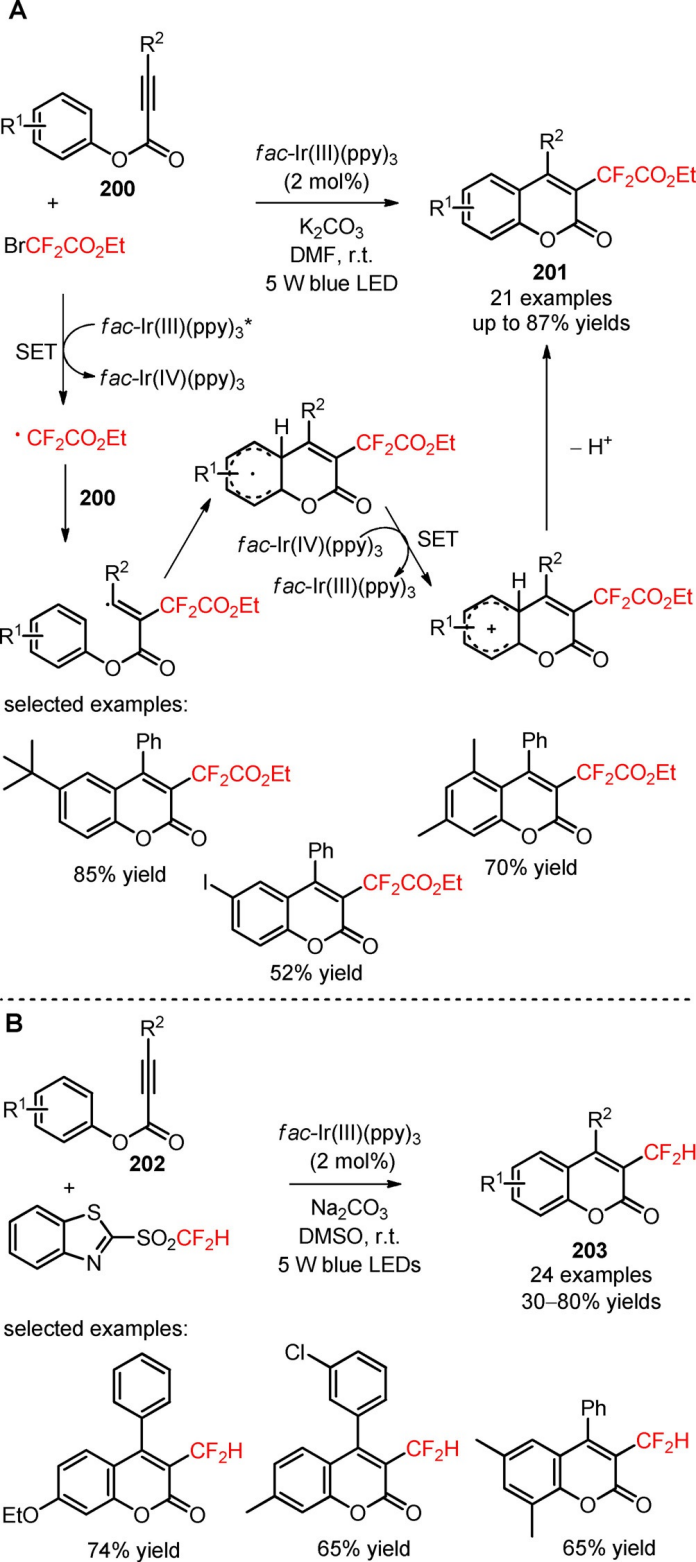
Visible light‐mediated radical difluoroalkylation/intramolecular annulation of aryl 3‐phenylpropiolates (**200**, **202**) with BrCF_2_CO_2_Et (**A**) and 2‐BTSO_2_CF_2_H (**B**).

In 2015, Ji and collaborators described a protocol for the visible light‐mediated difluoroalkylation of aryl 3‐phenylpropiolates (**200**) with BrCF_2_CO_2_Et and subsequent construction of a coumarin ring.^116^ In the presence of *fac*‐Ir(III)(ppy)_3_, the radical difluoroalkylation/intramolecular annulation of a broad scope of aryl 3‐phenylpropiolates bearing electron‐donating and electron‐withdrawing substituents in the aromatic rings gave the corresponding 3‐difluoroalkylated coumarins (**201**, Scheme 51A: 21 examples, up to 87% yields). A mechanism of oxidative quenching of *fac*‐Ir(III)(ppy)_3_* and subsequent CF_2_CO_2_Et radical‐mediated difluoroalkylation of C≡C bonds of the substrates (**200**) was proposed. Difluoromethyl‐containing coumarins were also achieved *via* the photoinduced installation of CF_2_H groups in aryl 3‐phenylpropiolates (**202**) using 2‐BTSO_2_CF_2_H (**203**, Scheme 51B: 24 examples, 30–80% yields).^117^

**Scheme 52:**
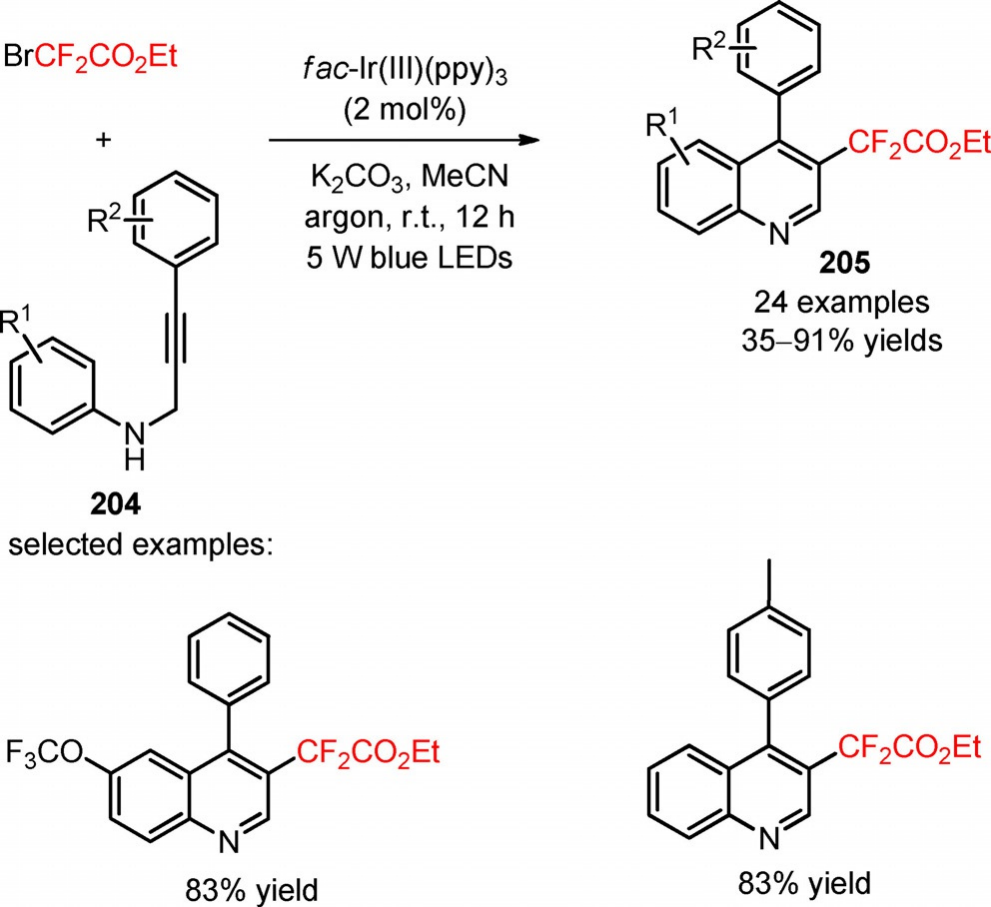
Photoinduced cascade difluoroalkylation/cyclization of *N*‐propargyl aromatic amines (**204**).

Recently, Sun and co‐workers described a methodology for the visible light‐induced radical difluoroalkylation of *N*‐propargyl aromatic amines (**204**) with the reagent BrCF_2_CO_2_Et and consecutive cyclization to form a quinoline ring.^118^ A large diversity of 3‐difluoroalkylated quinolines bearing electron‐withdrawing and electron‐donating substituents on the aniline and benzene rings was effectively obtained in moderate to high yields (**205**, Scheme 52: 24 examples, 35–91% yields).

**Scheme 53:**
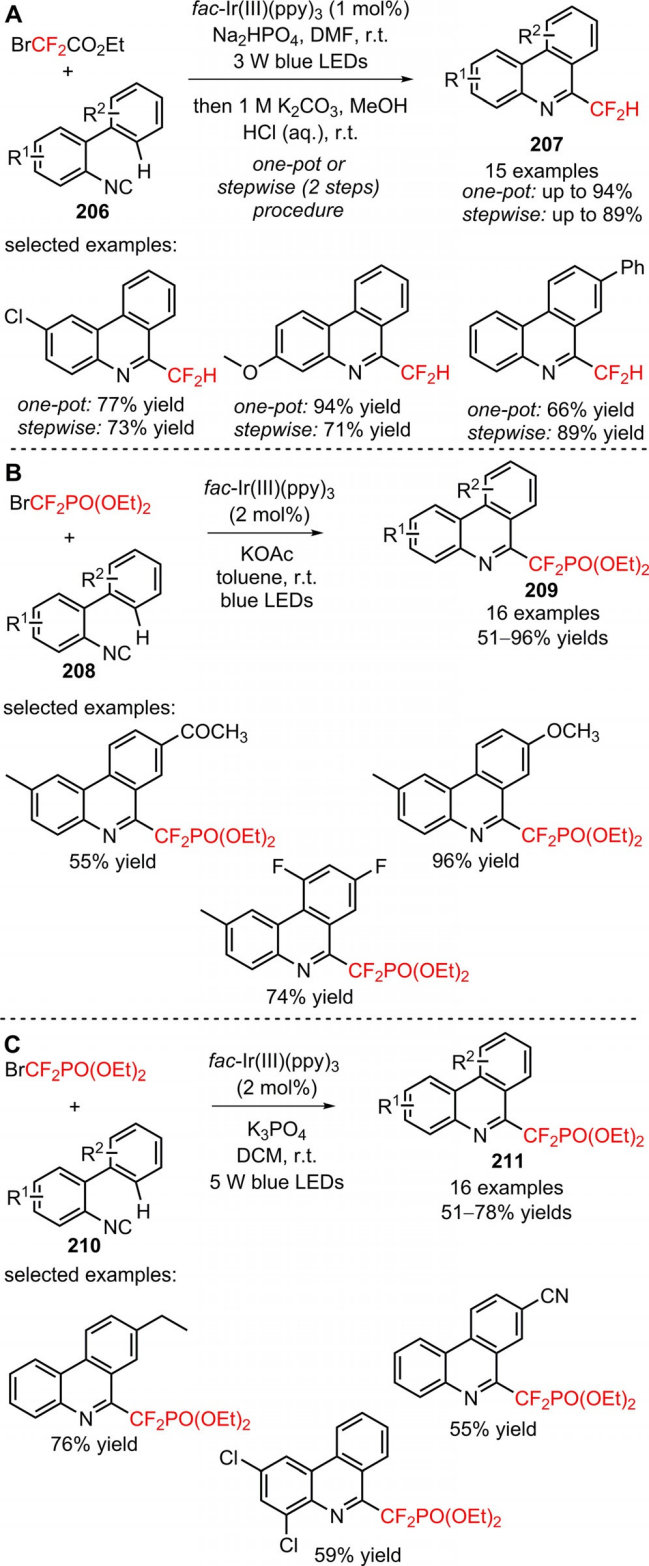
Visible light‐mediated difluoroalkylation of biphenyl isocyanides (**206**, **208**, **210**) reported by Yu (**A**), Liu (**B**), and Wang (**C**) groups.

In 2014, Yu and co‐workers developed a *stepwise* procedure for the preparation of difluoromethylated phenanthridine derivatives (**207**) involving the difluoroalkylation/radical cyclization of biphenyl isocyanides (**206**) with BrCF_2_CO_2_Et and subsequent decarboxylation under basic conditions.^119^ The difluoromethylation of the substrates took place upon visible light irradiation in the presence of *fac*‐Ir(III)ppy_3_, together with KHPO_4_ in DMF at room temperature. After the radical‐induced difluoroalkylation, the ester functionality was removed *via* saponication and subsequent acid‐mediated decarboxylation. The authors investigated the influence of a *one‐pot* procedure on the overall efficiency of difluoromethylation of biphenyl isocyanides (**206**). They found that a *one‐pot* procedure could afford a range of electron‐deficient and electron‐rich phenanthridine derivatives (**207**, Scheme 53A: 15 examples; *one‐pot*: up to 94%; *stepwise*: up to 89%) with reaction yields comparable to those of the *stepwise* methodology, and could be easily scaled up.

Later, two independent papers published by the Liu^120^ and Wang^121^ groups have shown the successful utilization of BrCF_2_PO(OEt)_2_ in the phosphono‐difluoromethylation of biphenyl isocyanides (**208**, **210**) under visible light photoredox conditions. A variety of biphenyl isocyanides bearing electron‐donating and electron‐withdrawing substituents attached to the aromatic rings was efficiently converted into the respective phosphonodifluoromethylated phenanthridines (**209**, Scheme 53B: 16 examples, 51–96% yields; **211**, Scheme 53C: 16 examples, 51–78% yields).

**Scheme 54:**
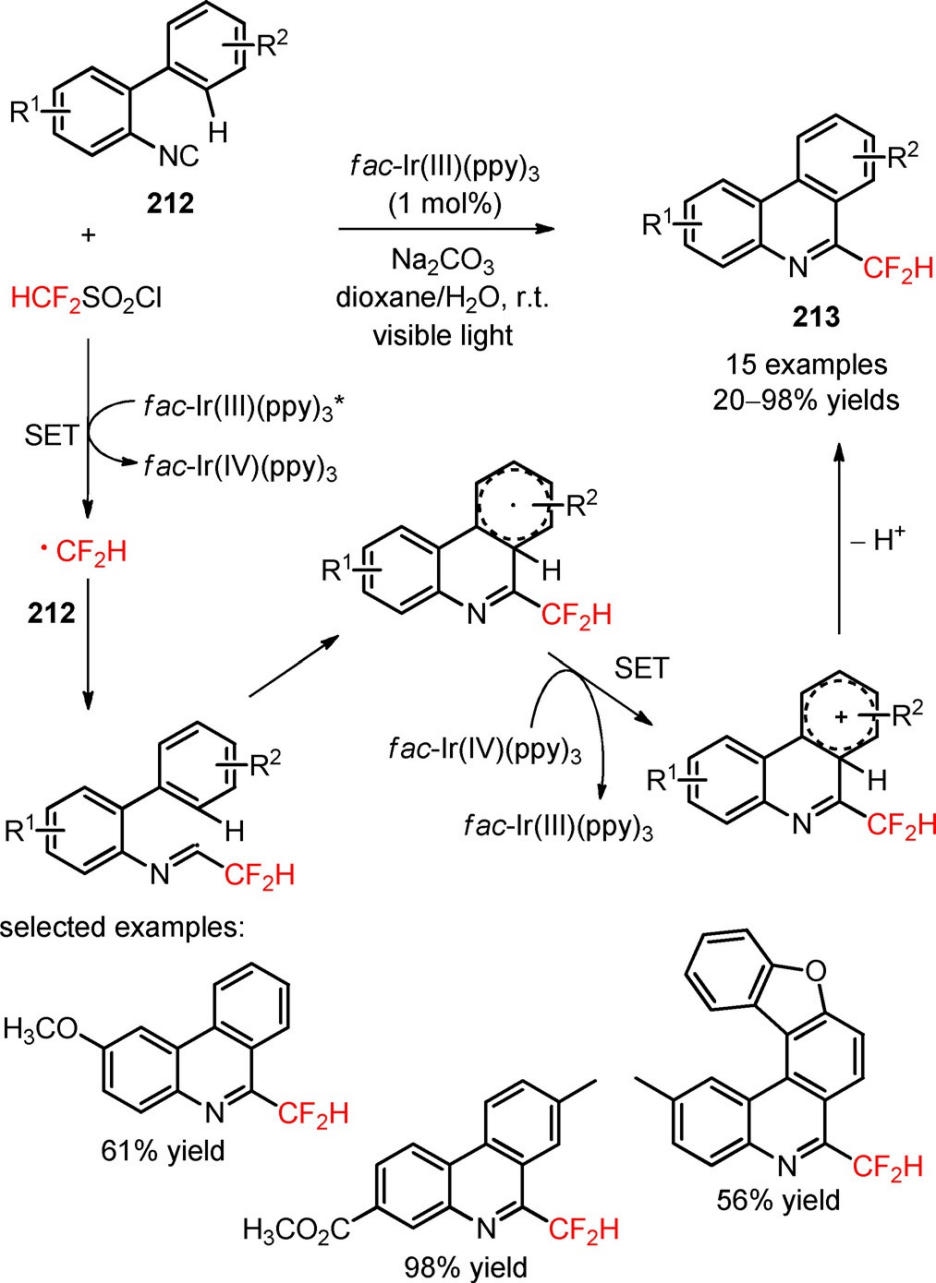
Photoinduced difluoromethylation of biphenyl isocyanides (**212**) and the suggested mechanism.

The visible light‐mediated installation of CF_2_H groups into biphenyl isocyanides (**212**) was reported for the first time by Dolbier and collaborators using the reagent HCF_2_SO_2_Cl.^122^ This method enabled the synthesis of difluoromethylated phenanthridines (**213**), excluding the need of a *stepwise* procedure involving the conversion of other *gem*‐difluoroalkyl groups into a difluoromethyl group. An improved reactivity for difluoromethylation of the substrates was accomplished using wet dioxane and K_2_HPO_4_, in the presence of *fac*‐Ir(III)(ppy)_3_ (Scheme 54). Substrates with electron‐donating and electron‐withdrawing substituents in both aromatic rings were efficiently converted into the respective difluoromethylated phenanthridines (**213**, 15 examples, 20–98% yields). Other radical fluoroalkyl precursors, in particular PhCF_2_Br and CH_3_CF_2_SO_2_Cl, were compatible with the developed methodology. The authors suggested a mechanism for the formation of phenanthridine scaffold involving the generation of CF_2_H radicals *via* oxidative quenching of *fac*‐Ir(III)(ppy)_3_* and radical addition to the C≡N bond of biphenyl isocyanides (**212**). Subsequent cyclization onto the aromatic ring, oxidation *via fac*‐Ir(IV)(ppy)_3_, and base‐assisted deprotonation gave the final products (**213**). Similar mechanistic pathways have been proposed to the difluoroalkylation reactions described in the Scheme 53.

**Scheme 55:**
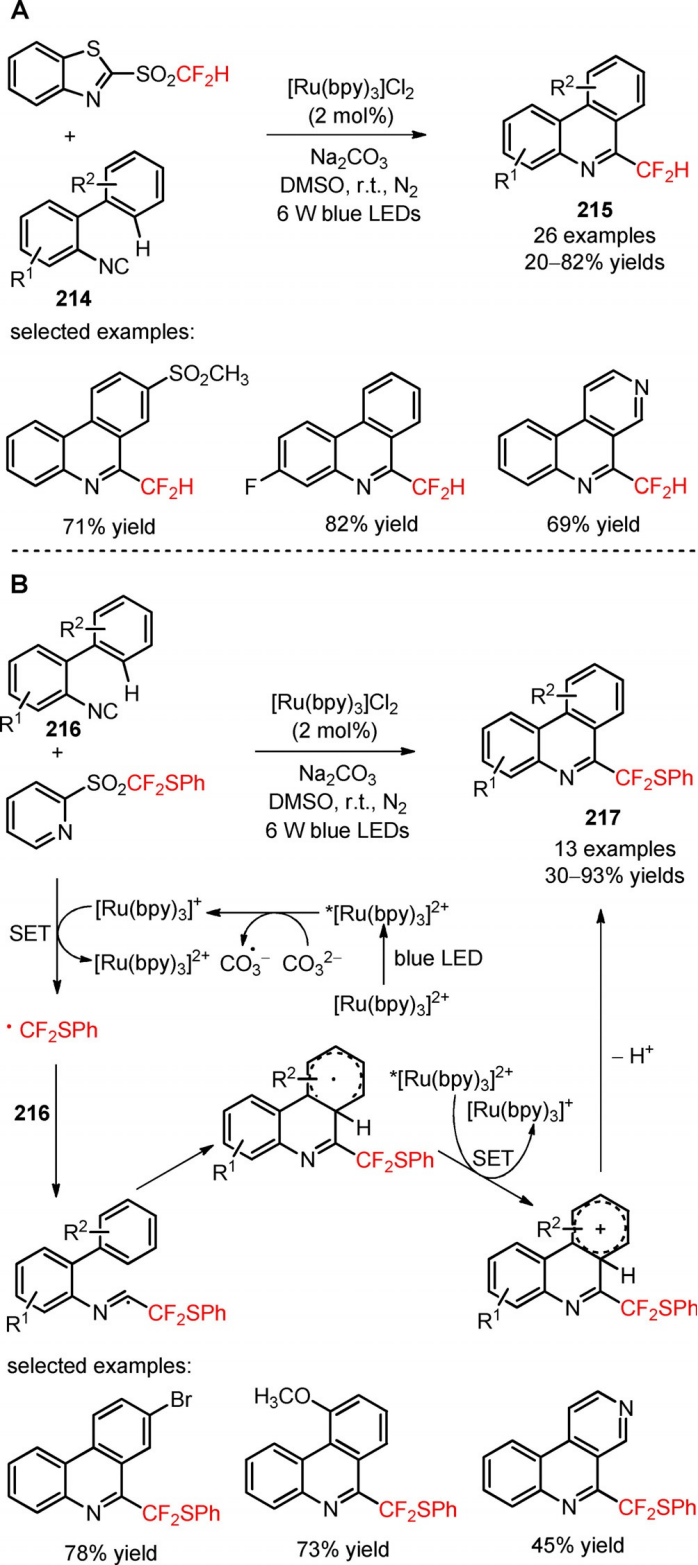
Visible light‐promoted difluoromethylation of biphenyl isocyanides (**214**, **216**) with 2‐BTSO_2_CF_2_H (**A**) and 2‐PySO_2_CF_2_SPh (**B**).

Difluoroalkyl sulfones, including 2‐BTSO_2_CF_2_H^123^ and 2‐PySO_2_CF_2_SPh,^124^ have also been implemented in the synthesis of difluoromethylated (**215**) and arylthio‐difluoromethylated phenanthridines (**217**), respectively, from biphenyl isocyanides (**214**, **216**), under irradiation with 6 W blue LEDs. Optimal conditions for the difluoromethylation and arylthiodifluoromethylation were achieved by combining the photocatalyst [Ru(bpy)_3_]Cl_2_ with the base Na_2_CO_3_ and DMSO. Biphenyl isocyanides bearing electron‐donating and electron‐withdrawing substituents on the aromatic rings were suitable substrates for the desired transformation (**215**, Scheme 55A: 26 examples, 20–82% yields; **217**, Scheme 55B: 13 examples, 30–93% yields). The developed methodology was also extended to other fluoroalkyl sulfones containing 1,1‐difluoroethyl (−CF_2_CH_3_), (phenyl)difluoromethyl (−CF_2_Ph), (benzoyl)difluoromethyl (−CF_2_COPh), and arylthiodifluoromethyl (−CF_2_SAr) moieties. Luminescence quenching experiments suggested a mechanism of difluoroalkylaytion mediated by reductive quenching of *[Ru(bpy)_3_]^2+^ *via* oxidation of CO_3_^2−^.

### 1.7 Difluoroalkylation of SH‐ and OH‐Containing Substrates

**Figure 9:**
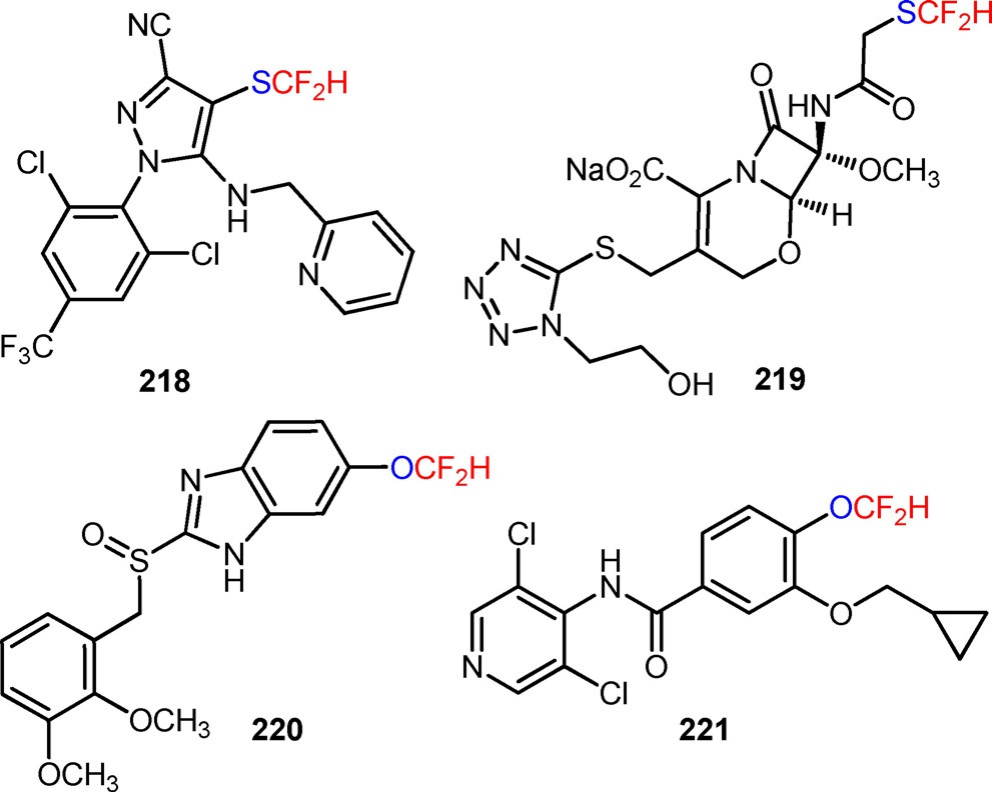
Chemical structures of SCF_2_H‐ [pyriprole (**218**) and flomoxef sodium (**219**)] and OCF_2_H‐containing bioactive molecules [pantoprazole (**220**) and roflumilast (**221**)].

Transition metal‐photoinduced difluoroalkylation of *sp* ^2^‐ and *sp*‐hybridized carbon atoms has been widely described under the scope of this review. On the other hand, the difluoroalkylation of other groups, in particular SH and OH groups, by visible light photoredox catalysis has been underdeveloped. The resulting −SCF_2_H and −OCF_2_H substituents have emerged as important functional groups in bioactive molecules, including the pyriprole (**218**, Figure 9), flomoxef sodium (**219**, Figure 9), pantoprazole (**220**, Figure 9), and roflumilast (**221**, Figure 9). Just recently in 2017, the Fu and Qing groups reported on the use of fluoroalkylating agents for the synthesis of difluoroalkyl (thio)ether derivatives under photoredox conditions.

**Scheme 56:**
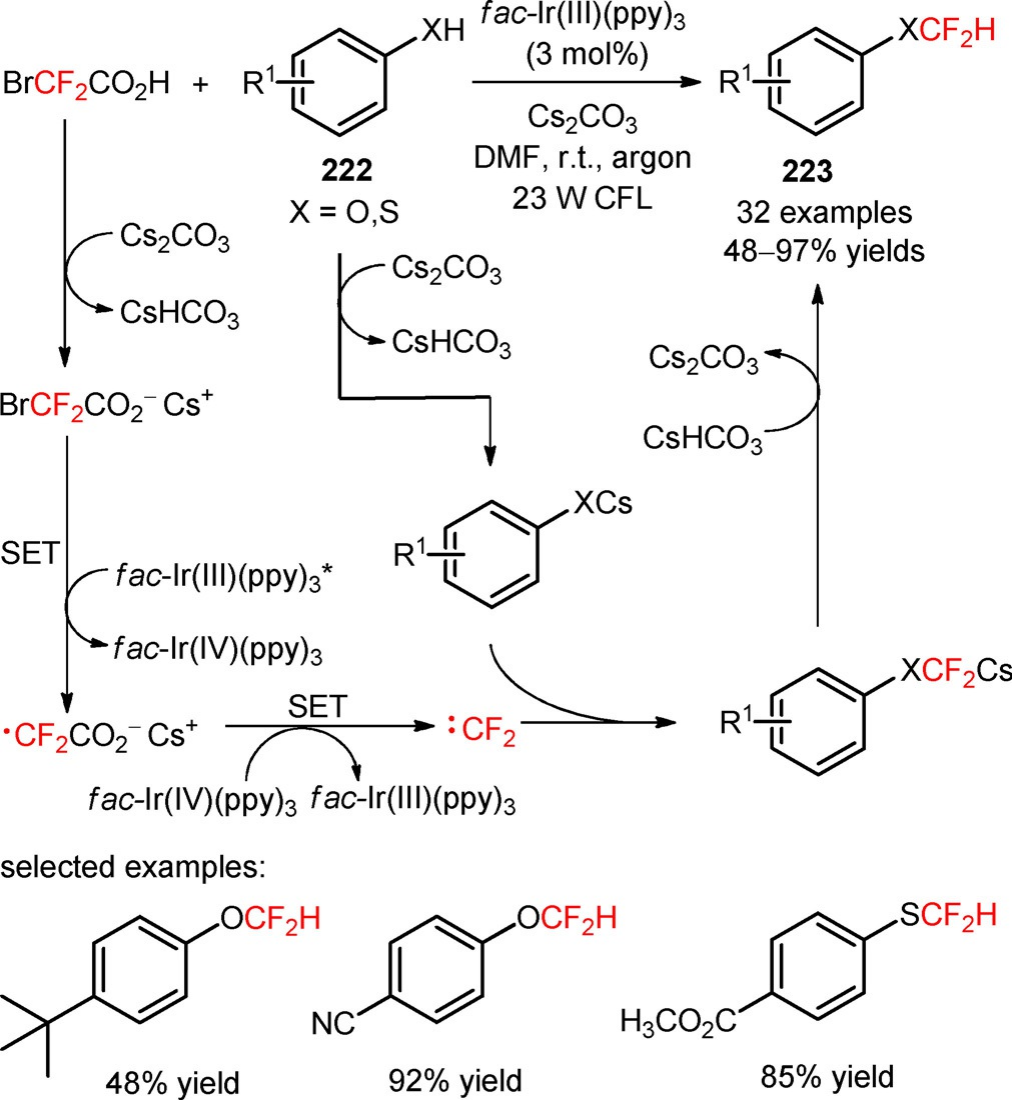
Photoinduced difluoromethylation of phenols and thiophenols (**222**) with BrCF_2_CO_2_H in the presence of *fac*‐Ir(III)(ppy)_3_ and the proposed mechanism.

The commercially available bromodifluoroacetic acid (BrCF_2_CO_2_H, CAS number: 354‐08‐5) was employed in the difluoromethylation of phenols and thiophenols (**222**) under visible light with a 23 W CFL.^125^ Screening of photocatalysts, bases, and solvents showed that the combination of *fac*‐Ir(III)(ppy)_3_, Cs_2_CO_3_, and DMF, respectively, was appropriate for the efficiency of difluoromethylation process (Scheme 56). Phenols and thiophenols possessing electron‐donating and electron‐withdrawing groups gave the corresponding difluoromethylated (thio)ethers (**223**, 32 examples, 48–97% yield). The protocol was also applied to other substrates, such as heteroaryl alcohols and heteroaryl thiols. The authors hypothesized a mechanism for the difluoromethylation involving the generation of a difluorocarbene (:CF_2_) intermediate *via* oxidation of a radical carbanion intermediate resulting from the reaction between BrCF_2_CO_2_H and Cs_2_CO_3_, and subsequent reduction. Concurrently, reaction of the phenol and thiophenol derivatives (**222**) with Cs_2_CO_3_ provided ArXCs (X=O, S) and CsHCO_3_. The reaction of ArXCs with :CF_2_ and subsequent treatment with CsHCO_3_ provided the difluoromethylated (thio)ether derivatives (**223**).

**Scheme 57:**
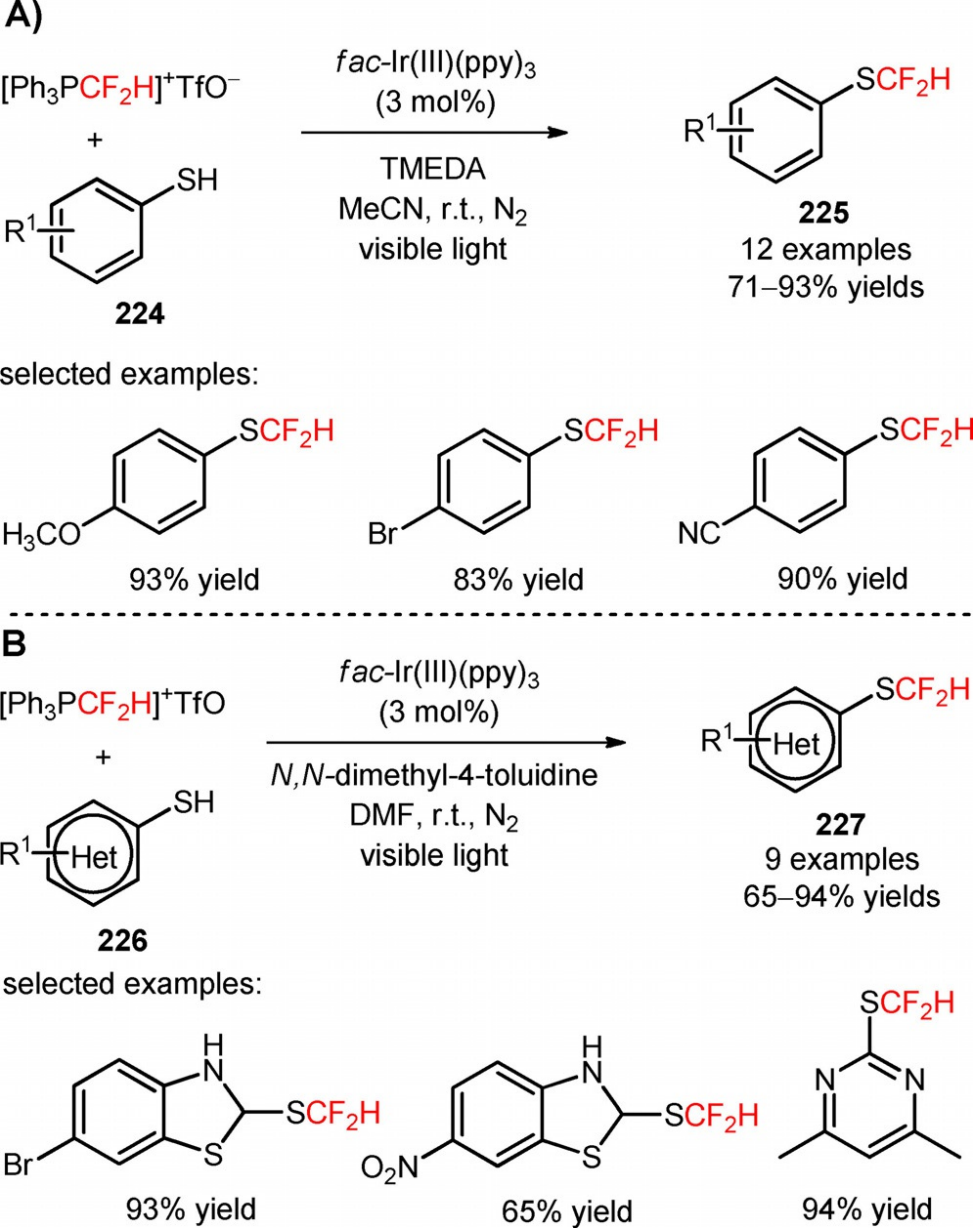
Visible light‐induced photocatalytic difluoromethylation of thiophenols (**224**) and heteroaryl thiols (**226**) using the reagent [Ph_3_PCF_2_H]^+^TfO^−^.

**Scheme 58:**
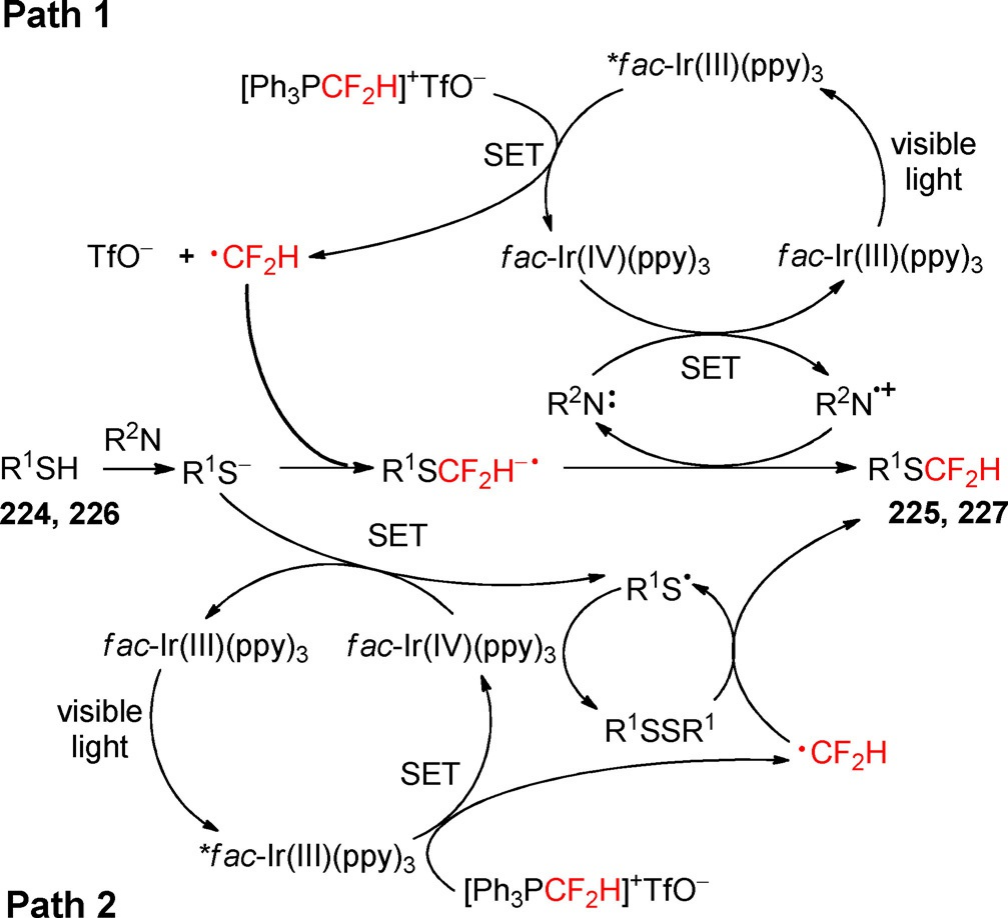
Proposed mechanistic pathways for the photoinduced difluoromethylation of thiophenols (**224**) and heteroaryl thiols (**226**).

In addition, the reagent (difluoromethyl)triphenylphosphonium triflate ([Ph_3_PCF_2_H]^+^TfO^−^) was applied in the radical difluoromethylation of thiols (**224**, **226**) under irradiation with visible light.^126^ Apart from [Ph_3_PCF_2_H]^+^TfO^−^, the selection of photocatalyst *fac*‐Ir(III)(ppy)_3_ and TMEDA as the base was critical for the success of the difluoroalkylation reactions (Scheme 57). A large variety of thiophenols possessing electron‐neutral, electron‐donating, and electron‐withdrawing substituents and heteroaryl thiols including benzo[*d*]thiazole‐2‐thiols, 2‐thiopyridines, 4‐thiopyridines, and 2‐thiopyrimidines yielded the corresponding difluoromethylated thioethers (**225**, Scheme 57A: 12 examples, 71–93% yields; **227**, Scheme 57B: 9 examples, 65–94% yields). Interestingly, an excellent S/X (X=O, N) chemoselectivity of the difluoromethylation process was observed. Two plausible pathways were proposed for the radical difluoromethylation of thiols (Scheme 58). Electrophilic addition of CF_2_H radicals to the thiolates and subsequent oxidation afforded the corresponding products (Path 1). Alternatively, the thiolate can be oxidized by *fac*‐Ir(IV)(ppy)_3_ to a sulfur radical and then converted into the disulfide derivative. Difluoromethylated thioethers (**225**, **227**) were obtained from the reaction between the CF_2_H radicals and the disulfide (Path 2). The latter mechanism was considered the more likely pathway due to the observed chemoselective S‐difluoromethylation.

## 2 Organic Photocatalyzed Difluoroalkylation Reactions

Over the last years, we have seen promising advances in the application of transition metal photocatalysts in the late‐stage difluoroalkylation of organic substrates. Nevertheless, metal‐catalyzed processes usually present a number of drawbacks, including the use of expensive metal catalysts, their potential toxicity, and in a few cases problems related to the elimination of the metal catalyst at the end of a reaction.^35^,^127^,^128^ The development of synthetic approaches using organic dyes and other metal‐free organic compounds has been regarded as an attractive alternative to transition metal complexes in photoredox catalysis. These organic photocatalysts are typically less expensive, less toxic, and easy to handle.^129^,^130^ The development of metal‐free catalyzed protocols is highly desirable in the pharmaceutical industries in order to restrict the maximal amount of transition metal impurities used in the production of pharmaceuticals. A survey of difluoroalkylation reactions that make use of organic photocatalysts is presented in this section.

**Scheme 59:**
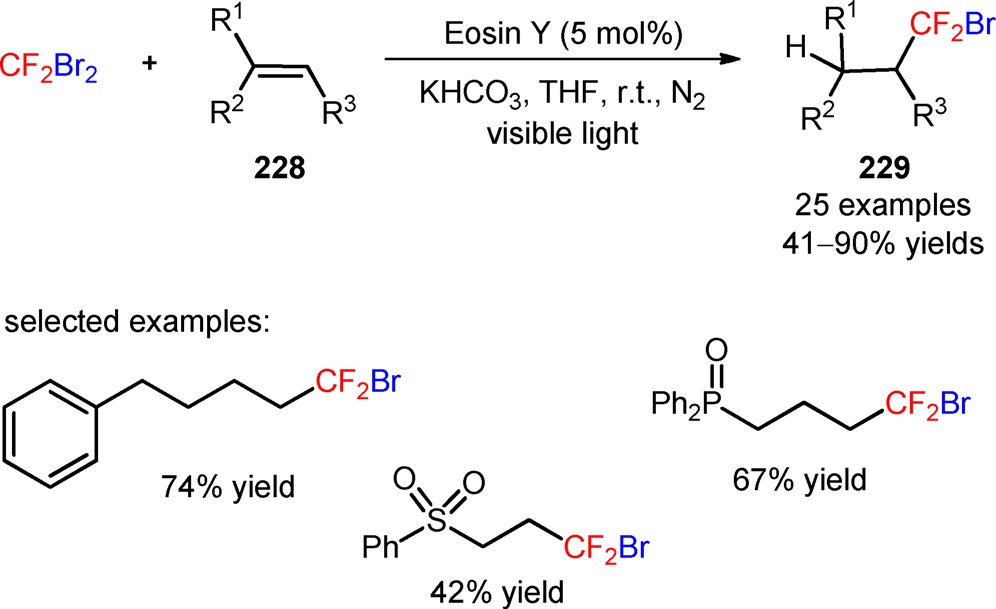
Photoinduced hydro‐bromodifluoromethylation of alkenes (**228**) with CF_2_Br_2_.

**Figure 10:**
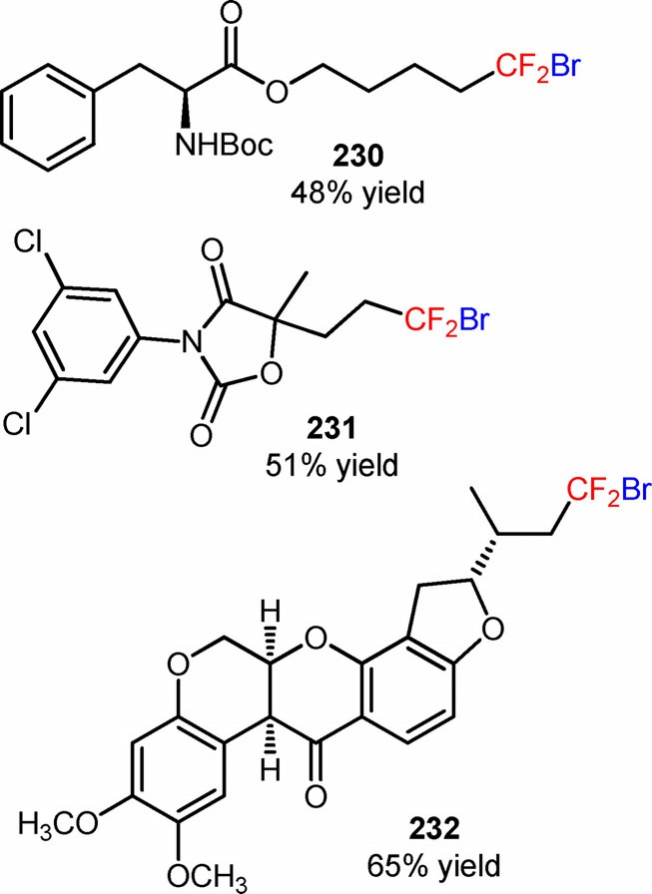
Chemical structures of hydro‐bromodifluoromethylated l‐phenylalanine derivative (**230**), vinclozolin (**231**), and rotenone (**232**).

**Scheme 60:**
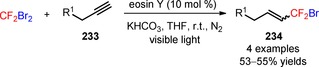
Photoinduced hydro‐bromodifluoromethylation of alkynes (**233**) with CF_2_Br_2_.

A photoinduced hydro‐bromodifluoromethylation of alkenes (**228**) was reported by Qing and co‐workers^131^ using dibromodifluoromethane (CF_2_Br_2_, CAS number: 75‐61‐6) as difluoroalkylating reagent and eosin Y as photocatalyst.^127^ Under irradiation with visible light, the combined use of THF as hydrogen atom donor with the additive KHCO_3_ was appropriate to achieve selectivity of the hydro‐bromodifluoromethylation process and to minimize the competitive bromine trapping after the bromodifluoromethylation process (Scheme 59). Mono‐ and disubstituted alkenes possessing a wide range of functional groups, including aldehydes, alkyl and allylic alcohols, amides, carboxylic acids, esters, ethers, halides, ketones, nitriles, nitro groups, phosphine oxides, sulfones, can be converted into the corresponding hydro‐bromodifluoromethylated products (**229**, 25 examples, 41–90% yields). Remarkably, the developed protocol for hydro‐bromodifluoromethylation can be extended to more complex alkenes, including an l‐phenylalanine derivative (**230**, Figure 10), vinclozolin (**231**, Figure 10), and rotenone (**232**, Figure 10), as well as alkynes **233** (**234**, Scheme 60: 4 examples, 53–55% examples). Mechanistic studies suggested the involvement of photoexcited eosin Y in the reduction of CF_2_Br_2_ with concomitant generation of bromodifluoromethyl (CF_2_Br) radicals. Subsequent radical addition to the alkenes (**228**) and hydrogen abstraction from THF afforded the hydro‐bromodifluoromethylated products (**229**). Labelling experiments with THF‐*d*_8_ corroborated the suggested mechanism of hydrogen abstraction.

**Scheme 61:**
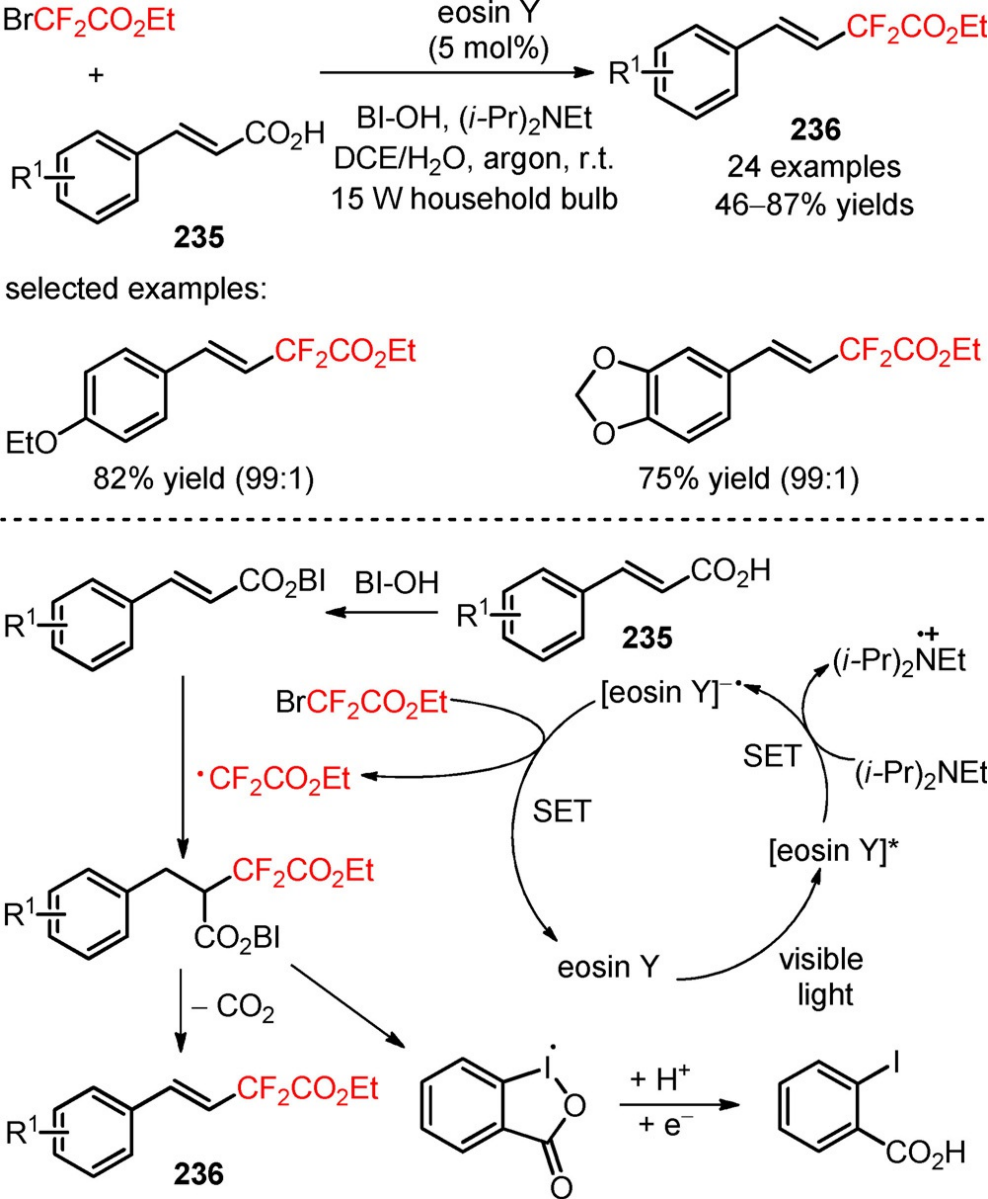
Visible light‐mediated decarboxylative difluoromethylation of α,β‐unsaturated carboxylic acids (**235**) and the proposed mechanism.

Eosin Y was also effectively employed as a metal‐free photocatalytic system for the decarboxylative difluoroalkylation of α,β‐unsaturated carboxylic acids (**235**) with BrCF_2_CO_2_Et under mild conditions.^132^ The use of the hypervalent iodine reagent 1‐hydroxy‐3‐oxobenziodoxole (BI‐OH) enabled the decarboxylative difluoromethylation of the substrates *via* activation of the carboxylic acid group. Under irradiation with a 15 W household bulb, an improved reaction efficiency was accomplished using the solvent mixture DCE/H_2_O and the reducing agent (*i‐*Pr)_2_NEt (Scheme 61). A wide range of (*E*)‐difluoroalkylated styrenes possessing both electron‐donating and electron‐withdrawing groups at *meta*‐ and *para*‐positions on the aromatic ring was successfully synthesized in good yields (**236**, 24 examples, 46–87% yields). The authors suggested a reaction mechanism involving reductive quenching of the photoexcited eosin Y *via* SET oxidation of (*i‐*Pr)_2_NEt with concomitant formation of CF_2_CO_2_Et radicals. The hypervalent iodine reagent BI‐OH is incorporated into the carboxylic acid moiety of the substrates to generate a benziodoxole vinyl carboxylic acid complex. Radical difluoroalkylation to the α‐carbon atoms of the benziodoxole vinyl carboxylic acid and subsequent elimination of CO_2_ and benziodoxole radical provided the desired (*E*)‐difluoroalkylated styrenes (**236**).

**Scheme 62:**
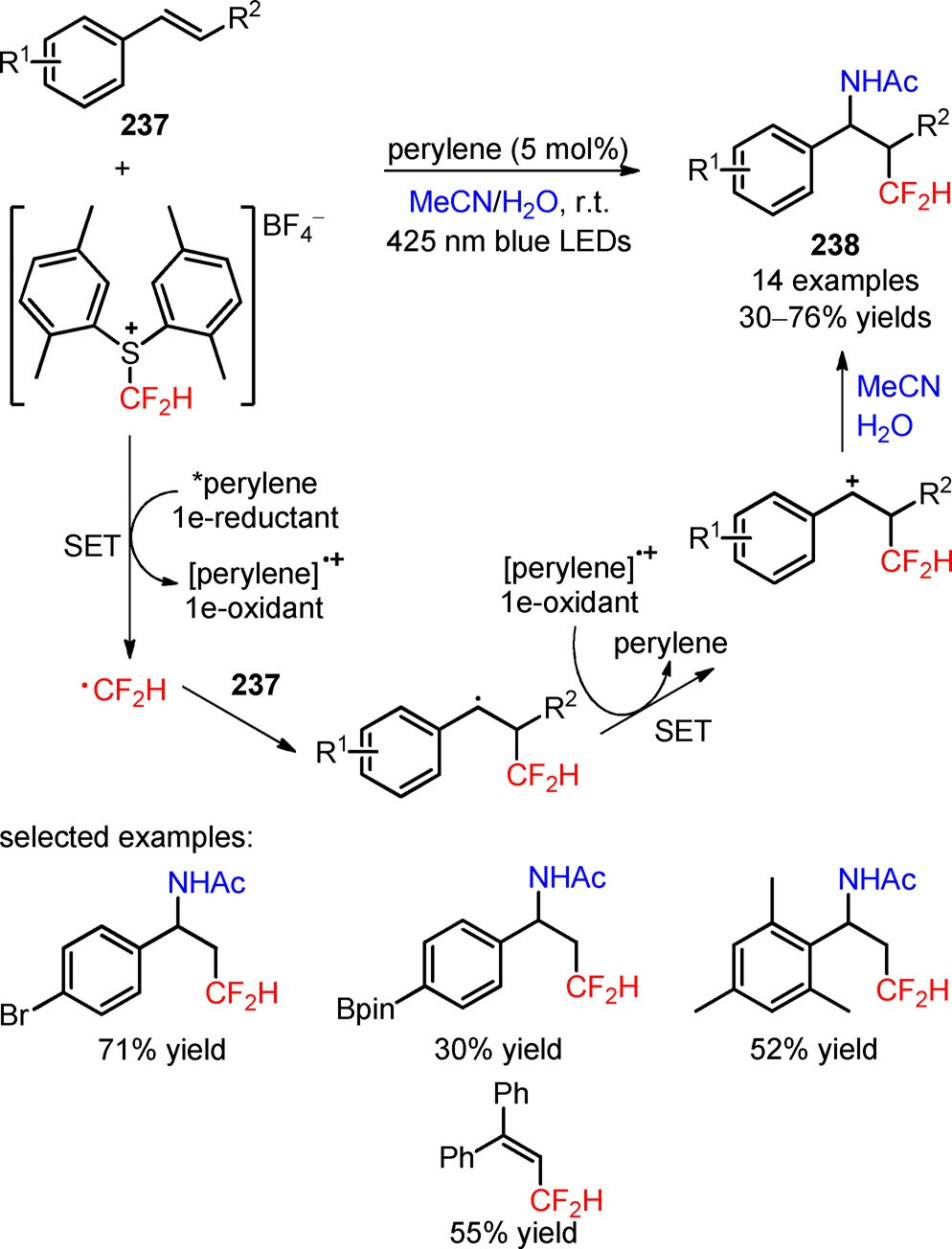
Perylene‐catalyzed amino‐difluoromethylation of styrenes (**237**) with *S*‐difluoromethyl‐*S*‐di(*para*‐xylyl)sulfonium tetrafluoroborate and the proposed mechanism.

In 2017, Akita and collaborators described the visible light‐induced amino‐difluoromethylation of styrenes, using the shelf‐stable and easy‐to‐handle difluoromethylating reagent *S*‐difluoromethyl‐*S*‐di(*para*‐xylyl)sulfonium tetrafluoroborate (CAS number: 2133476‐50‐1) and the photocatalyst perylene in the presence of MeCN and H_2_O.^133^ The presence of the two methyl groups of the *para*‐xylyl unit in the proximity of the sulfur atom of *S*‐difluoromethyl‐*S*‐di(*para*‐xylyl)sulfonium tetrafluoroborate confers easy‐handling and stability to this reagent. The amino‐difluoromethylation process exhibited a good functional group tolerance (electron‐donating and electron‐deficient groups) and afforded a variety of amino‐difluoromethylated products in moderate to good yields (**238**, Scheme 62: 14 examples, 30–76% yields). Perylene also promoted the amino‐ and chloro‐trifluoromethylation of styrenes using the Yagupolskii–Umemoto reagent and CF_3_SO_2_Cl, respectively. A plausible mechanism for the amino‐difluoromethylation of styrenes (**237**) involved the formation of CF_2_H radicals *via* oxidative quenching of photoexcited perylene and reductive cleavage of the C−S bond of the reagent. Radical addition to styrenes, oxidation, and subsequent Ritter amination with MeCN/H_2_O gave the corresponding products (**238**).

**Scheme 63:**
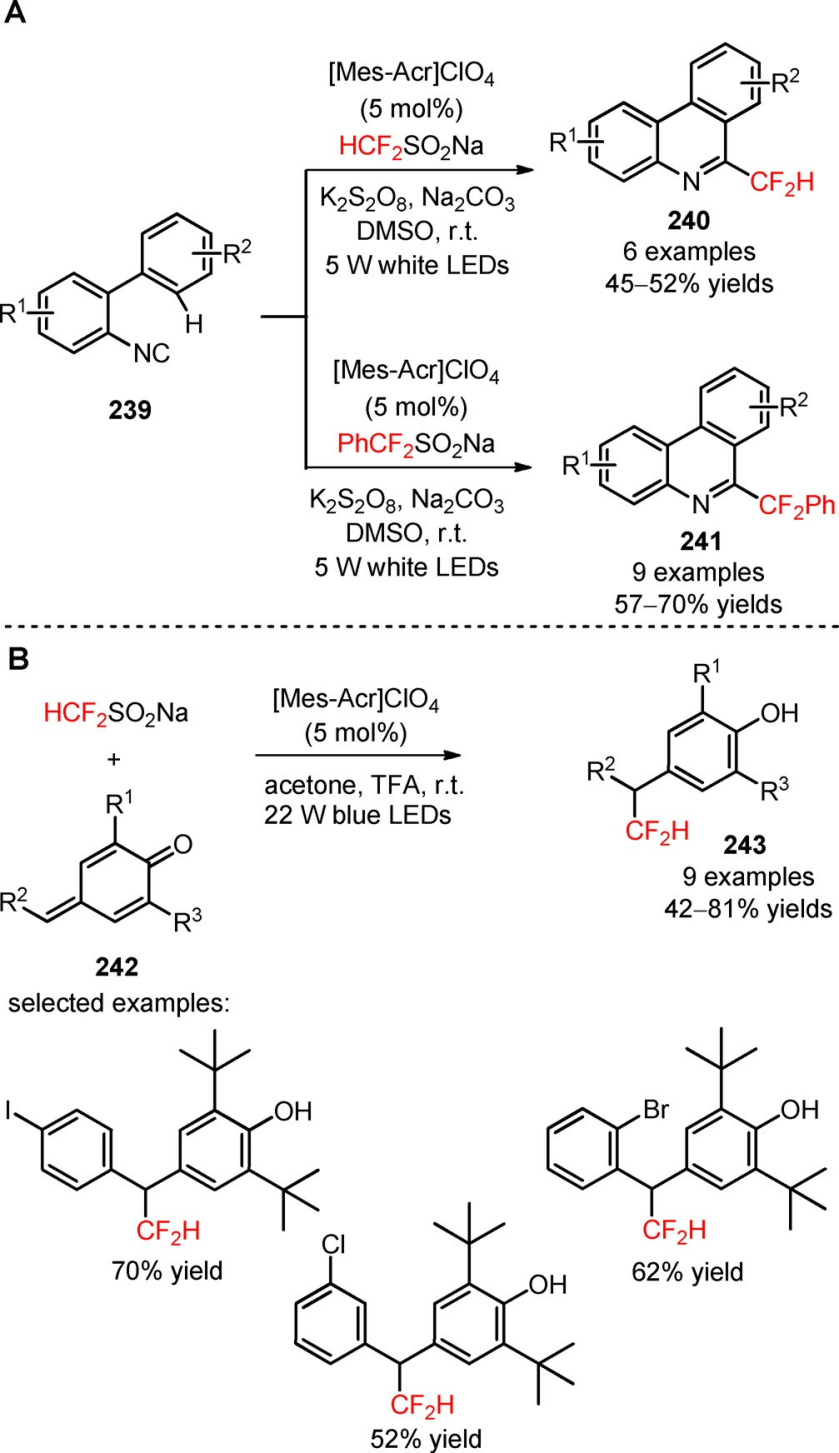
Visible light‐induced difluoroalkylation of biphenyl isocyanides (**239**) and *para*‐quinone methides (**242**).

Liu and co‐workers described a successful application of the organic photocatalyst [Mes‐Acr]ClO_4_ 134, 135, 136, 137 to the visible light‐mediated insertion of CF_2_H and CF_2_Ph radicals into biphenyl isocyanides (**239**) using sodium difluoromethanesulfinate (HCF_2_SO_2_Na, CAS number: 275818‐95‐6) (**240**, 6 examples, 45–52% yields) and sodium difluoro(phenyl)methanesulfinate (PhCF_2_SO_2_Na, CAS number: 268730‐04‐7) (**241**, 9 examples, 57–70% yields), in the presence of the oxidant K_2_S_2_O_8_ and the base Na_2_CO_3_ (Scheme 63A).^138^ The authors suggested a reductive quenching of photoexcited [Mes‐Acr]^+^ and oxidation of HCF_2_SO_2_Na and PhCF_2_SO_2_Na *via* photoexcited [Mes‐Acr] or by K_2_S_2_O_8_ to produce the CF_2_H and CF_2_Ph radicals, respectively. *para*‐Quinone methides (**242**) were also difluoromethylated using the reagent HCF_2_SO_2_Na and the photocatalyst [Mes‐Acr]ClO_4_, in the presence of TFA (Scheme 63B).^139^ Under irradiation with 9 W white LEDs, a range of difluoromethylated derivatives could be obtained in moderate to good yields (**243**, 9 examples, 42–81% yields) and no competitive nucleophilic addition of the double bond of the substrates (**242**) to the sulfonate group of the reagent was observed.

**Scheme 64:**
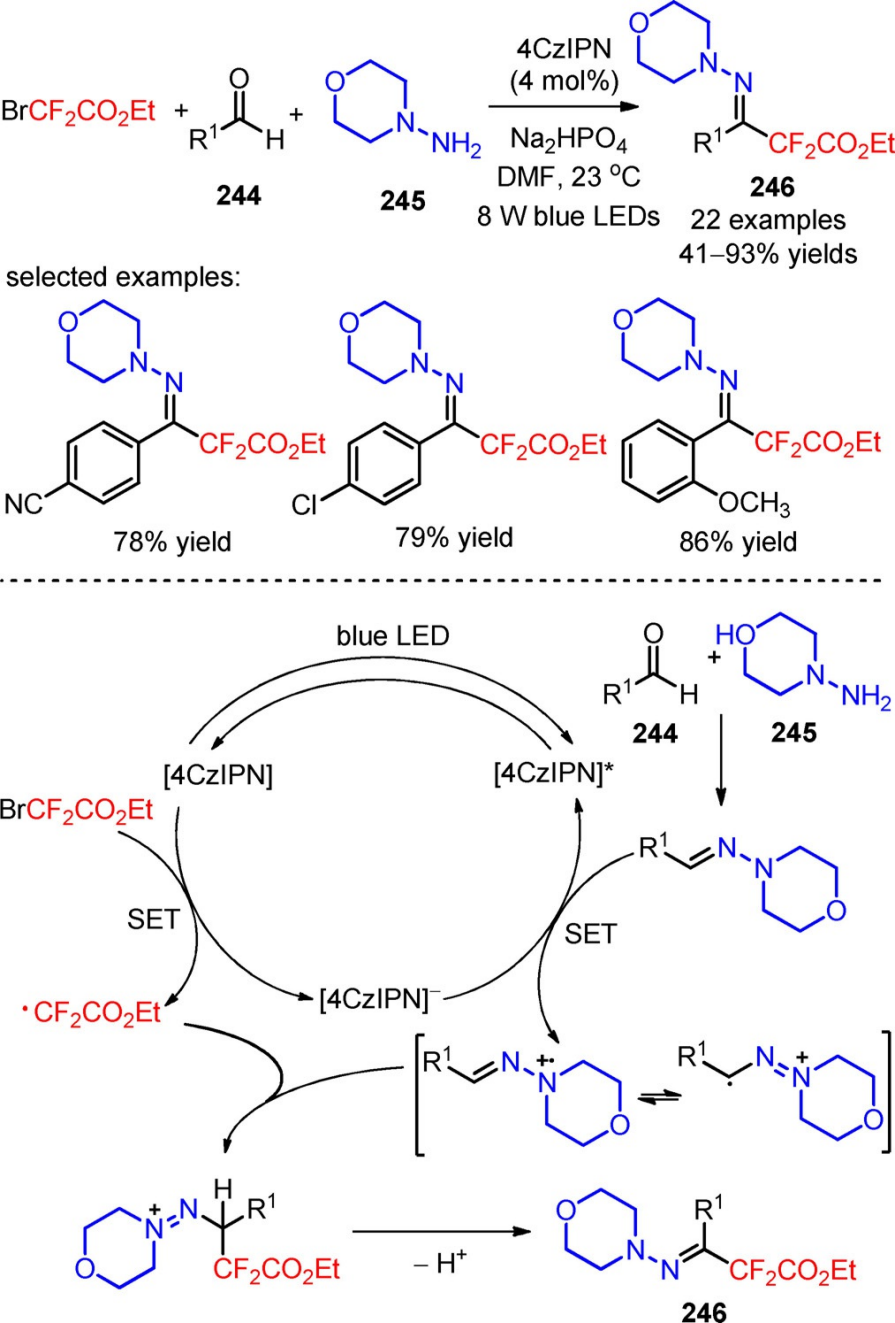
Organic dye‐catalyzed three‐component coupling of aldehydes (**244**), hydrazines (**245**) and BrCF_2_CO_2_Et, under visible light photoredox conditions.

Wang and co‐workers employed the photocatalyst 4CzIPN^140^, ^141^, ^142^ in the difluoroalkylation of aldehyde‐derived hydrazones *via* a three‐component coupling of aldehydes (**244**), hydrazines (**245**) and BrCF_2_CO_2_Et, under irradiation with 8 W blue LEDs.^143^ An investigation of the substrate scope for the difluoroalkylation process demonstrated that aldehydes bearing electron‐donating and electron‐withdrawing substituents at *ortho*‐, *meta*‐, and *para*‐positions on the aromatic ring, as well as heterocyclic and aliphatic groups were compatible substrates, providing the corresponding products in moderate to excellent yields (**246**, Scheme 64: 22 examples, 41–93% yields). This methodology can also be applied to different hydrazines and other reagents such as bromodifluoroacetamides and 2‐(bromodifluoromethyl)benzoxazole. Radical trapping and fluorescence quenching experiments suggested a mechanism involving the *in situ* coupling between aldehydes (**244**) and hydrazines (**245**), the reductive quenching of [4CzIPN]*, and the intermediacy of CF_2_CO_2_Et radicals in the difluoroalkylation process.

## 3 Concluding Remarks

The present review provides a general survey of visible light photoredox methodologies for the late‐stage installation of CF_2_FG and CF_2_H substituents into diverse families of organic substrates in the presence of distinct transition metal complexes and organic photocatalysts. In recent years, visible light photoredox catalysis has been an extensively exploited tool for promoting radical‐involved transformations, including the activation of organic substrates and further construction of the corresponding difluoroalkylated products. Most of the reported photoinduced reactions are operationally simple, are efficiently performed under mild conditions, and require minimal amounts of transition metal (0.05–7 mol%) and organic photocatalyst (4–10 mol%).

Diverse difluoroalkylating reagents have been successfully employed in the incorporation of difluoroalkyl moieties, including CF_2_CO_2_Me, CF_2_CO_2_Et, CF_2_CON(CH_2_CH_2_)_2_O, CF_2_COPh, CF_2_PO(OEt)_2_, CF_2_PO(O‐*i‐*Pr)_2_, CF_2_SPh, CF_2_Cl, CF_2_Br moieties, and some of them can undergo further chemical modifications into other CF_2_‐containing functional groups, including CF_2_H. Alternative difluoroalkyl percursors have enabled the direct introduction of CF_2_H substituents without the need of a post‐functionalization step. Interestingly, we have seen a major progress in the development of photoinduced difluoroalkylation reactions involving the use of transition metal complexes as photocatalysts, in particular by *fac*‐Ir(III)(ppy)_3_. In spite of the recent works regarding transition metal‐free difluoroalkylation reactions with organic photocatalysts, further research work in this field is still needed. In fact, the organophotocatalysis is a particular concern in the area of pharmaceutical industry, in order to minimize the utilization of transition metals.

The mechanistic pathway for most of the photoredox‐catalyzed difluoroalkylation reactions is initiated by visible light irradiation of the transition metal or organic photosensitizer at a certain wavelength and consecutive stimulation to an excited state. Excited photosensitizers may undergo an oxidative or reductive quenching, depending on the redox potential of the difluoroalkylating reagents, thus enabling the application of electron‐donor or electron‐acceptor difluoroalkylating reagents for the generation of CF_2_FG and CF_2_H radicals. The majority of the photoinduced difluoroalkylations reported in this review involves a mechanism of oxidative quenching of the photocatalysts and reduction of the difluoroalkylating reagents. The radical addition into carbon atoms of C=C, C=N, C≡C, C≡N bonds and further chemical transformations (e.g., oxidation, halogen addition, cyclization) results in the formation the corresponding CF_2_‐containing products. A variety of skeletons including alkenes, arenes, heteroarenes, α,β‐unsaturated carboxylic acids, allylic alcohols, allylic amines, unsaturated amides, alkynes, biphenyl isocyanides, and thiols have proven to be suitable for the preparation of the corresponding difluoroalkylated compounds. Furthermore, the difluoroalkylation of structurally simple starting materials provides valuable intermediates for the synthesis of highly complex and functionalized heterocycles of potential biological interest, including benzoxazines, chromones, coumarins, oxindoles, phenanthridines, polycyclic lactones, and tetralins, in a single‐step operation. Overall, the attractive characteristics of visible light photoredox reactions including environmentally benign conditions, excellent functional group versatility, and cost effectiveness will enable the application of these approaches by organic chemists in the exploration of novel methodologies for the introduction of difluoroalkyl substituents.

## Biographical Information

*Christian Lemaire* received his Ph.D. degree in 1991 from the Université de Liège, Belgium. He is a senior research scientist currently working with Professor André Luxen at the Cyclotron Research Centre (Liège, Belgium). His research is focused mainly on the development and the automation of new synthesis methods, designed according to good manufacturing practice (GMP) guidelines, for the routine production of various ^18^F‐labelled radiopharmaceuticals used in positron emission tomography (PET) imaging. He is author and co‐author of 100 articles.

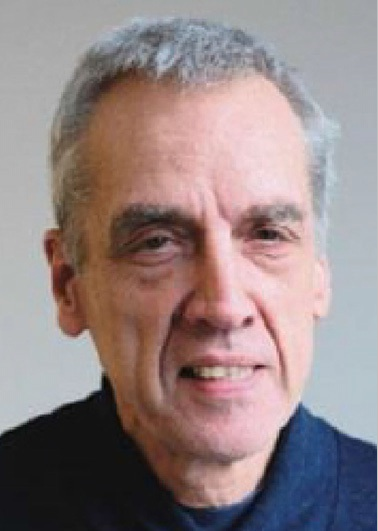

## Biographical Information

*André Luxen* is a full professor at the Université de Liège, lecturing nuclear chemistry and organic chemistry. After his Ph.D. in organic chemistry, he joined Michael E. Phelps’ group at UCLA as a post‐doc. In 1986, he was appointed as assistant professor at UCLA. He joined the University of Brussels in 1989 and established the new cyclotron centre at Erasme Hospital. From 1994 to 2017, he was the director of the CRC at the University of Liège. His research interests include mainly neuroimaging with positron emission tomography (PET) and MRI and the development of biomarkers for PET. He is author or co‐author of more than 295 articles.

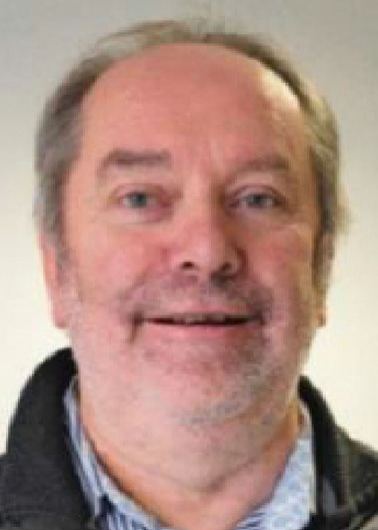

## Biographical Information

*Agostinho Lemos* obtained his M.Sc. degree in medicinal chemistry from the University of Porto, Portugal in 2015. He is currently a Ph.D. researcher in organic chemistry and radiochemistry under the doctoral program ISOTOPICS MSCA‐ITN 2016–2019 with the supervision of Prof. André Luxen. His research concerns the development of C[^18^F]FH‐containing reagents for direct difluoromethylation of organic molecules under visible light photoredox conditions.